# Human neural stem cell-derived extracellular vesicles improve cognitive function following glioma chemoradiation therapy

**DOI:** 10.1016/j.canlet.2026.218564

**Published:** 2026-05-06

**Authors:** Casey Hudson, Robert P. Krattli, Sanad M. El-Khatib, Arya R. Vagadia, An H. Do, Shreya Madan, Manal T. Usmani, Tracy Nguyen, Devyani Swami, Katja M. Piltti, Leslie M. Thompson, Brian J. Cummings, Aileen J. Anderson, Munjal M. Acharya

**Affiliations:** a Department of Anatomy & Neurobiology, University of California, Irvine, United States; b Department of Physical Medicine & Rehabilitation, University of California, Irvine, United States; c Department of Psychiatry & Human Behavior, University of California, Irvine, United States; d Department of Anatomy & Neurobiology, Department of Radiation Oncology, University of California, Irvine, United States

**Keywords:** Neural stem cells, Cognitive function, Radiation therapy, Chemotherapy, Glioma, Extracellular Vesicles

## Abstract

Cranial radiation therapy (RT) with concomitant and adjuvant temozolomide (TMZ; Stupp protocol) prolongs glioma survival but frequently results in persistent cognitive impairment. Human neural stem cell (hNSC)-derived extracellular vesicles (EVs) are a promising acellular therapy whose bioactive cargo can modulate neuroinflammation and synaptic integrity. We evaluated two EVs derived from GMP-grade hNSCs (Shef6 and UCI-191) in syngeneic glioma-bearing and non-tumor adult mice treated with fractionated cranial RT (3 × 8.67 Gy) together with concomitant low-dose (25 mg/kg) and adjuvant high-dose (66.7 mg/kg, intraperitoneal) TMZ. EV administration improved memory performance in RT-TMZ–exposed mice and, notably, Shef6-EVs also extended survival in glioma-bearing mice in the absence of chemoradiotherapy. Immunofluorescence analyses demonstrated attenuated gliosis and preservation of synaptic integrity in EV-treated RT-TMZ-exposed brains, while transcriptomic profiling identified distinct neuroprotective gene expression pathways associated with each EV source. Critically, neither Shef6 nor UCI-191 EVs diminished or interfered with the anti-tumor efficacy of RT-TMZ. These data support hNSC-derived EVs as a translational strategy to mitigate treatment-related neurotoxicity while preserving oncologic benefit in a clinically relevant glioma model.

## 1. Introduction

The US National Cancer Institute estimated more than 24,000 new central nervous system (CNS) cancer cases each year, with a 5-year survival rate of 33% [1]. Primary and metastatic brain tumors are commonly treated with the Stupp protocol, which combines fractionated cranial radiation therapy (RT) with adjuvant temozolomide (TMZ) chemotherapy [2]. Although this regimen improves survival, RT-TMZ treatment is frequently accompanied by debilitating neurocognitive sequelae that markedly diminishes patients' and survivors’ quality of life (QOL). RT-induced cognitive decline (RICD) has been linked to impaired neurogenesis, reduced dendritic complexity, elevated oxidative stress, and chronic neuroinflammation [3]. TMZ can further exacerbate CNS injury by disrupting hippocampal function, suppressing neural precursor cell proliferation, and increasing anxiety-like behaviors [4]. Together, the adverse cognitive effects of RT and chemotherapy are referred to as cancer-related cognitive impairments (CRCI) or *brain fog* [5–7]. CRCI is especially concerning in pediatric brain tumor and low-grade glioma patients, who often experience prolonged post-treatment survival [5,8,9]. Beyond survival, preservation of cognitive function may be the most critical criterion for evaluating therapeutic success in brain cancer care. However, long-term CRCI remains a major unmet clinical need, with a conspicuous lack of effective interventions. Thus, regenerative strategies that restore brain function and cognition after completion of oncologic treatment are clearly needed.

This study evaluates the translational potential of extracellular vesicles (EVs) derived from good manufacturing practice (GMP)-grade human neural stem cells (hNSC-EVs) to ameliorate CRCI in a clinically relevant mouse glioma model treated with RT-TMZ. Proliferating human neural stem cells secrete EVs that support cell-to-cell communication and maintain the neural microenvironment [10–12]. EVs carry bioactive cargo, including proteins, lipids, mitochondrial components, and nucleic acids [11]. They exhibit low immunogenicity, display a prolonged circulating half-life, cross the blood-brain barrier (BBB) [12,13], and can remain stable for up to 20 months at 4 °C [14]. EVs derived from stem or progenitor cells have been shown to promote CNS recovery after stroke and traumatic brain injury [15,16]. Our prior work using a commercially available hNSC line in an acute 9 Gy RT model demonstrated that transplanted hNSCs improved cognitive outcomes [17,18]. However, long-term follow-up revealed that only approximately 4.5% of transplanted stem cells survived at 8 months, and very few differentiated into mature, functional neurons [19]. These findings suggested that *(i)* direct cell replacement is unlikely to account for the observed neuroprotection, and *(ii)* hNSCs likely exert their therapeutic benefits by secreting trophic factors *in vivo*. Consistent with this view, studies in traumatic brain injury, stroke, and Alzheimer's disease models indicate that the functional benefits of stem cell therapy are largely mediated by EV production and release [15,16,20–25]. Indeed, intracranial administration of EVs derived from a commercially available hNSC line has been shown to confer cognitive benefit in an acute RT (9 Gy), non-cancer model [26,27]. Albeit, none of the previous neuroprotective studies tested the approach to model clinical standard of care for glioma. Building on this foundation, the present study uses a syngeneic murine glioma model and a clinically relevant Stupp protocol to test whether GMP-grade hNSC-derived EVs can mitigate CRCI and neurodegenerative sequelae *in vivo*.

## 2. Materials and methods

Details of all chemicals and materials are provided in Supplemental Resource Table.

### 2.1. Animals and groups

All animal experimental procedures were ethically approved by the Institutional Animal Care and Use Committee (IACUC) and conducted in compliance with the guidelines established by the National Institutes of Health (NIH). Twelve-week-old male and female wild-type mice (C57BL/6 J) were procured from Jackson Laboratories (RRID: IMSR_JAX:000664) and were group-housed (3–5/cage) under standard conditions, including a 12-h:12-h light-dark cycle, room temperature maintained at 20 °C ± 1, and humidity at 70% ± 10. The mice were provided standard rodent chow diet (Envigo Teklad 2020X) by the University Laboratory Animal Resources (ULAR) with ad libitum access to the chow and water. For the non-cancer experiments, animals were randomly divided into four groups (N = 5–12/group): Control + Veh (vehicle injection, received sterile PBS via retro-orbital (RO) injection, no irradiation or chemotherapy), Control + EV (received EVs via RO injection, no irradiation or chemotherapy), RT-TMZ + Veh (received irradiation and chemotherapy, sham EV injection), and RT-TMZ + EV (received irradiation and chemotherapy, EV injection). For the glioma experiments, animals were randomly divided into six groups (N = 4–12/group): Control + Veh (sham EV injection), Control + EV (received EVs), CT2A + Veh (cancer-bearing mice, sham EV injection), CT2A + EV (cancer-bearing mice, received EVs), CT2A + RT-TMZ + Veh (cancer-bearing mice treated with radiation and chemotherapy, sham EV injection), CT2A + RT-TMZ + EV (cancer-bearing mice treated with irradiation and chemotherapy, received EV injection).

### 2.2. Glioma induction, detection and growth measurements

Murine CT2A-Luc cells (Sigma-Aldrich; Cat. SCC195; RRID: CVCL_ZJ60) were cultured in monolayer with Gibco^™^ DMEM (1X), GlutaMAX^™^-I Supplement, (FisherSci; Cat. 10-569-010) and 10% Gibco^™^ FBS (FisherSci; Cat. 10-082-147). For surgical preparation, CT2A-Luc cells were first trypsinized with Gibco^™^ TrypLE^™^ Express Enzyme (FisherSci; Cat. 12-605-010), spun down at 300 G for 4 min, resuspended in Gibco^™^ Hibernate^™^-A Medium (FisherSci; Cat. A1247501) and kept on ice until implantation. Successful tumor induction in mouse subjects required a minimal concentration of 1000 cells/μL for a total dose volume of 2 μL per subject. For all cell culture procedures, the incubators were maintained at 37 °C and 5% CO_2_.

Treatment groups designated for tumor induction were initially anesthetized with 2.5% v/v isoflurane (Isopspire^™^; Dechra; Cat. 26675-46-7) delivered at 1000 cc/min ±10% using a passive scavenging anesthesia system (VetEquip; Cat. 922100). The mice were then shaved with an electric trimmer and secured to a digital mouse stereotaxic apparatus (Stoelting; Cat. 51730UD) while maintained under anesthesia with 2.0% isoflurane at 500 cc/min ±10%. The shaved scalp was sterilized using Betadine^®^ Surgical Scrub (Patterson Veterinary; Cat. 07-836-3379), followed by a Sterile Alcohol Prep Pad (FisherSci; Cat. 06-669-62), with this process repeated three times. A midline sagittal incision was made with a scalpel (#11; FisherSci; Cat. 08-927-5B), and the underlying soft tissue was cleaned with 3% hydrogen peroxide (FisherSci; Cat. H325–500) to expose bregma, which served as the reference point for stereotaxic coordinates. Tumor induction coordinates were determined as anterior-posterior (AP) +1.54 mm, medial-lateral (ML) +2.00 mm, and dorsal-ventral (DV) −3.00 mm. The AP-ML points were marked with a fine-tip pen, and a bone drill (CellPoint Scientific; Cat. 67-1024) was used to create an opening in the skull. A 10 μL Hamilton syringe (Hamilton; Cat. 80300) with a fixed needle was used to draw 2 μL of tumor cell suspension. The suspension was injected at DV −3.00 mm in two stages, with a 60-s pause following the first microliter and a 6-min pause after the second. The needle was gradually retracted per each millimeter of DV with a rest for 60 s at each depth to prevent capillary backflow of the injected cells. The incision was closed using interrupted sutures with size 6–0 polyglactin-coated material (Patterson Veterinary; Cat. 07-891-0161) and sealed with tissue adhesive (Patterson Veterinary; Cat. 07-805-5031). Post-surgery, the mice were transferred to a recovery cage on a circulating water heating pad (45 °C) and monitored until mice regained consciousness. During recovery, mice received a 1.0 mL subcutaneous injection of 0.9% saline (Patterson Veterinary; Cat. 07-800-9424). Postoperative analgesic care involved a subcutaneous injection of Buprenorphine HCl (MWI Health; Cat. 78949214), diluted to 0.05 mg/kg body weight in saline, administered immediately after surgery and again twice at 12-h intervals.

CT2A-Luc^+^ glioma progression was monitored using bioluminescence imaging (BLI) throughout the duration of the experiment. Mice were administered a 200 μL intraperitoneal injection (IP) of the bioluminescent substrate luciferin, prepared as a 15 mg/mL stock solution using D-Luciferin, sodium salt (GoldBio; Cat. eLUCNA) and Gibco^™^ DPBS (without calcium and magnesium, FisherScientific; Cat. 14-190-144). Following injection, the animals were anesthetized (isoflurane, as above) and positioned prone within the IVIS Lumina III *in vivo* Imaging System (Revvity; Cat. CLS136334). Luciferase activity expressed by CT2A-Luc^+^ cells was imaged, and quantified using Living Image Software (Revvity; Cat. 128110). The optimal time from injection to imaging was determined by performing serial imaging on two test subjects, identifying peak bioluminescent emission approximately 8–12 min post-injection. This method was applied consistently for imaging all subsequent subjects and during weekly monitoring.

### 2.3. Cranial radiation therapy

The assigned experimental groups received fractionated cranial radiotherapy (RT) approximately 7–10 days post-tumor induction and after a positive BLI signal. The mice were anesthetized as previously described. The fractionated RT was administered via the SmART^+^ irradiator (Small Animal Radiation Therapy unit, SmART; Precision X-ray, Inc.). Each fraction of 8.67 Gy dose was delivered as two equal doses of 4.34 Gy each via the sagittal plane to minimize damage to the surrounding tissue. The irradiation was attenuated using a 0.3 mm Copper filter, a 10 × 10 mm fixed collimator, and a photon spectrum of 225 kVp at 20 mA for 96 s to achieve 8.67 Gy dose. Mice received a total of three RT doses, administered every other day, for a cumulative dose of 26.01 Gy.

### 2.4. Temozolomide treatment

TMZ (3-methyl-(triazen-1-yl)-imidazole-4-carboxamide; Selleckchem; Cat. S1237) was prepared by dissolving it in filter-sterilized, warm reverse osmosis water and 2% ethyl alcohol. Two concentrations (25 mg/kg, low dose; and 66.7 mg/kg, high dose) of the drug were prepared with constant stirring at 45 °C. The drug solutions were frozen at −20 °C until usage. TMZ was administered via IP injection. Mice received a total of six IP injections over the course of two weeks, the first week was three injections of the low dose of TMZ (24-h after RT), and the second week was the high dose of TMZ (every other day).

### 2.5. Human neural stem cell culturing

The EVs used in this study were derived from two GMP-grade hNSC lines, including UCI-191 [28,29] and S6.133. hNSC (Shef6) [30–33]. All hNSC culturing was conducted in a BSL-II cell culture facility and was approved by the Institutional Biosafety Committee (IBC). The hNSCs were maintained in proliferation media as a monolayer with specific media composition for each cell line. For UCI-191 the media contained X-VIVO^™^15 Serum-Free Medium (Lonza; Cat. 04-481Q), 20 ng/mL bFGF (ThermoFisher; Cat. PHG0026), 20 ng/mL EGF (ThermoFisher; Cat. PHG0311), 10 μg/mL N2-CTS Supplement (Invitrogen; Cat. A13707–01), 10 ng/mL Leukemia Inhibitory Factor (LIF; Sigma; Cat. LIF1010), 63 μg/mL N-acetylcysteine (Sigma; Cat. A9165–5G), and 2 ng/mL Heparin (Sigma; Cat. H3149–25KU). The proliferation media for Shef6 line contained X-VIVO^™^15 Serum-Free Medium, 100 ng/mL bFGF, 100 ng/mL EGF, 10 μg/mL N2-CTS Supplement, and 10 ng/mL LIF. Cell culture media for the two hNSC lines were sterile filtered using a 0.2 μM Nalgene^™^ Rapid-Flow^™^ Sterile Filter Unit (ThermoFisher; Cat. 566-0020). The T-225 flasks used for both hNSCs were pre-coated to improve cell attachment. For Shef6, flasks were coated using CELLstart^™^ Substrate (ThermoFisher; Cat. A1014201). CELLstart^™^ was diluted with DPBS+/+ (1:100 dilution; FisherScientific; Cat. 14040-117) and was sterile filtered as previously described. Ample CELLstart^™^ was added to the flasks to cover the bottom of the flask surface and incubated overnight (37 °C and 5% CO_2_). For UCI-191, the flasks were coated first with Poly-L-ornithine (PLO; FisherScientific; Cat. A-004-C) diluted to a final concentration of 20 μg/mL using DPBS −/− (1:100 dilution). PLO was left in the flasks overnight in the incubator. The following day the flasks were treated with Laminin Mouse Protein (ThermoFisher; Cat. 23017015) diluted to a final concentration of 10 μg/mL with DPBS −/− (1:100 dilution). Laminin was left in the flasks for a minimum of 3 h in the incubator.

### 2.6. Extracellular vesicle isolation, characterization, and administration

The hNSCs released EVs into the culture media during growth; the conditioned media was continuously collected for EV isolation. After the collection of ~1.5 L of conditioned media, the EVs were isolated using an ultracentrifugation method (Optima XE-90; Beckman Coulter; Cat. A94471) [34]. The hNSC conditioned media was spun at 300 g for 5 min, the supernatant was moved to a new tube and spun at 2500 g for 20 min. The supernatant was syringe-filtered through a 0.2 μM nylon filter (ThermoFisher; Cat. 13-1001-00) and transferred to 70 mL ultracentrifuge tubes (Beckman Coulter; Cat. 355655). The 70 mL centrifuge tubes were then spun at 105,000 g for 90 min, and the resulting supernatant was discarded. At this stage, the EVs were in the pellet, which was then resuspended in DPBS −/− and transferred to 26 mL centrifuge tubes (Beckman Coulter; Cat. 355654). These tubes were spun at 105,000 g for 120 min. The resulting pellets of purified EVs were resuspended in DPBS −/−, aliquoted into 200 μL tubes, and stored at −80 °C until use. EV size and quantity was confirmed using a nano-particle analyzer (NanoSight 300; Malvern Panalytical; Cat. NS300). A liter of conditioned media can produce ~4.18 × 10^12^ EV/mL with a size distribution of 110 ± 59.14 nm (diameter). The EVs were visualized, and size was re-confirmed via transmission electron microscopy (TEM; JEOL TLD.; Jem-1400 Plus). Timo negative staining was performed before TEM imaging, using 2% UA in water and Grid 400 mesh Formvar carbon (conducted by the UCSD, Cellular and Molecular Medicine Electron Microscopy Core; RRID:SCR_022039). To confirm that our isolated EVs were exosomes, a protein analysis was done to confirm the presence of commonly known EV biomarkers using ProcartaPlex^™^ Human Exosome Characterization Panel 6-Plex (ThermoFisher; Cat. EPX060–15845-901). The EVs were resuspended in DPBS −/− to achieve a concentration of 2.25 × 10^8^ EVs per 50 μL. On the day of EV delivery, mice were anesthetized via isoflurane (as above) and received 50 μL of either sham (sterile DPBS −/−) or EV injections via the RO route. The mice received a total of four RO injections, once per week over the course of four weeks.

### 2.7. Cognitive function tasks

#### Object Recognition and Object Location Memory.

The object recognition memory (ORM) and object location memory (OLM) tasks rely on the intact functioning of the hippocampus, perirhinal cortex, and medial prefrontal cortex. ORM assesses episodic memory by measuring the animal's preference for exploring a novel object, while OLM evaluates spatial recognition memory [35]. Both tasks were conducted in testing rooms with appropriate lighting (50–70 Lux) using four square arena boxes, digital camera recording equipment (Noldus), and tracking software (EthoVision XT v17; RRID:SCR_000441). For ORM, mice were habituated in the open-field arenas (30 × 30 × 30 cm) containing a thin layer of bedding but no objects for 10 min over three consecutive days. Open Field Test (OFT) data was derived from this habituation period. During the familiarization phase, mice explored two identical plastic objects secured 16 cm apart in opposing corners for 5 min. After a 5-min break in their home cages, one familiar object was replaced with a novel object (both objects were cleaned with 10% ethanol and dried). Mice were then returned to the arena for a 5-min test phase, after which they were returned to their home cages. The OLM task was performed one week later. Mice were familiarized with the testing room and arena boxes (30 × 30 × 30 cm) containing blue tape along one of the walls and bedding. During familiarization, mice were allowed to explore two identical objects placed 16 cm apart for 5 min. After a 5-min break in their home cages and cleaning of objects with 10% ethanol, one object was relocated to a novel position 16 cm from the opposite corner, while the other object remained stationary. Mice were then returned to the arena for a 5-min test phase. Behavior across all phases was recorded, and the “head direction to zone” feature of EthoVision XT software was used to track exploration time. To ensure unbiased analysis, time spent interacting (nose within 2 cm) with the familiar and novel/relocated objects was scored by observers blinded to experimental conditions. The preference index (PI) was calculated as:

$$PI=\left(\frac{\mathrm{Novel}\;\mathrm{exploration}\;\mathrm{time}}{\mathrm{Total}\;\mathrm{exploration}\;\mathrm{time}}\right)-\left(\frac{\mathrm{Familiar}\;\mathrm{exploration}\;\mathrm{time}}{\mathrm{Total}\;\mathrm{exploration}\;\mathrm{time}}\right)\times 100$$

### 2.8. Fear extinction memory consolidation

To assess the prefrontal cortex, hippocampal-cortical-amygdala circuit-dependent fear conditioning and extinction processes, fear extinction (FE) memory consolidation testing was conducted [36]. Testing took place in a behavioral conditioning chamber (17.5 × 17.5 × 18 cm; Coulbourn Instruments) with steel shock floors (3.2 mm diameter slats, 8 mm spacing) and a waste tray treated with 10% vinegar. On Day 1 (fear conditioning), mice were habituated to the chamber for 2 min, followed by three pairings of an auditory conditioned stimulus (CS; 16 kHz tone, 80 dB, 120 s) co-terminating with a foot shock unconditioned stimulus (US; 0.6 mA, 1 s) at 2-min intervals. Extinction training occurred on Days 2–4, during which mice were habituated to the chamber for 2 min before being exposed to 20 non-reinforced CS tones (16 kHz, 80 dB, 120 s) at 5-s intervals. On Day 5, a final extinction test was conducted by presenting mice with three non-reinforced CS tones at 2-min intervals. Freezing behavior was recorded via an overhead camera and analyzed using FreezeFrame software (Coulbourn Instruments). The software calculated a motion index for each video frame, with immobility thresholds set individually by a blinded observer. Freezing was defined as continuous immobility lasting 1 s or longer. For the extinction test, the percentage of time spent freezing was calculated as an indicator of fear memory retention.

### 2.9. Elevated plus maze

The elevated plus maze (EPM) task evaluates anxiety-like behavior by comparing the time spent in open and closed arms of a plus-shaped platform elevated above the ground. The maze consists of one open arm without walls or a roof and one closed arm with high walls and a darker environment. Before testing, the maze was cleaned with an odor-eliminating disinfectant (Virkon; Lanxess; Cat. RSL_31/Virks) and dried. Each mouse was placed at the center of the maze and allowed to explore for 5 min. Behavior was recorded using the same camera hardware and analyzed with Noldus tracking software as previously described. The proportion of time spent in the open versus closed arms was used as a measure of anxiety-like behavior, with reduced time in the open arm indicating higher anxiety.

### 2.10. Tissue collection

Anesthetized mice (inhaled isoflurane) were humanely euthanized using an intracardiac perfusion technique using ice-cold saline (0.9% saline with heparin, 10 U/mL). Mice intended for molecular assays had their brains extracted, the prefrontal cortex (PFC) and hippocampus excised, and flash frozen in liquid nitrogen. Flash frozen brains were stored at −80 °C until usage. All other brains were used for immunohistochemistry (IHC) analyses and were fixed by 4% paraformaldehyde (PFA) made in DPBS (pH 7.4; Sigma-Aldrich; Cat. 158127). Brains were extracted and soaked in 4% PFA overnight, then switched to storage in DPBS containing 0.05% Sodium Azide (pH 7.4; Sigma-Aldrich; Cat. S2002). For cryosectioning, brains were prepared in a sucrose gradient (10% to 30% w/v; pH 7.4; Sigma; Cat. S7903) and embedded in the O.C. T compound (VWR; Cat. 25608903), and then coronally cryosectioned using a cryostat (Cryostat HM525 NX; Cat. 95-664-0 EC). The free-floating sections (30 μm) were stored in 24-well plates in PBS with 0.05% Sodium Azide for the staining procedures.

### 2.11. Immunofluorescence staining

Free floating brain sections were collected from each group (2 sections with a visible hippocampal region per brain; four brains per group) for dual/triple immunofluorescence staining. For synaptic vesicle glycoprotein 2A (SV2a), post-synaptic density protein 95 (PSD-95), and glial fibrillary acidic protein (GFAP) staining, tissues were washed in 1X TBS (dilute with MilliQ-Water from 10X TBS; pH 7.4; Bioland Scientific; Cat. TBS01–03) three times (5 min each) and Tris-A solution (0.1% Triton-X; FisherSci; Cat. 85111; in 1X TBS, ph 7.4) at room-temp for 10 min. Sections for the three stains were incubated in blocking solution with each respective host, 4% bovine serum albumin (BSA) in Tris-A buffer for PSD-95 marker; 10% natural goat serum (NGS) in Tris-A buffer for SV2a and GFAP markers (NGS; Jackson ImmunoResearch; Cat. 005-000-121, and BSA; Sigma-Aldrich; Cat. 05470) at room-temp for 45 min. Sections were incubated in primary antibodies at 4°C overnight [1:1000 for Mouse anti SV2a, (DHSB; Cat. SV2) in 3% NGS in TBS buffer for the SV2a marker, and 1:500 for Mouse anti PSD-95, (Fisher Scientific; Cat. MA1–045), with 2% BSA in TBS buffer for PSD-95 marker, and 1:500, Mouse anti GFAP, (Millipore, Cat. AB5804) with 3% NGS in TBS for the GFAP marker]. Sections were placed at room temperature for 1 h the next day before staining for secondary antibodies. Sections were washed with 1X TBS (3 times, 5 min each) and then transferred to secondary antibodies to incubate at room-temp for 1 h to visualize the target antigens [1:1000 Goat anti Mouse AF 568, (Abcam; Cat. ab175473) in 3% NGS in TBS buffer for the SV2a marker, and 1:1000 Goat anti Mouse AF 647, (Abcam; Cat. ab150115) for PSD-95 marker in 2% BSA in TBS, and 1:500 Goat anti Mouse AF 568, (Abcam Cat. ab175473) in 3% NGS in TBS buffer for GFAP marker]. Lastly, brain sections were counterstained with DAPI nuclear dye (1 μmol/L; FisherSci; Cat. D1306) in 1X TBS at room-temp for 15 min. Stained sections were washed with 1X TBS (2 times, 5 min each) and mounted on superfrost slides (FisherSci; Cat. 22-037-246) using VectaShield antifade mounting medium (VectaShield; Cat. H-1000–10).

IHC for Cluster of Differentiation 68 and Ionized calcium-binding adaptor molecule 1 (CD68-IBA1) markers followed similar steps above with some minor changes. First, brain sections were washed with PBS-0.3% Tween 20 (Sigma-Aldrich; Cat. 9005-64-5) three times (5 min each) and then incubated in 3% peroxide solution (Dilute 30% Hydrogen Peroxide with 1% Methanol; (FisherSci; Cat. A412500) in 1X DPBS at room-temp for 30 min to block any endogenous peroxidase activity. Sections were washed in 1X DPBS (3 times, 5 min each) prior to incubating in blocking solution (4% BSA in DPBS-03% Tween-20) at room-temp for 30 min. Brain sections were then incubated in primary antibodies at 4°C overnight [1:500, Rat anti Mouse CD68 (Bio-rad; Cat. MCA1957) for CD68 marker only, combined with 1:500 Rabbit anti IBA1 (FUJIFILM Wako; Cat. 019-1974), for CD68/IBA1 markers, with 1% BSA in PBS-0.3% Tween-20 buffer]. Sections were washed and stained in secondary antibodies at room-temp for 1 h [1:1000; Goat anti Rat AF 647 (Abcam; Cat. ab150159) for CD68 marker, and 1:500; Goat anti Rabbit AF 488 (FisherSci; Cat. a11008) for IBA1 marker]. Sections were washed and counterstained with DAPI nuclear dye (1 μmol/L; FisherSci; Cat. D1306) and mounted on superfrost slides (FisherSci; Cat. 22-037-246) using VectaShield antifade mounting medium (VectaShield; Cat. H-1000–10).

### 2.12. Hematoxylin and eosin staining

To determine the impact of cancer, RT-TMZ and systemic hNSC-EV treatment on the gross pathology of peripheral organs, we collected lungs, liver and kidneys from a small cohort of animals. Tissues were fixed overnight using FineFIX fixative (Cat. No. 70147G; Milestone Medical), post-fixed using paraffin and sectioned at a thickness of 4 μm. Routine Hematoxylin and Eosin (H&E) staining was performed by the UC Irvine Experimental Tissue Resource Shared facility.

### 2.13. Microscopy and 3D algorithm-based volumetric quantification

Immunostained brain sections were captured at high resolution (1024 *xy* pixels, for CD68-Iba-1, and 2048p for SV2a, PSD-95, and GFAP markers) using a laser-scanning confocal microscope (Nikon Eclipse Ti C2) with a 20 × and 40 × oil-immersion objective lenses, acquiring 0.5 μm thick z-stacks. The resulting high-resolution images were deconvoluted, and 3D volume surfaces for each antigen of interest were generated using Imaris (Oxford Instruments; RRID: SCR_007370), an advanced image analysis software. Synapse-related markers such as SV2a and PSD-95 were quantified by analyzing the surface volumes of synaptic puncta. For the microglia activation marker, CD68-IBA1, volumetric co-localization between the surfaces of the two markers was determined and analyzed. For the astrocytosis marker (GFAP), total volume of astrocytes was analyzed and quantified. All image analyses were performed using automated batch processing, ensuring consistent application of parameters across all images for unbiased results.

### 2.14. Differential gene expression analysis

Flash frozen micro-dissected brains intended for molecular analyses were ground to a fine powder with an ice-cold mortar and pestle. RNA was isolated using the RNeasy^®^ Plus Universal Mini Kit (Qiagen; Cat. 73404) following manufacturers protocol. RNA concentration and integrity was confirmed on the Nano-drop (ThermoFisher; Cat. ND-ONE-W). Gene expression was analyzed using the commercially available nCounter^®^ Mouse Neuroinflammation panel (Bruker; Cat. 531-115000237) to evaluate gene expression related to neuroinflammation in the treatment groups. The panel includes 757 genes involved in neuroimmune interactions, and 13 internal reference genes, or ‘housekeeping genes’. Data collection was completed by the Genomics Research and Technology Hub at UC Irvine (RRID: SCR_026615). The raw value data were normalized on the nCounter^®^ Software utilizing a two-step normalization process: an initial Positive Control Normalization, followed by a CodeSet Content Normalization using the 13 internalized housekeeping genes. Differential expression analysis was performed on log_2_-transformed expression values using an unpaired two-sample Welch's *t*-test (two-sided). For each experimental condition (RT-TMZ + Shef6 EV, RT-TMZ + UCI-191 EV, CT2A + RT-TMZ + Shef6 EV, and CT2A + RT-TMZ + UCI-191 EV), gene expression levels were compared against their respective control groups: RT-TMZ + Vehicle and CT2A + RT-TMZ + Vehicle Transcriptional changes in this dataset were analyzed using thresholds of **|log2FC| > 1.0** and **|log2FC| > 0.2** to capture both robust and subtle, potentially meaningful differences in gene expression. This approach ensured that gene expression patterns were evaluated not only under a stringent cutoff for high-confidence differentially expressed genes, but also under a more exploratory threshold to visualize modest transcriptional changes. P values were adjusted for multiple testing using the Benjamini–Hochberg false discovery rate (FDR) method, with an FDR cutoff of 0.5 applied for this exploratory analysis.

## 3. Results

### 3.1. Characterization of extracellular vesicles

**Fig. 1.**
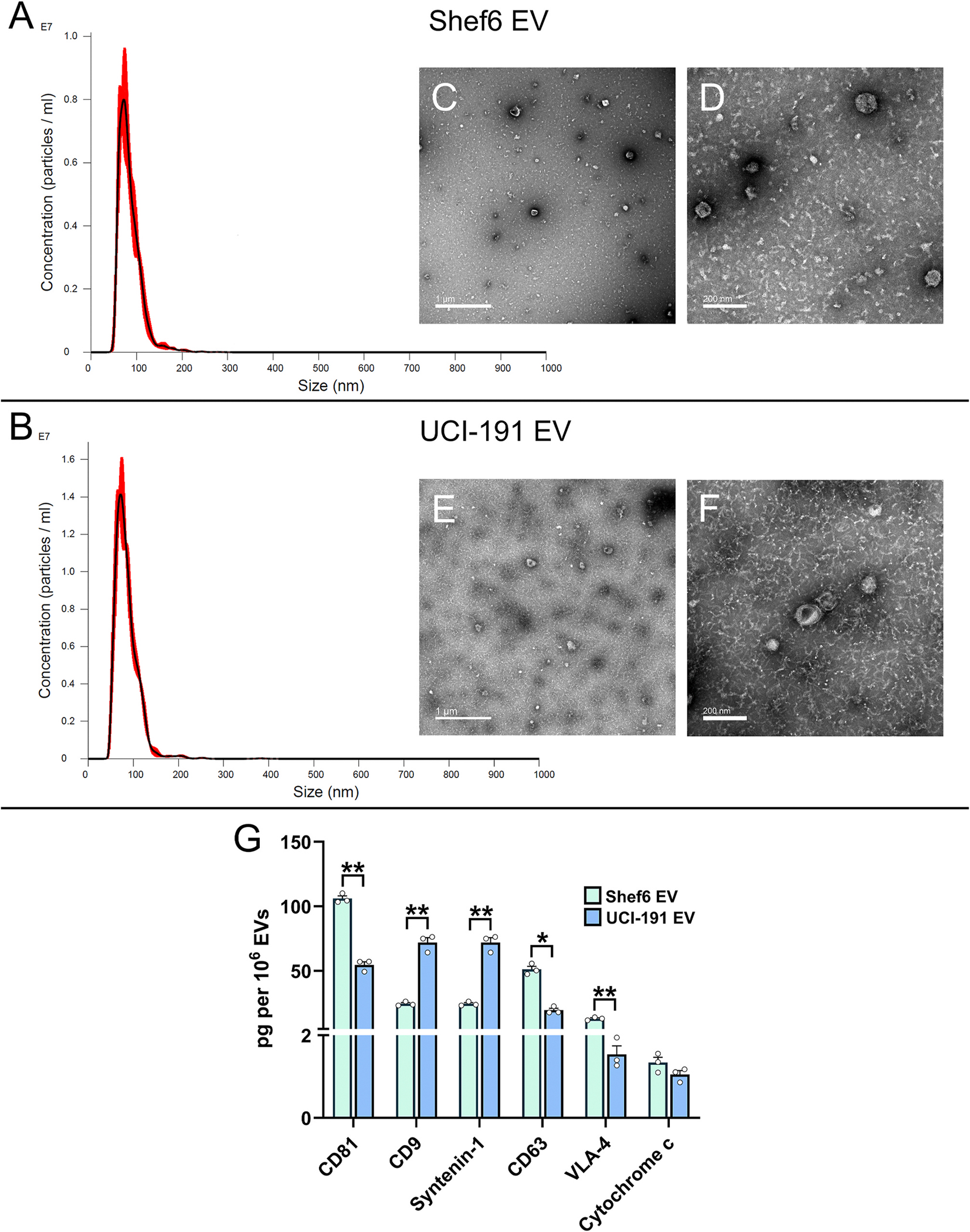
hNSC-EV characterization by size and protein markers. (**A**-**G**) Nano-vesicles isolated from hNSC conditioned media were characterized for size and protein marker content. (**A**-**B**) Particle size distribution graph for EV samples (in triplicate) characterized using a laser nanoparticle analyzer (NanoSight 3000). (**C**-**F**) Representative transmission electron micrographs of Shef6-derived (**C**-**D**) and UCI-191-derived (**E**-**F**) EVs are shown. (**G**) EVs (in triplicate) were isolated, lysed, and protein content was confirmed using the ProcartaPlex^™^ Human Exosome Characterization Panel. TEM magnifications were 10,000 × (**B** and **E**) and 40,000 × (**C** and **F**). Scale bars, 1 μm (**B** and **E**) and 200 nm (**C** and **F**). *P*-values were derived from multiple unpaired *t* tests. *, P ≤ 0.05; **, P ≤ 0.01; ***P ≤ 0.001; ****, P ≤ 0.0001.

Exosome size was confirmed and quantified using a laser nanoparticle analyzer. EVs from both Shef6 and UCI-191 hNSCs had an average size of approximately 80 nm (Fig. 1A and B), consistent with prior exosome literature [37]. EV morphology was characterized using TEM (Fig. 1C-D and E-F). EVs were characterized biochemically for EV-specific protein markers. EV proteins were purified and characterized as described in Methods and Materials. EVs derived from both hNSCs expressed CD81, CD9, CD63, syntenin-1, Very Late Antigen-4 (VLA-4), and cytochrome *c*. The expression of CD81, CD63, and VLA-4 were significantly higher in Shef6 EVs compared to UCI-191 EVs (P ≤ 0.01 for CD81 and VLA-4; P ≤ 0.05 for CD63). On the other hand, CD9 and syntenin-1 expression were significantly higher in UCI-191 EVs (Fig. 1G; P ≤ 0.01).

### 3.2. hNSC-EVs alleviate cognitive dysfunction following combined fractionated cranial irradiation and chemotherapy

**Fig. 2.**
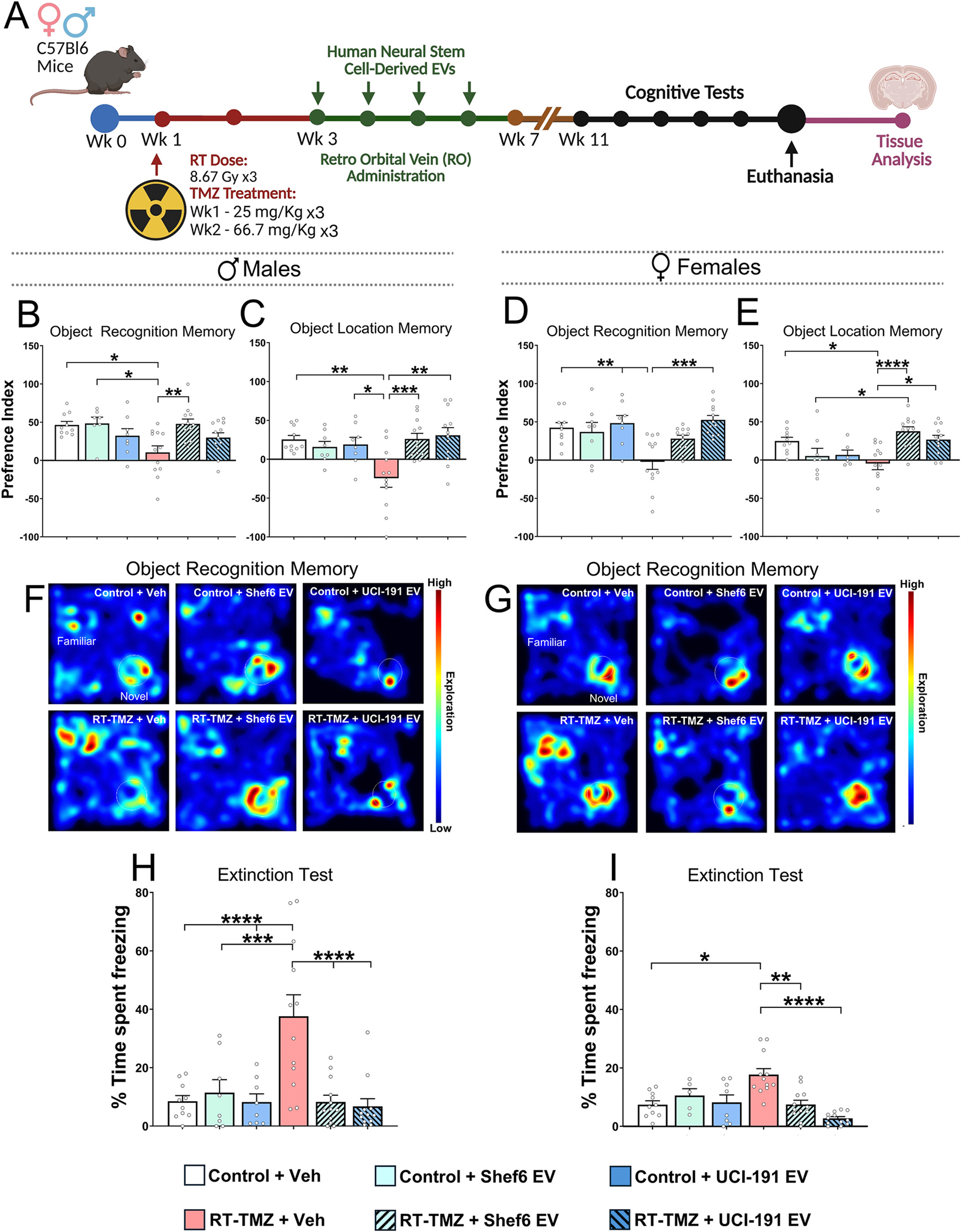
hNSC-EVs prevent cognitive impairments following combined cranial radiation therapy and chemotherapy. (**A**) Research design: Three-month-old WT (C57BL6) male and female mice received fractionated cranial RT (8.67 Gy × 3, every other day) and concomitant TMZ (25 mg/kg × 3, IP) 24h after each RT, and adjuvant TMZ (66.7 mg/kg × 3, IP) 72h after the concomitant TMZ, every other day, over two weeks. 72 h after completion of RT-TMZ treatment, mice received RO injections of hNSC-EVs (RT-TMZ + Shef6 EV; RT-TMZ + UCI-191 EV) or sham RO injections (RT-TMZ + Veh; sterile PBS) once weekly for 4-weeks. Four weeks after the final EV treatment, cognitive and behavioral tasks (ORM, OLM, FE, EPM, OFT) were administered. After completion of cognitive testing, animals were euthanized, and their brains were collected for cellular and molecular analyses (See Suppl. Fig. S1 for OFT and EPM data; **A**, designed using *BioRender*). (**B**-**E**) Spontaneous exploration tasks, ORM and OLM, for male and female mice. Cognitive performance data are reported as Preference Index (PI), calculated by [(*T*_*Novel*_/*T*_*Total*_)-(*T*_*Familiar*_/*T*_*Total*_)] × 100, where *T* is the time (s) exploring novel or familiar object (or location), and the total time exploring both. (**F**-**G**) Representative heatmaps depicting animal exploration of familiar or novel objects throughout the ORM arena (See Suppl. Fig. S2 for OLM heatmaps). (**H**-**I**) Analysis of the percentage of time spent freezing on the final Test Day of FE in male (**H**) and female (**I**) mice. Initial fear conditioning (*T*_*1–3*_) and extinction training (*Days 1–3*) are graphically depicted in Suppl. Fig. S3. Data are represented by mean ± SEM (N = 5–12 animals/group). *P*-values were calculated by two-way ANOVA and Bonferroni's multiple comparisons test. *, P ≤ 0.05; **, P ≤ 0.01; ***P ≤ 0.001; ****, P ≤ 0.0001.

12-week-old male and female wild-type (WT; C57BL/6) mice were exposed to fractionated RT and TMZ as described in Methods and Fig. 2A. 72h after the final chemotherapy dose, mice received a systemic intravenous (RO vein) injection of either Shef6 or UCI-191 hNSC-EVs, once weekly for four weeks. Four weeks after the completion of EV treatment, all groups (Control + Veh; Control + Shef6 EV; Control + UCI-191 EV; RT-TMZ + Veh; RT-TMZ + Shef6 EV; RT-TMZ + UCI-191 EV) were habituated and tested on behavioral and cognitive function tasks.

For the EPM test in male mice, RT-TMZ + Veh spent significantly lower percentage of time exploring the open arm of the maze when compared to Control + Veh and Control + Shef6 EV (Supp. Fig. 1A; P ≤ 0.001 and P ≤ 0.05, respectively). RT-TMZ + Shef6 EV demonstrated an increase in open arm exploration compared to RT-TMZ + Veh (Supp. Fig. 1A; P ≤ 0.01). On the other hand, Control + UCI-191 EV and RT-TMZ + UCI-191 EV groups showed reduced exploration in the open arm compared to Control + Veh (Supp. Fig. 1A; P ≤ 0.01 and P ≤ 0.05, respectively). In female mice, RT-TMZ + Veh, Control + UCI-191 EV, and RT-TMZ + UCI-191 EV displayed a significant decrease in exploration of the open arms compared to Control + Veh (Supp. Fig. 1B; P ≤ 0.0001, P ≤ 0.05, and P ≤ 0.001, respectively). RT-TMZ + Shef6 EV showed an increase in exploration when compared to RT-TMZ + Veh (Supp. Fig. 1B; P ≤ 0.001).

For the OFT in male mice, RT-TMZ + Veh showed a decline in exploration of the central zone of the arena (30% total arena area) when compared to Control + Veh, Control + Shef6 EV, and Control + UCI-191 EV (Supp. Fig. 1C; P ≤ 0.05). RT-TMZ + UCI-191 EV had increased exploration compared with RT-TMZ + Veh (Supp. Fig. 1C; P ≤ 0.01). Similarly, the female RT-TMZ + Veh group showed a decrease in exploration of the central zone compared to the Control + Veh and Control + Shef6 EV (Supp. Fig. 1D; P ≤ 0.01). RT-TMZ + Shef6 EV demonstrated an increase in the central zone exploration compared to RT-TMZ + Veh (Supp. Fig. 1D; P ≤ 0.01). The decrease in exploration of RT-TMZ + Veh during EPM and OFT tasks compared to Control + Veh indicates radiation and chemotherapy-induced anxiety-like behavior. RT-TMZ-treated mice that received either hNSC-EV treatment did not display anxiety-like behavior and were willing to explore.

Evaluation of male mice performance on the ORM task in the RT-TMZ + Veh group showed a significantly lower Preference Index (PI) compared to the Control + Veh and Control + Shef6 EV groups (Fig. 2B; P ≤ 0.05). Shef6 EV treatment in RT-TMZ-exposed mice had an increased PI compared with the RT-TMZ + Veh group (Fig. 2B; P ≤ 0.01); RT-TMZ + UCI-191 EV showed a trend toward improved PI. For females, RT-TMZ + Veh had a lower PI compared to Control + Veh and Control + UCI-191 EV (Fig. 2D; P ≤ 0.01). The RT-TMZ + UCI-191 EV group had a higher PI compared to RT-TMZ + Veh (Fig. 2D; P ≤ 0.001). These data indicate that RT-TMZ treatment disrupts object recognition memory formation, and hNSC-EV treatment is neuroprotective. The ORM behavior data is reflected in the representative exploration heatmaps generated for treatment groups (Fig. 2F and G).

Analysis of OLM testing in male mice revealed a decrease in PI in RT-TMZ + Veh compared to Control + Veh and Control + UCI-191 EV (Fig. 2C; P ≤ 0.01 and 0.05, respectively). Both hNSC-EV-treated groups displayed an increase in PI (Fig. 2C; RT-TMZ + Shef6 EV, P ≤ 0.001; RT-TMZ + UCI-191 EV, P ≤ 0.01). Similarly, the RT-TMZ + Veh female group had a lower PI than the Control + Veh counterpart (Fig. 2E; P ≤ 0.05). As with the males, both hNSC-EV treated female groups had an increase in PI compared to RT-TMZ + Veh (Fig. 2E; RT-TMZ + Shef6 EV, P ≤ 0.0001; RT-TMZ + UCI-191 EV, P ≤ 0.05). These data indicate that RT-TMZ-treated mice exhibit impaired spatial memory, whereas mice treated with hNSC-EVs do not. This is reflected in the representative heatmaps for OLM in both sexes (see Supp. Fig. S2A–B).

Animals were then administered hippocampal-cortical-amygdala circuit-dependent learning using fear-extinction training and a subsequent fear-extinction test in males (Fig. 2H) and females (Fig. 2I). All mice successfully acquired the conditioned fear response, evidenced by the increase in percentage of freezing during the *Conditioning phase* (*T1-T3*; Supp. Fig. S3A–B). Extinction testing of male mice showed that RT-TMZ + Veh were unable to extinguish the conditioned fear response and showed a high percentage of freezing compared to Control + Veh, Control + Shef6 EV, and Control + UCI-191 EV (Fig. 2H; P ≤ 0.0001, 0.001, and 0.0001, respectively). Both RT-TMZ + Shef6 EV and RT-TMZ + UCI-191 EV showed a significantly lower percentage of time freezing compared to RT-TMZ + Veh (Fig. 2H; P ≤ 0.0001). In female mice, RT-TMZ + Veh had a higher percentage of freezing during extinction testing compared to Control + Veh (Fig. 2I; P ≤ 0.05) but was lower in RT-TMZ groups receiving either Shef6 or UCI-191 EVs (Fig. 2I; P ≤ 0.01 and 0.0001, respectively). These data indicate that RT and TMZ impair animals’ ability to extinguish a conditioned fear response, whereas hNSC-EVs protect that cognitive ability.

### 3.3. hNSC-EVs prevent neuroinflammation following combined cranial irradiation and chemotherapy

**Fig. 3.**
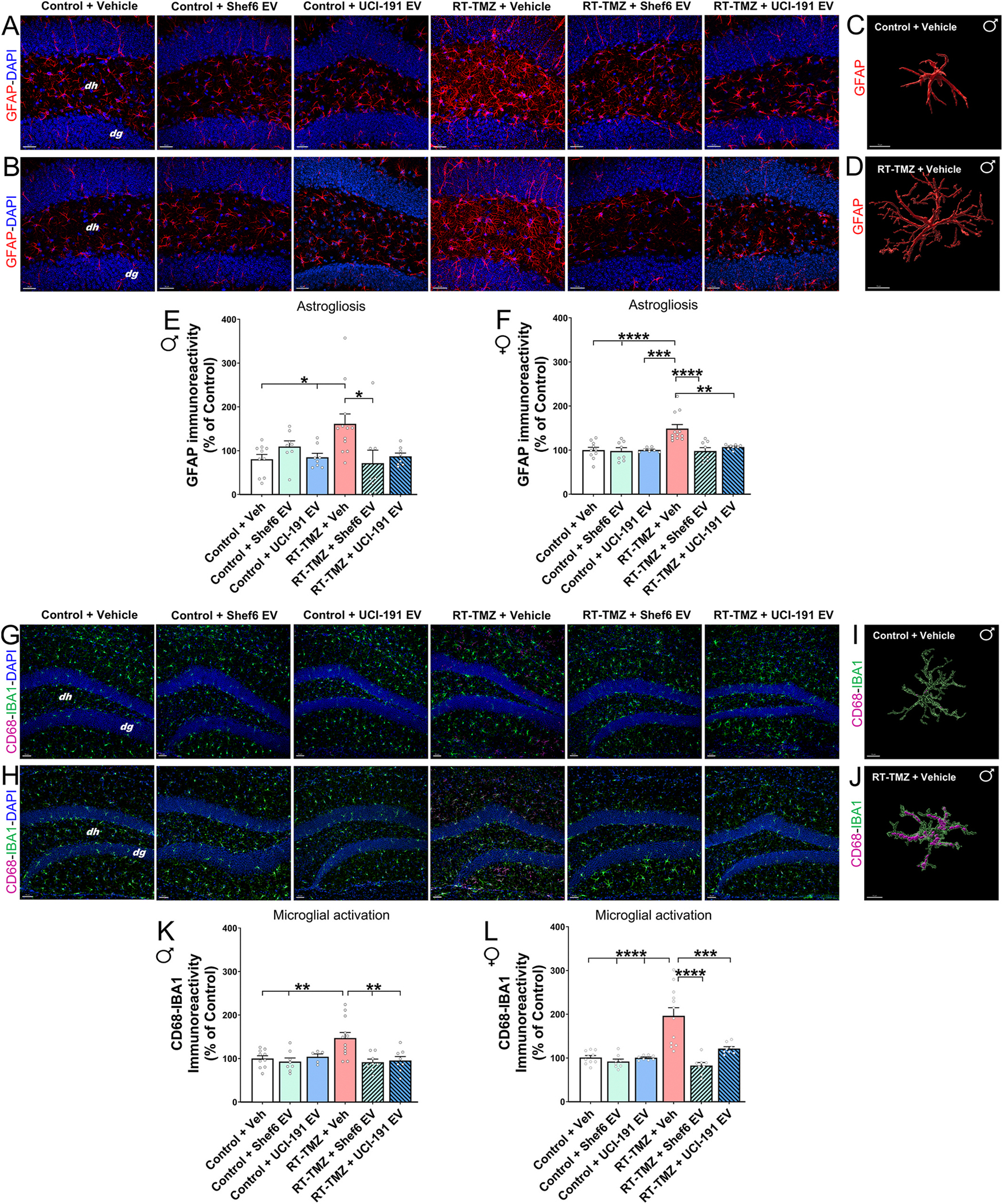
hNSC-EVs prevent neuroinflammation following combined cranial radiation and chemotherapy. (**A**-**B**) Confocal fluorescent micrographs of astrogliosis in male (**A**) and female (**B**) mice. Immunofluorescence staining and confocal microscopy of the astrocytic cytoskeleton glial fibrillary acidic protein (GFAP; red) with DAPI nuclear counter stain (blue) in the hippocampal dentate hilus (*dh*) and dentate gyrus (*dg*). (**C**-**D**) High resolution immunoreactive surface rendering of single cells in the Control + Veh (**C**) and RT-TMZ + Veh (**D**) groups in male brains for GFAP^+^ astrocytes (See Suppl. Fig. S4 for female single cell surface renderings). (**E**-**F**) Volumetric analysis of the 3D algorithm-based quantification of GFAP in male (**E**) and female (**F**) mice. (**G**-**H**) Dual labeled confocal fluorescent micrographs of IBA1 and CD68 colocalization to quantify microglial activation in male (**G**) and female (**H**) mice. Immunofluorescence staining and confocal microscopy for a pan microglial marker (IBA1, green), and lysosomal membrane marker CD68 (magenta) with DAPI nuclear counter stain (blue) in the hippocampal dentate hilus (*dh*) and dentate gyrus (*dg*). (**I**-**J**) High resolution immunoreactive surface rendering of single cell images in the Control + Veh (**I**) and RT-TMZ + Veh (**J**) groups in male brains for IBA1-CD68^+^ dual-labeled microglia (See Suppl. Fig. S4 for female single cell surface renderings). (**K**-**L**) 3D algorithm-based volumetric quantification of CD68-IBA1 in male (**K**) and female (**L**) mice. *dh, dentate hilus,* and *dg, dentate gyrus*. Confocal z stacks were acquired at 40 × (**A** and **B**, 1.30 numerical aperture) and 20 × (**G** and **H**, 0.75 numerical aperture) magnifications. Scale bars, 30 μm (**A** and **B**), 50 μm (**G** and **H**), and 10 μm (**C** and **D**; **I** and **J**). Data are represented by mean ± SEM (N = 5–12 animals/group). *P* values were calculated by one-way ANOVA and Tukey's multiple comparisons tests. *, P ≤ 0.05; **, P ≤ 0.01; ***P ≤ 0.001; ****, P ≤ 0.0001. (For interpretation of the references to color in this figure legend, the reader is referred to the Web version of this article.)

To determine the effects of RT, TMZ, and hNSC-EVs on neuroinflammation, we performed dual immunofluorescence and 3D algorithm-based volumetric quantification of immunoreactive surfaces for two cellular hallmarks of neuroinflammation: astrogliosis, via GFAP (Fig. 3A–F), and microglial activation via CD68-IBA1 (Fig. 3G–L). IBA1 is a pan microglial marker and CD68 is a lysosomal membrane protein. Analysis of confocal z stacks and quantification of GFAP^+^ surface renderings showed significantly elevated GFAP immunoreactivity and cellular morphology (Fig. 3A–D) in the RT-TMZ + Veh male mice compared to Control + Veh and Control + UCI-191 EV (P ≤ 0.05). While RT-TMZ mice treated with Shef6 EVs had significantly lower GFAP expression (Fig. 3E; P ≤ 0.05), and a trend of lower expression was observed in the RT-TMZ + UCI-191 EV group. RT-TMZ exposure also elevated GFAP immunoreactivity in the female mice compared to Control + Veh, Control + Shef6 EV, and Control + UCI-191 (Fig. 3F; P ≤ 0.0001, 0.0001, and 0.001, respectively). Both hNSC-EV-treated female groups had lower GFAP expression compared to RT-TMZ + Veh (Fig. 3F; RT-TMZ + Shef6 EV, P ≤ 0.0001; RT-TMZ + UCI-191 EV, P ≤ 0.01). Quantification of microglial activation in male mice found elevated CD68-IBA1 co-localization (Fig. 3G–J) in the RT-TMZ + Veh mice compared to Control + Veh and Control + Shef6 EV (Fig. 3K; P ≤ 0.01). Treatment with both EVs (RT-TMZ + Shef6 EV and RT-TMZ + UCI-191 EV) reduced microglial activation compared to RT-TMZ + Veh (Fig. 3K; P ≤ 0.01). A similar extent of RT-TMZ treatment-induced microglial activation was observed in female mice (RT-TMZ + Veh) compared to Control + Veh, Control + Shef6 EV, and Control + UCI-191 EV (Fig. 3L; P ≤ 0.0001). Both types of EVs reduced microglial activation compared to RT-TMZ + Veh (Fig. 3L; RT-TMZ + Shef6 EV, P ≤ 0.0001; RT-TMZ + UCI-191, P ≤ 0.001). A representative surface rendering of astrocytes from the RT-TMZ + Veh groups showed activated morphology characterized by cellular hypertrophy, and thicker and longer stelae of GFAP^+^ branches compared to Control + Veh (Fig. 3C and D) [38]. Concurrently, microglia from the Control + Veh group displayed the classic morphology of homeostatic microglia; small soma with many ramified processes, whereas microglia from the RT-TMZ + Veh group had elevated expression of the phagocytic marker CD68 (Fig. 3I and J) [39]. Representative surface renderings of astrocytes and microglia from female mice in the Control + Veh and RT-TMZ + Veh groups is shown in Supp. Fig. S4A–B. These data indicate that RT and TMZ treatments elevated astrogliosis and microglial activation, suggesting neuroinflammation, while hNSC-EVs reduced gliosis *in vivo*.

### 3.4. hNSC-derived EVs prevent cranial radiation and chemotherapy-induced synaptic density loss

To evaluate synaptic density following RT, TMZ, and hNSC-EVs, we quantified two synaptic markers: SV2a and PSD-95. We analyzed synaptic protein expression in two brain regions important for learning and memory: the pyramidal cell layer (*pyr*) and corresponding stratum radiatum (*sr*) of the hippocampal CA1, and the molecular layer (*ml*) of the dentate gyrus (*dg*).

**Fig. 4.**
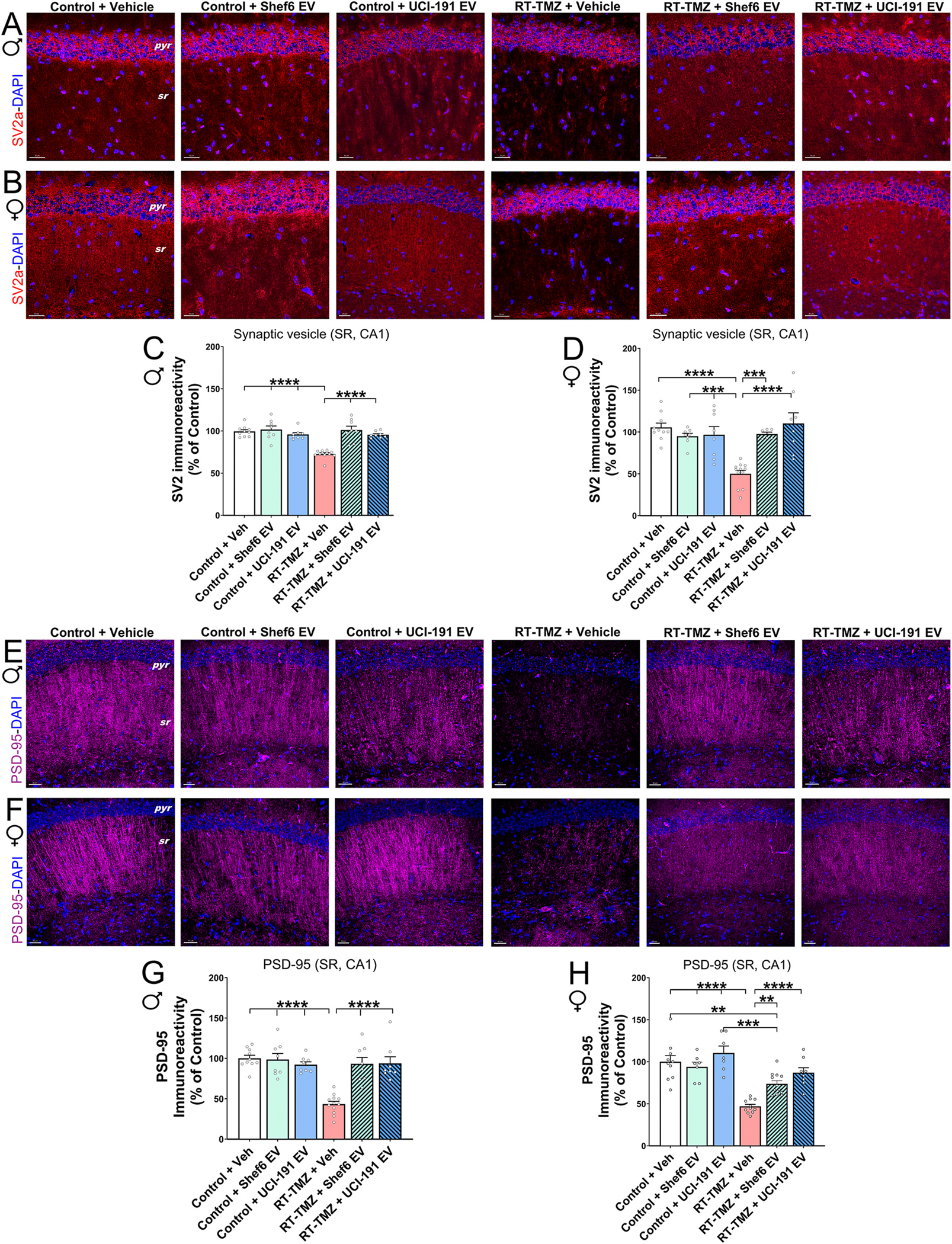
hNSC-EVs prevent synaptic density loss following combined cranial radiation therapy and chemotherapy.Fluorescent confocal micrographs and volumetric quantification of synaptic density in male (**A**, **C**, **E**, **G**) and female (**B**, **D**, **F**, **H**) mice. (**A**-**D**) Immunofluorescence staining, confocal microscopy, and 3D algorithm-based quantification for synaptic vesicle glycoprotein 2A (SV2a; red) with DAPI nuclear counter stain (blue) in the hippocampal CA1 pyramidal cell layer (*pyr*) and stratum radiatum (*sr*). (See Suppl. Fig. S5 for quantification in the dentate molecular layer). (**E**-**H**) Representative confocal micrographs and volumetric analysis of postsynaptic density protein, PSD-95 (magenta), with DAPI nuclear counter staining in the hippocampal *pyr* and *sr* subregions of CA1. (See Suppl. Fig. S6 for quantification in the dentate molecular layer). Data are represented by mean ± SEM (N = 6–12 animals/group). *P* values were calculated by one-way ANOVA and Tukey's multiple comparisons tests. *, P ≤ 0.05; **, P ≤ 0.01; ***P ≤ 0.001; ****, P ≤ 0.0001. Scale bars, 30 μm. (For interpretation of the references to color in this figure legend, the reader is referred to the Web version of this article.)

Representative images of SV2a expression in the CA1 (*pyr*, *sr*) for all groups in male and female mice indicate somatic and dendritic expression of SV2a (Fig. 4A and B). Representative images of SV2a expression in the dentate molecular layer (*dg*, *ml*) is shown in Supp. Fig. S5A–B. SV2a expression in the CA1 (*pyr*, *sr*) of male mice was significantly reduced following RT and TMZ treatments (RT-TMZ + Veh) compared to Control + Veh, Control + Shef6 EV, and Control + UCI-191 EV (Fig. 4C; P ≤ 0.0001). Both hNSC-EV treatment groups had elevated SV2a expression compared to RT-TMZ + Veh (Fig. 4C; P ≤ 0.0001). RT-TMZ + Veh male mice also had lower SV2a immunoreactivity in the dentate molecular layer (*dg*, *ml*) compared to Control + Veh and Control + UCI-191 EV (Supp. Fig. 5C; P ≤ 0.0001). RT-TMZ + UCI-191 EV improved SV2a expression compared to RT-TMZ + Veh, and there was a trend of increased expression in RT-TMZ + Shef6 EV group (Supp. Fig. 5C; P ≤ 0.0001). Control + Veh males had higher levels of SV2a compared to non-irradiated and irradiated Shef6 EV treated mice (Supp. Fig. 5C; P ≤ 0.05 and 0.001, respectively). Control + UCI-191 EV also had higher SV2a expression compared to RT-TMZ + Shef6 EV (Supp. Fig. 5C; P ≤ 0.05). Interestingly, SV2a immunoreactivity was significantly higher in the RT-TMZ + UCI-191 EV compared to RT-TMZ + Shef6 EV and (Supp. Fig. 5C; P ≤ 0.05). We found concurrent observations in the SV2a expression in female CA1 in RT-TMZ + Veh groups showing a reduced expression compared to Control + Veh, Control + Shef6 EV, and Control + UCI-191 EV (Fig. 4D; P ≤ 0.0001, 0.001, and 0.001, respectively). RT-TMZ + Veh mice also had lower levels of SV2a compared to RT-TMZ + Shef6 EV and RT-TMZ + UCI-191 EV (Fig. 4D; P ≤ 0.001 and 0.0001, respectively). Quantification of SV2a in the dentate molecular layer showed a significantly lower expression in the RT-TMZ + Veh compared to Control + Veh and Control + Shef6 EV groups (Supp. Fig. 5D; P ≤ 0.01 and 0.001, respectively). Both hNSC-EV treated groups showed increased SV2a expression compared to RT-TMZ + Veh mice (Supp. Fig. 5D; RT-TMZ + Shef6 EV, P ≤ 0.01; RT-TMZ + UCI-191 EV, P ≤ 0.05).

Representative images of PSD-95 in the CA1 (*pyr*, *sr*) for all male and female groups are showing dendritic expression of PSD-95^+^ emanating through the *sr* (Fig. 4E and F). Representative images for the dentate molecular layer (*dg*, *ml*) are shown in Supp. Fig. S6A–B. Volumetric quantification in the CA1 (*pyr*, *sr*) showed that RT-TMZ + Veh male mice had a significant decrease in PSD-95 compared to Control + Veh, Control + Shef6 EV and Control + UCI-191 EV (Fig. 4G; P ≤ 0.0001). RT-TMZ + Shef6 EV and RT-TMZ + UCI-191 EV male mice had a significantly elevated PSD-95 compared to RT-TMZ + Veh (Fig. 4G; P ≤ 0.0001). Similarly, RT-TMZ treated males (RT-TMZ + Veh) hippocampal dentate molecular layer had a significant decrease in PSD-95 compared to Control + Veh, Control + Shef6 EV, and Control + UCI-191 EV (Supp. Fig. S6C; P ≤ 0.0001, 0.0001, and 0.001, respectively). Both EV treated RT-TMZ groups showed improved PSD-95 immunoreactivity compared to RT-TMZ + Veh (Supp. Fig. S6C; P ≤ 0.001). As in males, female CA1 (*pyr*, *sr*) PSD-95 expression was reduced in the RT-TMZ + Veh compared to Control + Veh, Control + Shef6 EV, and Control + UCI-191 EV (Fig. 4H; P ≤ 0.0001). Both hNSC-EV treated groups had a significant increase in PSD-95 expression compared to RT-TMZ + Veh (Fig. 4H; RT-TMZ + Shef6 EV, P ≤ 0.01; RT-TMZ + UCI-191 EV, P ≤ 0.0001). Further examination of the data showed significantly higher expression in Control + UCI-191 EV compared to RT-TMZ + Shef6 EV (P ≤ 0.001), and Control + Veh compared to RT-TMZ + Shef6 EV (Fig. 4H; P ≤ 0.01). Quantification of PSD-95 expression in the female dentate molecular layer found reduced expression in the RT-TMZ + Veh group compared to Control + Veh, Control + Shef6 EV, and Control + UCI-191 EV (Supp. Fig. S6D; P ≤ 0.0001, 0.05, and 0.01). Whereas UCI-191 EV increased PSD-95 following RT-TMZ treatment in female mice compared to RT-TMZ + Veh (Supp. Fig. S6D; P ≤ 0.05); RT-TMZ + Shef6 EV showed a trend of higher PSD-95 expression, albeit not significant (Supp. Fig. S6D). These data indicate neurodegenerative impact of combined RT and TMZ treatments on synaptic density, and that hNSC-EV treatment protected against the synaptic loss.

### 3.5. hNSC-derived EVs reverse cognitive decline without interfering with the therapeutic efficacy of RT-TMZ for glioma

**Fig. 5.**
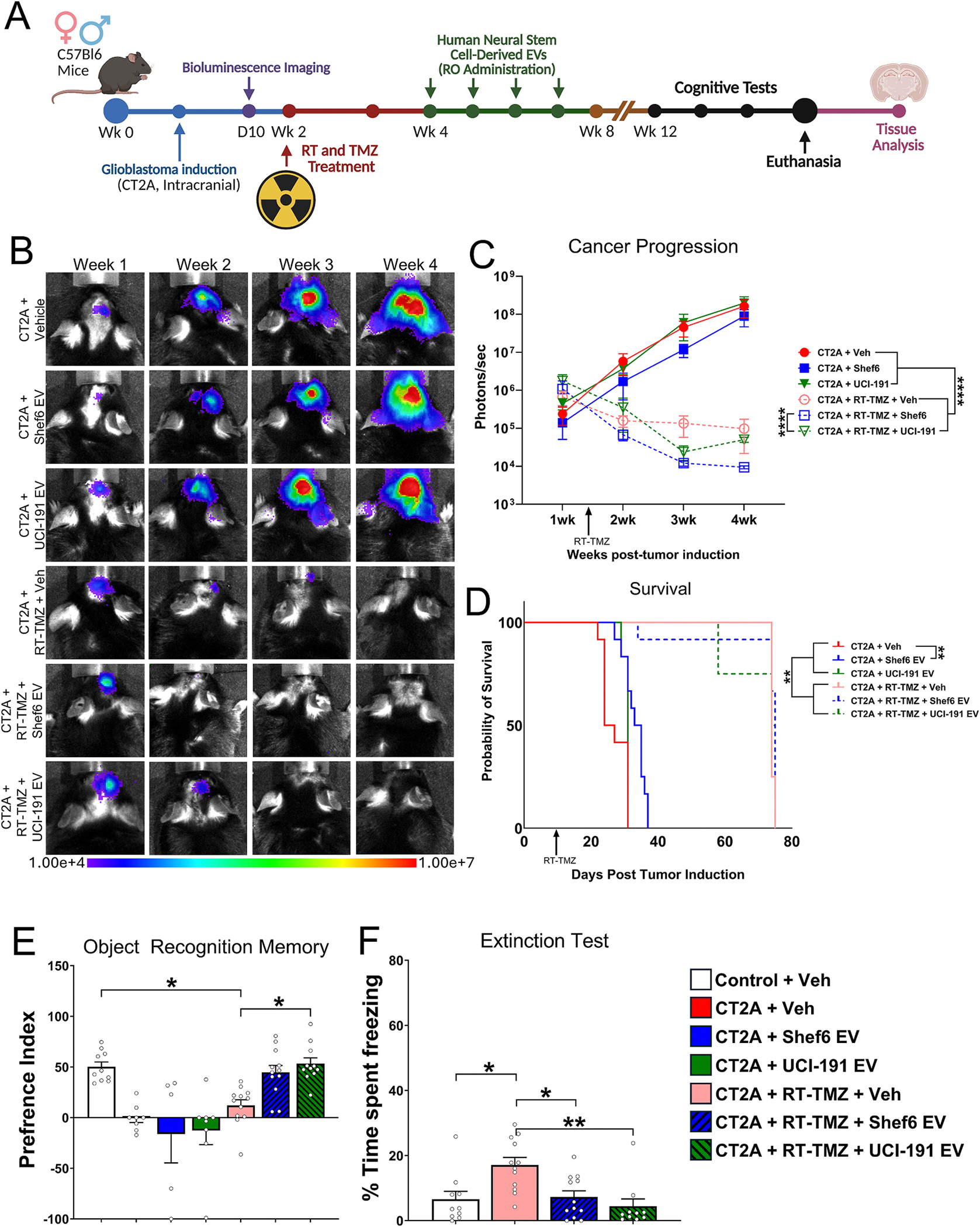
hNSC-EVs improve cognition in glioma-bearing mice without interfering with the efficacy of RT and TMZ. (**A**) Research design: Three-month-old male and female C57BL6 WT mice were intracranially injected with murine CT2A-luc cells using stereotaxic guidance. Tumor burden was confirmed ten days post-induction and monitored longitudinally via bioluminescence imaging, quantified by the IVIS^®^ Spectrum system. Following tumor confirmation, mice received combined RT and TMZ as described in Fig. 2 (CT2A + RT-TMZ + Veh). Mice were then treated with hNSC-EVs (CT2A + RT-TMZ + Shef6 EV, CT2A + RT-TMZ + UCI-191 EV), followed by cognitive and behavioral testing. After completion of cognitive testing, mice were euthanatized, and tissues were collected (designed using BioRender.com). See Suppl. Fig. S7 for OFT and EPM data. (**B**) Representative bioluminescence images depicting glioma progression over four weeks post-induction. (**C**) Tumor growth was quantified by relative bioluminescence and presented logarithmically. (**D**) Kaplan-Meier survival plot of cancer-bearing animals receiving vehicle, EV or RT-TMZ treatments. For each group represented on the survival plot *N* = 12, Males: *N* = 6–9, Females: *N* = 3–6. Mantel-Cox log-rank test (pairwise comparisons) and Bonferroni's multiple corrections. (**E**) The ORM task presented as Preference Index, calculated by: [(*T*_*Novel*_/*T*_*Total*_)-(*T*_*Familiar*_/*T*_*Total*_)] × 100, where *T* is the time (s) exploring novel or familiar object (or location), and the total time exploring both. See Suppl Table T1 for complete list of statistical significance showing P values. See Suppl. Fig. S8 for ORM heatmap. (F) Extinction testing quantified percentage time spent freezing on final Extinction Test Day. See Suppl. Fig. S9 for graphical representation of sequential fear extinction phase (Days 1–3) after fear conditioning (T1–3). Data represented by mean ± SEM. *N* = 4–12 animals per group (each group included 2–4 females). *P* values calculated by two-way ANOVA and Bonferroni's multiple comparisons test (**C**, **E, F**) and by Lognormal Brown-Forsythe and Welch ANOVA and Dunnett's T3 multiple comparisons test (**D**). *, P ≤ 0.05; **, P ≤ 0.01; ***P ≤ 0.001; ****, P ≤ 0.0001. (For interpretation of the references to color in this figure legend, the reader is referred to the Web version of this article.)

The foregoing data suggest that RO treatment with hNSC-EVs prevent fractionated RT and TMZ treatment-induced cognitive dysfunction, neuroinflammation and synaptic loss. We sought to confirm the cognitive benefit of hNSC-EVs in a clinically relevant brain cancer model. 12-week-old C57Bl/6 male and female mice were induced with syngeneic brain cancer via stereotaxic intracranial injection of the luciferase-expressing murine glioma line CT2A-luc (Fig. 5A). Confirmation of tumor development and subsequent progression or the therapeutic response to RT-TMZ treatment was monitored via BLI (Fig. 5B). Following successful tumor implantation, mice underwent clinically relevant fractionated RT and TMZ to model the Stupp protocol (described in Materials and Methods). 72h after final chemotherapy treatment, either Shef6 or UCI-191 hNSC-EVs were administered through intravenous injections (RO) once weekly for four weeks. Four weeks after the final EV injection, mice performed cognitive testing, after which they were euthanized, and tissues were collected for analysis.

Quantitative analysis of photon emittance during BLI revealed a significant difference between untreated and RT-TMZ treated glioma-bearing animals (Fig. 5C; P ≤ 0.0001). While there was no observable difference in tumor growth amongst the untreated cancer groups (CT2A + Veh, CT2A + Shef6 EV, CT2A + UCI-191 EV), treatment with RT-TMZ significantly reduced the cancer growth in CT2A + RT-TMZ + Shef6 EV compared to CT2A + RT-TMZ + UCI-191 EV (Fig. 5C; P ≤ 0.0001). Importantly, treatment with hNSC-EVs neither influenced the trajectory of the tumor growth without RT-TMZ treatment, nor interfered with the therapeutic response to RT-TMZ (Fig. 5B–D). Kaplan-Meier survival data revealed that RT-TMZ treated mice survived significantly longer than glioma-bearing mice (Fig. 5D; P ≤ 0.01), with no observable difference in survival in mice undergone Stupp protocol treatment with or without EV injections. Notably, we found an increase in survival of CT2A + Shef6 EV mice compared to CT2A + Veh (Fig. 5D; P ≤ 0.01).

Analysis of EPM testing showed that CT2A + RT-TMZ + UCI-191 EV spent a lower percentage of time in the open arms of the maze compared to CT2A + Veh and CT2A + UCI-191 EV (Supp. Fig. 7A; P ≤ 0.05). On the other hand, OFT testing showed that CT2A + RT-TMZ + Shef6 EV spent higher percentage of time in the central zone of the arena compared to CT2A + Veh and CT2A + Shef6 EV (Supp. Fig. 7B; P ≤ 0.05). After completion of EPM and OFT testing, mice were habituated for the ORM test. In absence of RT-TMZ treatment, tumor burden itself significantly reduced the performance on the ORM task for the CT2A + Veh, CT2A + Shef6 EV and CT2A + UCI-191 EV compared to Control + Veh (Supp. Table T1; CT2A + Veh, P ≤ 0.01; CT2A + Shef6 EV and CT2A + UCI-191 EV, P ≤ 0.001). RT-TMZ significantly improved PI compared to glioma-bearing mice, but CT2A + RT-TMZ + Veh did not perform up to the Control + Veh level (Fig. 5E; P ≤ 0.05). Meanwhile, UCI-191 EV treatment to the CT2A + RT-TMZ mice significantly increased the PI compared to CT2A + RT-TMZ + Veh (P ≤ 0.05), with a trend of higher PI observed in CT2A + RT-TMZ + Shef6 EV (Fig. 5E). For the sake of brevity, the significance of Control + Veh v. CT2A + RT-TMZ + Veh and CT2A + RT-TMZ + Veh v. CT2A + RT-TMZ + UCI-191 EV are shown on the plot (Fig. 5E); see Supp. Table 1 for all other group comparisons with significance. These findings are also represented in the heatmap of animals exploring novel and familiar objects in the ORM task (Supp. Fig. S8).

Animals were then administered FE memory consolidation test to assess hippocampal-cortical-amygdala neurocircuitry. Due to the invasive nature of the task (foot shocks and tones), we only tested Control + Veh, CT2A + RT-TMZ + Veh, CT2A + RT-TMZ + Shef6 EV, and CT2A + RT-TMZ + UCI-191 EV groups. All animals successfully acquired the conditioned fear response as evidenced by an increase in percentage of freezing during *Conditioning* (*T*_*1*_*-T*_*3*_; Supp. Fig. S9). Throughout *Extinction Training (Day* 1-Day *3)*, CT2A + RT-TMZ + Veh had a higher percentage of freezing compared to Control + Veh (Supp. Fig. S9), this was also observed on Test Day with CT2A + RT-TMZ + Veh having a higher percentage of freezing compared to Control + Veh (P ≤ 0.05). During *Extinction Test*, both CT2A + RT-TMZ + Shef6 EV and CT2A + RT-TMZ + UCI-191 EV had a lower percentage of freezing compared to CT2A + RT-TMZ + Veh (Fig. 5F; P ≤ 0.05 and 0.01, respectively). This data confirms the detrimental impact of cancer, cranial irradiation and chemotherapy on cognition that was restored by hNSC-EV treatment.

### 3.6. Neuroinflammation following combined RT and TMZ treatment in the glioma model

**Fig. 6.**
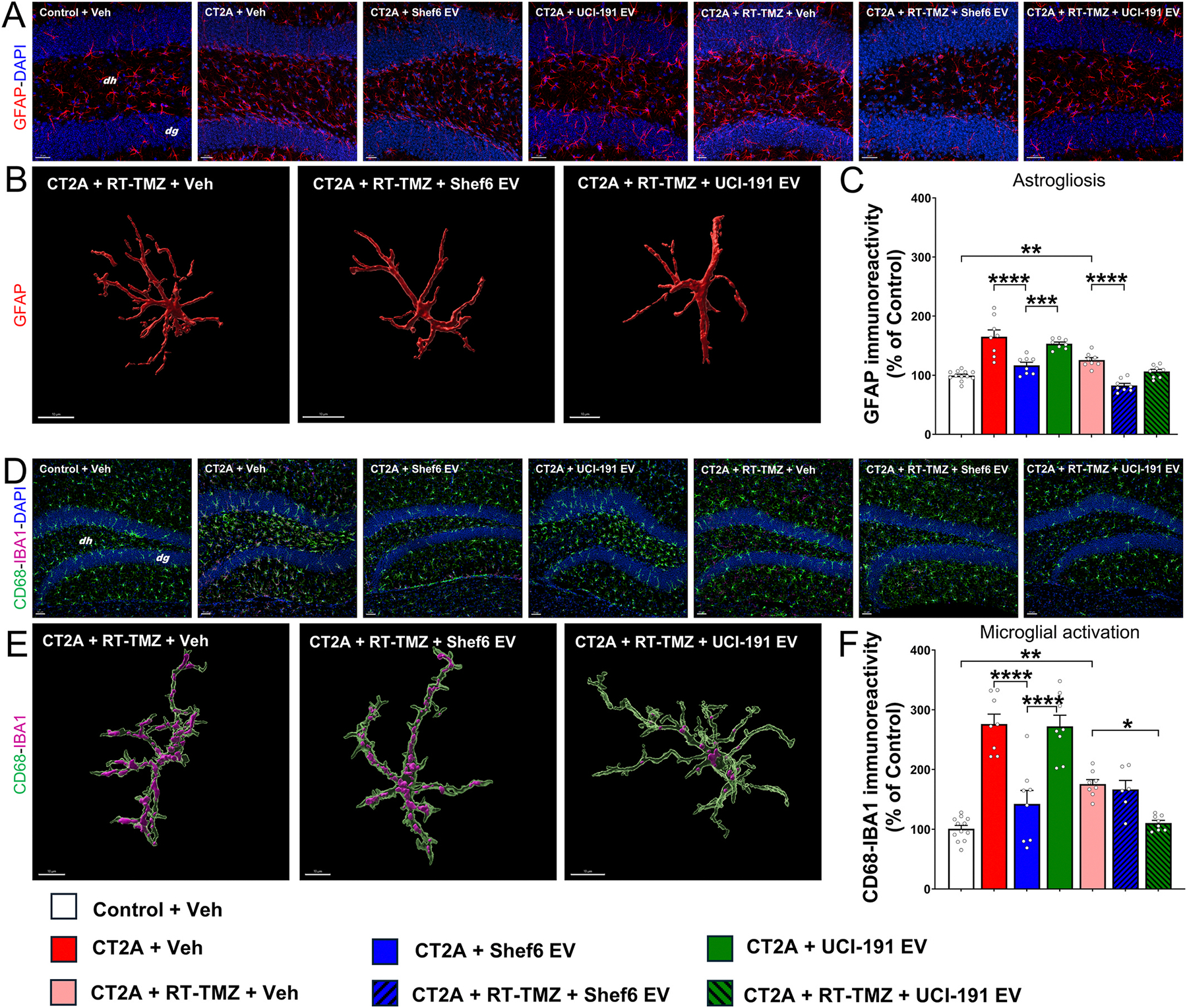
hNSC-EVs reduce neuroinflammation in brain cancer-bearing mice receiving combined cranial radiation therapy and chemotherapy. (**A**-**C**) Visual representation and quantification of astrogliosis. Immunofluorescence staining, confocal microscopy, and 3D algorithm-based quantification for GFAP (red) with a DAPI nuclear counter stain (blue) in the dentate hilus (*dh*) and dentate gyrus (*dg*) (**A** and **C**). See Suppl Table T2 for complete list of significant test comparisons. (**B**) High resolution GFAP^+^ surface reconstruction for the CT2A + RT-TMZ + Veh, CT2A + RT-TMZ + Shef6 EV, and CT2A + RT-TMZ + UCI-191 EV groups. (**D**-**F**) Visual depiction and analysis of microglial activation. CD68-IBA1 dual immunofluorescence staining with DAPI nuclear counter stain (blue) in the dentate hilus (*dh*) and dentate gyrus (*dg*). (**E**) High resolution surface rendering of CD68-IBA1 colocalization is shown for CT2A + RT-TMZ + Veh, CT2A + RT-TMZ + Shef6 EV, and CT2A + RT-TMZ + UCI-191 EV groups. Confocal z stacks were acquired at 40 × (**A**, 1.30 numerical aperture) and 20 × (**D**, 0.75 numerical aperture) magnifications. Scale bars, 30 μm (**A**), 10 μm (**B** and **E**), and 50 μm (**D**)**.** Data are represented by mean ± SEM (N = 5–12 animals/group). *P* values were calculated by one-way ANOVA and Tukey's multiple comparisons tests. *, P ≤ 0.05; **, P ≤ 0.01; ***P ≤ 0.001; ****, P ≤ 0.0001. (For interpretation of the references to color in this figure legend, the reader is referred to the Web version of this article.)

Analysis of GFAP immunoreactivity showed that, compared to Control + Veh, CT2A + RT-TMZ + Veh exhibited higher expression (P ≤ 0.01), while CT2A + RT-TMZ + Shef6 EV had reduced GFAP compared to CT2A + RT-TMZ + Veh (Fig. 6A–C; P ≤ 0.0001). Interestingly, CT2A + Shef6 EV had decreased GFAP expression compared to CT2A + Veh and CT2A + UCI-191 EV (Fig. 6A–C; P ≤ 0.0001 and 0.001, respectively). For the sake of brevity, all other group comparisons are shown in Supp. Table 2. High resolution surface reconstruction of single astrocytes distinguishes between hypertrophic morphology in the CT2A + RT-TMZ + Veh compared to EV treated groups (Fig. 6B). Quantification of microglial activation using CD68-IBA1 dual immunofluorescence showed that CT2A + RT-TMZ + Veh displayed higher colocalization compared to Controls (Fig. 6D–F; P ≤ 0.01). CT2A + RT-TMZ + UCI-191 EV showed reduced microglial activation compared to CT2A + RT-TMZ + Veh (Fig. 6D–F; P ≤ 0.05). All other P values are shown in Supp. Table 3. Visualization of microglial morphology highlights the elevated cellular expression of CD68 in CT2A + RT-TMZ + Veh compared to EV treated groups (Fig. 6E).

### 3.7. hNSC-EVs protect against synaptic loss following the glioma chemoradiotherapy

**Fig. 7.**
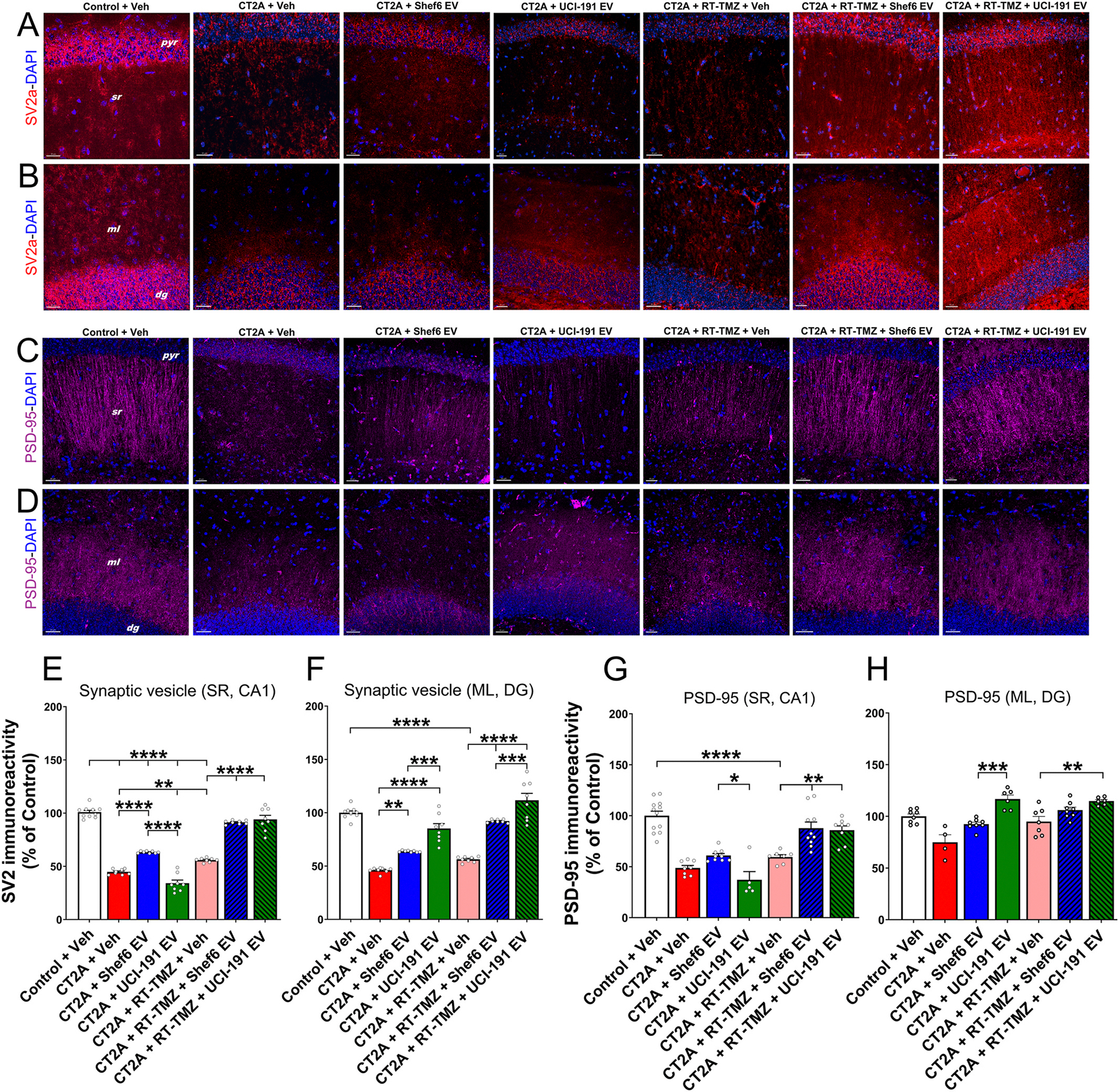
hNSC-EVs restore synaptic density in the glioma-bearing brain treated with combined radiation and chemotherapy. (**A**-**B**) Immunofluorescent confocal micrographs of synaptic vesicle glycoprotein 2A (SV2a; red) with DAPI nuclear counter stain (blue) in the pyramidal cell layer (*pyr*) and stratum radiatum (*sr*) of CA1 (**A**) and the molecular layer (*ml*) and dentate gyrus (*dg*) in the hippocampus (**B**). (**C**-**D**) Fluorescent confocal micrographs of postsynaptic density protein 95 (PSD-95; magenta) with DAPI nuclear counter stain (blue) in the pyramidal cell layer (*pyr*) and stratum radiatum (*sr*) of CA1 (**C**) and the molecular layer (*ml*) and dentate gyrus (*dg*) in the hippocampus (**D**). (**E**-**F**) Volumetric quantification of SV2a immunoreactive puncta in the CA1 *sr* (**E**) and *ml dg* (**F**) subregions. See Suppl Table T4 –T5 for all the group comparisons with significance. (G-H) Volumetric quantification of PSD-95 immunoreactive puncta CA1 (G) and dg (H). See Suppl Table T6-T7 for all the group comparisons with significance. Scale bars, 30 μm (**A**-**D**). Data are represented by mean ± SEM (N = 4–8 animals/group). *P* values were calculated by one-way ANOVA and Tukey's multiple comparisons tests. *, P ≤ 0.05; **, P ≤ 0.01; ***P ≤ 0.001; ****, P ≤ 0.0001. (For interpretation of the references to color in this figure legend, the reader is referred to the Web version of this article.)

Analysis of SV2a in the CA1 showed reduced expression in the CT2A + RT-TMZ + Veh compared to Control + Veh group (Fig. 7E; P ≤ 0.0001). Concurrently, both EV-treated CT2A and CT2A + Veh groups showed reduced SV2a expression compared to the Control + Veh group (Fig. 7E; P's ≤ 0.0001). In contrast, glioma-bearing mice receiving RT-TMZ and subsequently hNSC-EVs, had significantly higher SV2a expression compared to CT2A + RT-TMZ + Veh (Fig. 7E; P's ≤ 0.0001; see Supp Table T4 for all other group comparisons with significance). SV2a expression in the dentate molecular layer was significantly lower in CT2A + RT-TMZ + Veh mice compared to Control + Veh (Fig. 7F; P ≤ 0.0001). CT2A + RT-TMZ + Shef6 EV and CT2A + RT-TMZ + UCI-191 EV had elevated SV2a compared to CT2A + RT-TMZ + Veh (Fig. 7F; P ≤ 0.0001). Between the two EV treated groups receiving RT-TMZ, CT2A + RT-TMZ + UCI-191 EV had higher SV2a expression compared to CT2A + RT-TMZ + Shef6 EV (Fig. 7F; P ≤ 0.001; see Supp Table T5, for all other group comparisons with significance).

Quantification of PSD-95 immunoreactivity in the *pyr* and *sr* regions of CA1 revealed significantly lower expression in CT2A + RT-TMZ + Veh mice compared to Control + Veh (Fig. 7G; P ≤ 0.0001). Both CT2A + RT-TMZ + Shef6 EV and CT2A + RT-TMZ + UCI-191 EV had higher PSD-95 expression compared to CT2A + RT-TMZ + Veh (Fig. 7G; P ≤ 0.01; see Supp Table T6, for all other group comparisons with significance). PSD-95 expression in the dentate molecular was higher in CT2A + RT-TMZ + UCI-191 EV compared to CT2A + RT-TMZ + Veh (Fig. 7H; P ≤ 0.01; see Supp Table T7 for all other group comparisons with significance). Overall, treatment with hNSC-derived EVs restored or increased synaptic density in glioma-bearing mice receiving chemoradiotherapy.

### 3.8. hNSC-EV elevates neuroprotective gene signatures following RT-TMZ treatment for glioma

To characterize the transcriptional signatures and analyze the functional relevance of statistically significant genes in response to RT-TMZ treatment, volcano plots and bubble plots were generated across experimental groups using differential expression datasets derived from log_2_ fold-change expression files. Volcano plot analysis revealed significant upregulation of *Gzmb*, *Mapk12, Slamf8, Ppp3r2,* and *Il15ra* in the RT-TMZ + Shef6 EV group compared with the RT-TMZ + Veh. In contrast, the RT-TMZ + UCI-191 EV group exhibited increased expression of genes associated with cellular homeostasis and differentiation, including *Eomes, Prkcq, Gzma, Ccl7, Pilrb1, Fbln5,* and *Cdkn1c*. Functional assessment using bubble plots further demonstrated increased expression of *Arc*, *Tbr1*, and *cFos* following Shef6 EV treatment in the context of RT-TMZ exposure. In comparison, UCI-191 EV treatment following RT-TMZ exposure showed higher expression of several genes, including *Dab2, Pilrb1, Dlx 2,* and *Cst7.* However, these changes showed low statistical significance.

In cancer-bearing and RT-TMZ-exposed mouse brains, Shef6 EV treatment resulted in upregulation of *Osmr, Itga6, Dock2,* and *Fbln5*, genes implicated in tissue remodeling and neuroprotective processes. However, Shef6 EV treatment was also associated with downregulation of key homeostatic markers, including *Fos* and *Foxp3*. Bubble plot analysis further revealed increased expression of *Cp* and *Osmr* following Shef6 EV treatment in cancer-bearing irradiated mice. In contrast, UCI-191 EV treatment in cancer-bearing irradiated mice was associated with upregulation of *Slc2a1 (GLUT1), Ly6a, Tie1,* and *Fbln5*, along with downregulation of *Cd19* and *Pnoc*, and reduced expression of *Ccl3*. Collectively, these transcriptional changes suggest that UCI-191 EV treatment confers a pronounced neuroprotective and immunomodulatory effect compared with Shef6 EVs in RT-TMZ-exposed and tumor-bearing mouse brains.

### 3.9. Systemic hNSC-EV treatment did not impact peripheral organs

To determine the impact of systemic hNSC-EV delivery via RO injections on the gross pathologic outcomes of some of the key peripheral organs, we conducted H&E staining on liver, lung and kidneys. Histopathologic assessment did not reveal any gross treatment-related abnormalities in peripheral organs (Suppl. Fig. S10–S12). Liver, lung, and kidneys demonstrated preserved tissue architecture and cellular morphology in both tumor-bearing and non-tumor-bearing mice across all treatment arms, including RT-TMZ and hNSC-EV cohorts.

## 4. Discussion

This study demonstrates that systemically delivered EVs from GMP-grade hNSCs ameliorate glioma chemoradiotherapy-induced neurocognitive dysfunction while preserving, and in some contexts augmenting, anti-tumor efficacy in a clinically relevant glioma Stupp protocol model. Building on our prior work using stem cell transplantation and hNSC-EVs to reverse radiation- or chemotherapy-induced cognitive decline, these data support a mechanistic shift from cell replacement to EV-mediated neurotrophic and immunomodulatory support as a more feasible, acellular therapeutic strategy for CRCI [17–19,27,40–44]. The Shef6 cells line was previously tested for its neuroprotective benefits in rat [32] and marmoset model of TBI. UCI-191 cell line is currently being tested for the treatment of spinal cord injury and multiple sclerosis. Given the substantial translationally feasible work using these hNSCs, we sought to investigate the therapeutic benefits of EVs isolated from these two cells lines.

### 4.1. Regenerative approaches to alleviate CRCI

We and others have previously shown that transplantation of embryonic, fetal-derived, and commercially available hNSCs restore cognitive function after cranial RT or chemotherapy [17–19,27,32, 40–44]. Albeit a majority of these studies utilized an athymic nude rat model. Long-term follow-up revealed that only a small fraction of grafted hNSCs survived and very few differentiated into mature neurons, indicating that the durable cognitive rescue could not be attributed to direct cell replacement [19]. Instead, these findings, together with independent literature, implicated paracrine neurotrophic and anti-inflammatory benefits of transplanted stem cells as the primary driver of functional recovery [26,45,46].

To directly test this paracrine hypothesis, we previously administered a commercially available hNSC-derived EVs in an acute RT rodent model and showed that EVs alone recapitulated the benefits of cell transplantation, improving cognition, reducing neuroinflammation, and enhancing dendritic complexity [27,45]. Importantly, those studies also demonstrated that hNSC-EVs migrated through the host brain, colocalized with neuronal and astrocytic nuclei, and delivered bioactive cargo that restores neuronal plasticity, providing functional equivalence between stem cells and EVs in the irradiated brain [26,27,45,47]. The present work extends this regenerative paradigm into an immunocompetent, syngeneic glioma model treated with fractionated RT-TMZ, thereby aligning more closely with the clinical standard of care for glioblastoma [48]. Importantly, we utilized GMP-ready hNSC lines that are already being advanced toward clinical trials for traumatic brain injury and spinal cord injury, underscoring translational readiness. We rigorously verified EV biophysical properties, including size distribution, yield, and EV-specific protein biomarker expression, across multiple stem cell batches and passages (Fig. 1) [49–51], providing confidence in the robust and reproducible EV preparations suitable for eventual clinical implementation.

### 4.2. EVs mitigate RT-TMZ-induced neurotoxicity in non-tumor and glioma-bearing brains

In the WT male and female mice, fractionated RT combined with clinically relevant dosing of TMZ induced robust and persistent impairments across hippocampal- and cortico-amygdala-dependent domains, including ORM, OLM, and FE, as well as increased anxiety-like behavior. We observed similar neurocognitive impairments previously following exposure to acute RT or chemotherapy [52–56]. Systemic RO administration of Shef6 or UCI-191 EVs after completion of RT-TMZ restored performance on these cognitive function tasks in both sexes, indicating preserved episodic, spatial, and memory consolidation (Fig. 2) [57–59]. These neurocognitive improvements paralleled a marked reduction in astrogliosis and microglial activation (Fig. 3), and a restoration of synaptic density in the hippocampal CA1 and dentate gyrus (Fig. 4; Supp. Fig. S5–6), as indexed by SV2a and PSD-95 immunoreactivity – hallmarks of the neurodegeneration that predict progressive cognitive decline in patients receiving RT and systemic chemotherapy [52,54,56].

**Fig. 8.**
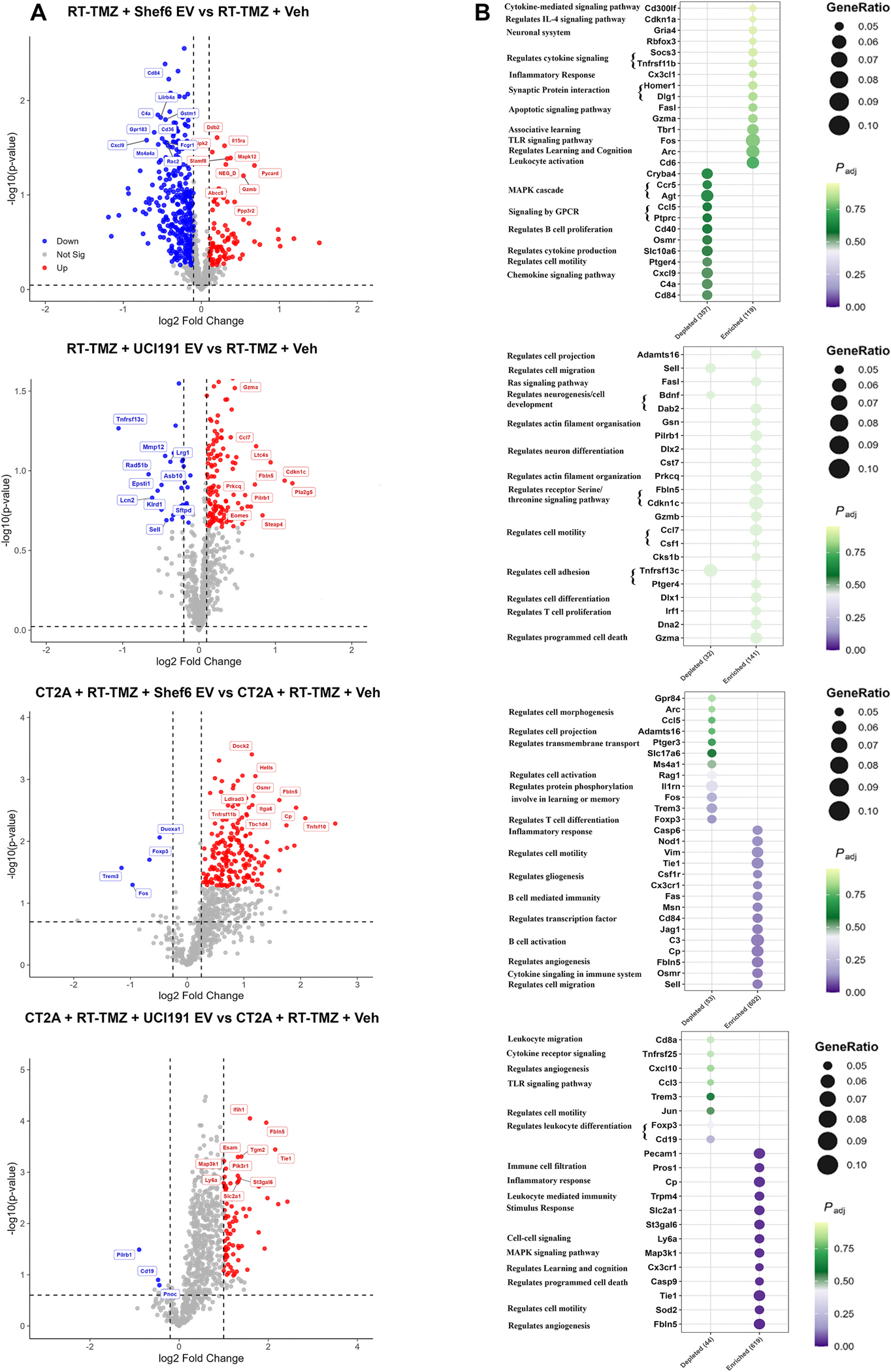
Comparative gene expression analysis of cancer and non-cancer mice hippocampi treated with hNSC-EVs. (**A**) Volcano plots depict differential gene expression in the host hippocampus across the experimental groups, including RT-TMZ + Shef6 EV (N = 3), RT-TMZ + UCI-191 EV (N = 3), CT2A + RT-TMZ + Shef6 EV (N = 3), and CT2A + RT-TMZ + UCI-191 EV (N = 3), each compared with their corresponding controls, with each point representing an individual gene. The x-axis corresponds to the log2 fold change (magnitude of expression difference between conditions), while the y-axis corresponds to the −log 10 of the adjusted P value (statistical significance after multiple testing correction, Benjamini-Hochberg FDR). Downregulated genes migrate further to the left, while upregulated genes displayed further to the right. Higher −log_10_P values indicated higher statistically significant gene expression. Significance thresholds used were adjusted P < 0.05 and log2 fold change >1. Red: Strongly upregulated, high significance. Blue: Strongly downregulated, high significance. (**B**) Bubble plots of differentially expressed genes (DEGs) in the host hippocampus in RT-TMZ + Shef6 EV, RT-TMZ + UCI-191 EV, CT2A + RT-TMZ + Shef6 EV, and CT2A + RT-TMZ + UCI-191 EV compared with Control + Veh (N = 4). Bubble plot showing significantly enriched and depleted genes across the experimental group relative to Control. Each bubble represents an individual gene meeting the criteria |log_2_ fold change| > 0.2 and adjusted P < 0.5. Genes are grouped by regulation status (Enriched or Depleted) and ordered by statistical significance (−log_10_ P value). Bubble size corresponds to the scaled magnitude of differential expression (GeneRatio derived from |log_2_ fold change|), while bubble color indicates the adjusted P value, with darker colors representing higher statistical significance. Gene ontology terms displayed along the x-axis denote Metascape-derived functional enrichment pathways associated with each gene. (For interpretation of the references to color in this figure legend, the reader is referred to the Web version of this article.)

We then determined if hNSC-EVs could confer equivalent neurocognitive benefits in the context of clinically relevant glioblastoma model treated with the Stupp regimen. In glioma-bearing mice, tumor burden alone impaired object recognition and fear extinction memory consolidation processes and was associated with elevated gliosis and the loss of synaptic density, mirroring cognitive and neuropathologic findings in patients with glioma (Fig. 5) [60]. As expected, RT-TMZ treatment led to a significant therapeutic response in shrinking tumors *in vivo* and improved survival but further impaired cognitive performance and exacerbated neuroinflammation and synaptic loss relative to non-tumor controls, recapitulating the dual-edged nature of current glioma therapy [61,62]. Notably, both Shef6 and UCI-191 EVs administered after chemoradiotherapy improved cognitive performance and restored synaptic and glial markers without attenuating the anti-tumor efficacy of RT-TMZ, demonstrating that hNSC-EVs can uncouple oncologic benefit from CNS toxicity in this model. Along with these benefits, we also observed the impact of Shef6 EVs on therapeutic response and overall survival. We found that the cancer-bearing groups treated with RT-TMZ still showed an intact therapeutic response in both groups treated with EVs (CT2A + RT-TMZ + Shef6 EV and CT2A + RT-TMZ + UCI-191 EV) confirming that EV treatment does not protect the tumor; in fact, Shef6 EVs significantly improved the therapeutic response to RT-TMZ (Fig. 5C). We have also confirmed that the EV treatment did not promote tumor growth by including cancer + EV groups not receiving RT-TMZ (CT2A + Shef6 EV and CT2A + UCI-191 EV). Our data shows that the cancer growth was not increased or accelerated compared to CT2A + Veh, and that Shef6 EV treatment slows the overall progression of cancer growth. Given the lack of adverse tumor burden caused by the EVs, we assume there is no significant effect on genotoxic stress. Gene expression analysis (Fig. 8) found inflammatory and immune-responsive signatures in the host tissue that would modulate tumor response *in vivo*. For example, in the RT-TMZ-exposed, cancer-bearing brain, Shef6 EV increased *Gzmb, Il15ra, Mapk12, Slamf8,* and *Ppp3r2*, indicating a strong cytotoxic immune response and *Osmr* and *Cp* (ceruloplasmin), which are linked to oxidative phosphorylation, reactive oxygen species (ROS), DNA-damage signaling, and radiation resistance in tumor cells. However, we didn't conduct gene expression analysis of cancer-bearing mice receiving Shef6-EV as this group was not clinically relevant in context of the cancer therapy (RT + TMZ), which is the likely scenario for the application of neuroprotective strategy to alleviate cognitive impairments. Importantly, while animal survival (Fig. 5D) was comparable between the two EV types (Cancer + RT-TMZ + EV), the cancer growth data (BLI, Fig. 5C) indicated significantly reduced cancer growth at 4 weeks post-implantation in the cancer + RT-TMZ + Shef6 EV groups compared to UCI-191 EV-treated group indicating a tumor modulatory and neuroprotective impact *in vivo*.

### 4.3. EVs restore functional and transcriptional neuroprotective signatures

Transcriptomic profiling of RT-TMZ–exposed non-tumor brains revealed that Shef6 and UCI-191 EVs engage distinct yet convergent neuroimmune programs that align with the restored cognitive function and reduced neurodegenerative consequences (Figs. 2–4, Fig. 8). In the RT–TMZ + Shef6 EV group, immune-activating genes such as *Gzmb, Mapk12, Slamf8, Ppp3r2,* and *Il15ra* were upregulated, indicating enhanced cytotoxic, cytokine, and T/NK cell-related signaling compared with RT-TMZ + Veh [63–66]. Upregulation of plasticity-related genes including *Arc, Tbr1,* and *Fos*, together with downregulation of chemokine/MAPK pathway components such as *Ccr 5, Cxcl9,* and *Agt*, suggests that Shef6 EVs simultaneously promote activity-dependent neuronal remodeling and dampen RT-TMZ-induced inflammatory cascades [67–72]. Collectively, these changes point to an EV-driven, immune-activating milieu that could facilitate cytotoxic engagement with neuroplastic and tissue-repair-related gene programs, favoring anti-tumor immunity or protecting neural tissue after irradiation. In parallel, RT-TMZ + UCI-191 EV increased expression of genes linked to T cell differentiation and immune modulation (*Eomes, Prkcq, Gzma, Ccl7, Pilrb1*) while elevating neuroprotective and homeostatic factors such as RAS signaling (e.g., *Fasl*) and the serine/threonine signaling pathway (*Fbln5* and *Cdkn1c*), implicated in neuronal plasticity, vascular/extracellular matrix (ECM) support, and controlled cell survival [73–77]. Notably, the higher expression of these genes suggests preserved neural integrity, enhanced neurogenesis, and activation of cytoprotective pathways, indicating a cellular shift toward survival and repair in irradiated neural tissue. Together, these gene expression profiles support a model in which Shef6 EVs promote mixed protective effect with robust immune activation and synaptic plasticity, whereas UCI-191 EVs favor neuroprotective and regenerative response post RT-TMZ exposure, although both treatments show partial evidence of protection against radiation induced injury.

In glioma-bearing mice, EV-responsive gene networks further illuminate downstream interactions of EVs with the tumor-bearing irradiated brain (Figs. 5–8). In CT2A + RT-TMZ mice, Shef6 EV treatment increased expression of *Osmr, Itga6, Dock2, Fbln5,* and *Tnfsf10,* genes involved in JAK/STAT and MAPK signaling, immune synapse formation, tissue remodeling, and apoptosis, suggesting a combination of neuroprotective, reparative, and cytotoxic programs [78–80]. However, Shef6 EVs also reduced expression of homeostatic regulators such as *Fos* and *Foxp3* and increased *Cp*, and *Osmr*, both previously linked to enhanced oxidative phosphorylation, ROS production, and activation of DNA damage-response pathways that can support glioma survival and radio-resistance [67,81,82]. Overall, in cancer-bearing brains, Shef6 EVs reveal a more complex, dual-edged profile although they may enhance cytotoxic and immune-related responses that could help restrain tumors, they also activate metabolic and stress-adaptive pathways, such as *Osmr*-driven OXPHOS and *Cp*-associated redox signaling, which may potentially support tumor resistance. In contrast, CT2A + RT-TMZ + UCI-191 EV brains showed upregulation of *Slc2a1, Ly6a, Tie1,* and *Fbln5* and downregulation of *Cd19, Pnoc,* and *Ccl3,* an expression profile consistent with improved glucose handling and metabolic support, vascular integrity and angiogenesis, synaptic plasticity, and attenuation of B cell- and chemokine-driven inflammatory signaling [45,76,77,83–85]. The gene profile suggests that UCI-191 EVs promote cell survival, vascular repair, and tissue recovery, while also dampening some immune-inflammatory signals, favoring predominantly a protective and anti-tumor immunomodulatory effect in cancer bearing irradiated brains.

In our mouse model of glioma chemoradiation therapy, we found divergent benefits imparted by each type of EV. For example, Shef6-EV significantly improved survival in vehicle- and 0 Gy-treated mice (Fig. 5D). EVs carries biologically distinct active cargo that differs depending on their cell origin (for example, Fig. 1G). Given the passive assortment of biological cargo into EVs, we expect to observe functional and molecular (transcriptomic) differences in the host brain. For example, distinct gene expression patterns was observed in non-cancer and cancer-bearing mice receiving RT + TMZ + EV (Fig. 8). Based on gene cluster comparison, Shef6 EVs in the RT-TMZ and cancer-bearing irradiated mice, increases *Gzmb, Il15ra, Mapk12, Slamf8,* and *Ppp3r2*, which is consistent with a strong cytotoxic immune response and *Osmr* and *Cp*, which are linked to oxidative phosphorylation, ROS, DNA-damage signaling, and radiation resistance in tumor cells. These data suggest tumor modulation rather than direct impact on the host brain function following Shef6 EV treatment. Whereas UCI-191 EVs increases *Slc2a1, Ly6a, Tie1,* and *Fbln5*, which together point to improved metabolic support, regenerative activity, vascular stability, and tissue repair, alongside lowering *Ccl3, Cd19,* and *Pnoc*, indicating a controlled repair response and reduced inflammatory or tissue macrophage–associated signaling. The overall gene expression pattern suggests reduced radiation-driven inflammatory response and more homeostatic recovery following UCI-191 EV treatment. Taken together, the gene-expression data provides a mechanistic link between EV treatment and the observed functional improvements in cognition, neuroinflammation, and synaptic density. The transcriptomic profiles suggest that Shef6 EVs induces a pro-immune and stress-induced response in both non-cancer and cancer-bearing models. In contrast, UCI-191 EVs appear to promote a neuroprotective program, vascular support, and tissue homeostasis. These findings support the concept that hNSC-EVs act as cargo-driven modulators of neuroimmune and synaptic networks in non-cancer and cancer bearing RT-TMZ-treated brains, with UCI-191 EVs showing a better balance between protection of normal tissue and avoidance of tumor-supportive signaling in glioma-bearing host brains. However, mechanism-guided combination strategies, together with protein-level validation and rigorous *in vivo* approaches such as single-cell or spatial transcriptomics, are needed to define the full therapeutic potential of hNSC-derived EVs.

Overall, these transcriptomic signatures reinforce the concept that hNSC-EVs act as cargo-driven modulators of neuroimmune and synaptic networks in RT-TMZ-treated brains and suggest that UCI-191 EVs may offer a more favorable balance between neuroprotection and avoidance of tumor-supportive signaling in glioma-bearing hosts.

### 4.4. Mechanistic insights

The observation that EV treatment restores cognition, reduces gliosis, and rescues synaptic loss in the absence of substantial long-term graft survival or neuronal differentiation substantiates a model in which EV cargo (rather than engraftment *per se*) mediates the durable neuroprotective benefits of stem cell-based interventions in the irradiated brain [19,27,40]. Our previous studies identified EV-derived miR-124 as a key therapeutic cargo that, when overexpressed via AAV in acutely-irradiated WT mice, improved spontaneous exploration behavior and attenuated microglial activation, directly linking an EV-associated miRNA cargo to cognitive and neuroinflammatory outcomes [45]. The present transcriptomic analyses extend these mechanistic insights by revealing coordinated regulation of cytokine, chemokine, MAPK, and T cell-associated pathways following EV treatment in RT-TMZ expose non-cancer and cancer-bearing brains, providing a systems-level view of EV-induced network reprogramming in the injured and tumor-bearing brain [86,87]. Notably, the preservation of RT-TMZ anti-tumor efficacy across both EV treatments distinguishes this approach from many neuroprotective strategies that risk shielding tumor cells from genotoxic and cytotoxic therapies [60]. The enhanced survival observed in CT2A-bearing mice receiving Shef6 EVs, even in the absence of RT-TMZ, suggests that, under some conditions, hNSC-EVs may directly modulate tumor-immune interactions to restrict glioma progression, a possibility consistent with EV-mediated enhancement of cytotoxic gene signatures in non-tumor brains [81,88, 89]. However, this possibility is yet to be confirmed experimentally. At the same time, EV-induced upregulation of *Osmr* and *Cp* in tumor-bearing brains underscores the need for careful mechanistic dissection of EV cargo and context-dependent signaling to avoid inadvertently promoting tumor resilience [76–78,81,82]. Further, comparing EV-derived miRNA and protein content will allow us to determine the mechanism of action (biological targets) of these EVs which can be further characterized by AAV-mediated *in vivo* expression systems in future studies (as shown previously [45]).

### 4.5. Limitations and future directions

A key limitation of this work is that EVs represent heterogeneous carriers of proteins, lipids, and nucleic acids, making it challenging to pinpoint which components drive the observed neurocognitive and molecular effects. Future studies will profile protein and miRNA cargo from Shef6 and UCI-191 EVs that would directly attribute specific phenotypes to individual cargo species or pathways. Our planned experiments using AAV-mediated overexpression of candidate miRNAs enriched in EVs isolated from GMP-grade hNSC preparations, including but not limited to miR-124, will enable causal testing of discrete EV cargos in the glioma RT-TMZ treatment models and may guide rational engineering of “next-generation” EV-mimetics optimized for CRCI [45, 46]. In addition, future work should systematically compare dosing schedules and routes of administration of hNSC-EVs to refine therapeutic windows that maximize neuroprotection while minimizing any risk of tumor-supportive signaling in glioma-bearing host environment.

## 5. Conclusion

In summary, by leveraging GMP-compliant hNSC sources and a clinically relevant Stupp protocol in immunocompetent glioma-bearing mice, this study provides preclinical *proof-of-concept* that hNSC-EVs can safely and effectively remediate glioma therapy-induced brain fog. These findings position hNSC-EVs as a promising acellular, translatable platform to restore cognition and synaptic health in brain cancer survivors without compromising oncologic outcomes and thereby laying groundwork for cargo-focused mechanistic studies and, ultimately, early-phase clinical translation.

## Supplementary Material

Supplementary Data

## Data Availability

The datasets generated during and/or analyzed during the current study are available from the corresponding author upon request. The Gene Expression Omnibus (GEO) reference number for the presented RNA sequencing study is GSE319089.

## Appendix A. Supplementary data

Supplementary data to this article can be found online at https://doi.org/10.1016/j.canlet.2026.218564.

## References

[1] SEER [Internet]. [cited 2025 Oct 17], Cancer of the brain and other nervous System - cancer stat facts, Available from: https://seer.cancer.gov/statfacts/html/brain.html.

[2] Stupp R, Hegi ME, Mason WP, van den Bent MJ, Taphoorn MJB, Janzer RC, , Effects of radiotherapy with concomitant and adjuvant temozolomide versus radiotherapy alone on survival in glioblastoma in a randomised phase III study: 5-year analysis of the EORTC-NCIC trial, Lancet Oncol. 10 (5) (2009 May) 459–466, 10.1016/S1470-2045(09)70025-7. 19269895

[3] Shamsesfandabadi P, Patel A, Liang Y, Shepard MJ, Wegner RE, Radiation-Induced cognitive decline: challenges and solutions, Cancer Manag. Res. 16 (2024 Aug 21) 1043–1052, 10.2147/CMAR.S441360. 39183756 PMC11345022

[4] Hussaini S, Jang M, Effect of temozolomide on neurogenesis and anxiety in the adult mouse brain, J. Clin. Oncol. 37 (15_suppl) (2019 May 20), 10.1200/JCO.2019.37.15_suppl.e23082 e23082–e23082.

[5] Oeffinger KC, Mertens AC, Sklar CA, Kawashima T, Hudson MM, Meadows AT, , Chronic health conditions in adult survivors of childhood cancer, N. Engl. J. Med. 355 (15) (2006 Oct 12) 1572–1582, 10.1056/NEJMsa060185. 17035650

[6] Johnson DR, Sawyer AM, Meyers CA, O'Neill BP, Wefel JS, Early measures of cognitive function predict survival in patients with newly diagnosed glioblastoma, Neuro Oncol. 14 (6) (2012 Jun) 808–816, 10.1093/neuonc/nos082. 22508762 PMC3367851

[7] Greene-Schloesser D, Robbins ME, Peiffer AM, Shaw EG, Wheeler KT, Chan MD, Radiation-induced brain injury: a review, Front. Oncol. 2 (2012) 73, 10.3389/fonc.2012.00073. 22833841 PMC3400082

[8] Silber JH, Radcliffe J, Peckham V, Perilongo G, Kishnani P, Fridman M, , Whole-brain irradiation and decline in intelligence: the influence of dose and age on IQ score, J. Clin. Oncol. (1992 Sep), 10.1200/JCO.1992.10.9.1390. Located at: world. 1517781

[9] Treatment and prognosis of IDH-mutant astrocytomas in adults - UpToDate [Internet]. [cited 2025 Oct 27]. Available from: https://www.uptodate.com/contents/treatment-and-prognosis-of-idh-mutant-astrocytomas-in-adults.

[10] Abels ER, Breakefield XO, Introduction to extracellular vesicles: biogenesis, RNA cargo selection, content, release, and uptake, Cell. Mol. Neurobiol. 36 (3) (2016 Apr 6) 301–312, 10.1007/s10571-016-0366-z. 27053351 PMC5546313

[11] Théry C, Exosomes: secreted vesicles and intercellular communications, F1000 Biol Rep 3 (2011 Jul 1) 15, 10.3410/B3-15. 21876726 PMC3155154

[12] Kumar MA, Baba SK, Sadida HQ, Marzooqi SA, Jerobin J, Altemani FH, , Extracellular vesicles as tools and targets in therapy for diseases, Signal Transduct. Targeted Ther. 9 (1) (2024 Feb 5) 27, 10.1038/s41392-024-01735-1. PMC10838959 38311623

[13] Zhu X, Badawi M, Pomeroy S, Sutaria DS, Xie Z, Baek A, , Comprehensive toxicity and immunogenicity studies reveal minimal effects in mice following sustained dosing of extracellular vesicles derived from HEK293T cells, J. Extracell. Vesicles 6 (1) (2017 Jun 6) 1324730, 10.1080/20013078.2017.1324730. 28717420 PMC5505007

[14] Kumeda N, Ogawa Y, Akimoto Y, Kawakami H, Tsujimoto M, Yanoshita R, Characterization of membrane integrity and morphological stability of Human salivary exosomes, Biol. Pharm. Bull. 40 (8) (2017) 1183–1191, 10.1248/bpb.b16-00891. 28768999

[15] Xin H, Li Y, Cui Y, Yang JJ, Zhang ZG, Chopp M, Systemic administration of exosomes released from mesenchymal stromal cells promote functional recovery and neurovascular plasticity after stroke in rats, J. Cereb. Blood Flow Metab. 33 (11) (2013 Nov) 1711–1715, 10.1038/jcbfm.2013.152. 23963371 PMC3824189

[16] Zhang Y, Chopp M, Meng Y, Katakowski M, Xin H, Mahmood A, , Effect of exosomes derived from multipluripotent mesenchymal stromal cells on functional recovery and neurovascular plasticity in rats after traumatic brain injury, J. Neurosurg. 122 (4) (2015 Apr) 856–867, 10.3171/2014.11.JNS14770. 25594326 PMC4382456

[17] Acharya MM, Martirosian V, Christie LA, Limoli CL, Long-term cognitive effects of human stem cell transplantation in the irradiated brain, Int. J. Radiat. Biol. 90 (9) (2014 Sep) 816–820, 10.3109/09553002.2014.927934. 24882389 PMC5027837

[18] Acharya MM, Christie LA, Hazel TG, Johe KK, Limoli CL, Transplantation of human fetal-derived neural stem cells improves cognitive function following cranial irradiation, Cell Transplant. 23 (10) (2014) 1255–1266, 10.3727/096368913X670200. 23866792 PMC3895108

[19] Acharya MM, Rosi S, Jopson T, Limoli CL, Human neural stem cell transplantation provides long-term restoration of neuronal plasticity in the irradiated Hippocampus, Cell Transplant. 24 (4) (2015) 691–702, 10.3727/096368914X684600. 25289634 PMC5027839

[20] Chopp M, Zhang ZG, Emerging potential of exosomes and noncoding microRNAs for the treatment of neurological injury/diseases, Expert Opin. Emerg. Drugs 20 (4) (2015) 523–526, 10.1517/14728214.2015.1061993. 26135408 PMC4878696

[21] Zhang Y, Chopp M, Zhang ZG, Katakowski M, Xin H, Qu C, , Systemic administration of cell-free exosomes generated by human bone marrow derived mesenchymal stem cells cultured under 2D and 3D conditions improves functional recovery in rats after traumatic brain injury, Neurochem. Int. 111 (2017 Dec) 69–81, 10.1016/j.neuint.2016.08.003. 27539657 PMC5311054

[22] Zhang ZG, Chopp M, Exosomes in stroke pathogenesis and therapy, J. Clin. Investig. 126 (4) (2016 Apr 1) 1190–1197, 10.1172/JCI81133. 27035810 PMC4811130

[23] Zhang Y, Chopp M, Liu XS, Katakowski M, Wang X, Tian X, , Exosomes derived from mesenchymal stromal cells promote axonal growth of cortical neurons, Mol. Neurobiol. 54 (4) (2017 May) 2659–2673, 10.1007/s12035-016-9851-0. 26993303 PMC5028236

[24] Apodaca LA, Baddour AAD, Garcia C, Alikhani L, Giedzinski E, Ru N, , Human neural stem cell-derived extracellular vesicles mitigate hallmarks of Alzheimer's disease, Alzheimers Res. Ther. 13 (1) (2021 Mar 6) 57, 10.1186/s13195-021-00791-x. 33676561 PMC7937214

[25] Karnas E, Dudek P, Zuba-Surma EK, Stem cell-derived extracellular vesicles as new tools in regenerative medicine - immunomodulatory role and future perspectives, Front. Immunol. 14 (2023 Jan 24) 1120175, 10.3389/fimmu.2023.1120175. 36761725 PMC9902918

[26] Baulch JE, Acharya MM, Allen BD, Ru N, Chmielewski NN, Martirosian V, , Cranial grafting of stem cell-derived microvesicles improves cognition and reduces neuropathology in the irradiated brain, Proc. Natl. Acad. Sci. U. S. A. 113 (17) (2016 Apr 26) 4836–4841, 10.1073/pnas.1521668113. 27044087 PMC4855546

[27] Smith SM, Giedzinski E, Angulo MC, Lui T, Lu C, Park AL, , Functional equivalence of stem cell and stem cell-derived extracellular vesicle transplantation to repair the irradiated brain, Stem Cells Transl. Med. 9 (1) (2020 Jan) 93–105, 10.1002/sctm.18-0227. 31568685 PMC6954724

[28] Uchida N, Buck DW, He D, Reitsma MJ, Masek M, Phan TV, , Direct isolation of human central nervous system stem cells, Proc. Natl. Acad. Sci. U. S. A. 97 (26) (2000 Dec 19) 14720–14725, 10.1073/pnas.97.26.14720. 11121071 PMC18985

[29] Cummings BJ, Uchida N, Tamaki SJ, Salazar DL, Hooshmand M, Summers R, , Human neural stem cells differentiate and promote locomotor recovery in spinal cord-injured mice, Proc. Natl. Acad. Sci. U. S. A. 102 (39) (2005 Sep 27) 14069–14074, 10.1073/pnas.0507063102. 16172374 PMC1216836

[30] Haus DL, Nguyen HX, Gold EM, Kamei N, Perez H, Moore HD, , CD133-enriched Xeno-Free human embryonic-derived neural stem cells expand rapidly in culture and do not form teratomas in immunodeficient mice, Stem Cell Res. 13 (2) (2014 Sep) 214–226, 10.1016/j.scr.2014.06.008. 25082219 PMC5675021

[31] Haus DL, López-Velázquez L, Gold EM, Cunningham KM, Perez H, Anderson AJ, , Transplantation of human neural stem cells restores cognition in an immunodeficient rodent model of traumatic brain injury, Exp. Neurol. 281 (2016 Jul 1) 1–16, 10.1016/j.expneurol.2016.04.008. 27079998

[32] Badner A, Reinhardt EK, Nguyen TV, Midani N, Marshall AT, Lepe CA, , Freshly thawed cryobanked human neural stem cells engraft within endogenous neurogenic niches and restore cognitive function after chronic Traumatic Brain injury, J. Neurotrauma 38 (19) (2021 Oct 1) 2731–2746, 10.1089/neu.2021.0045. 34130484

[33] Beretta S, Cunningham KM, Haus DL, Gold EM, Perez H, López-Velázquez L, , Effects of human ES-Derived neural stem cell transplantation and kindling in a rat model of traumatic brain injury, Cell Transplant. 26 (7) (2017 Jul) 1247–1261, 10.1177/0963689717714107. 28933218 PMC5657732

[34] Théry C, Amigorena S, Raposo G, Clayton A, Isolation and characterization of exosomes from cell culture supernatants and biological fluids, Curr. Protoc. Cell Biol. 30 (1) (2006 Mar), 10.1002/0471143030.cb0322s30, 3.22.1-3.22.29. 18228490

[35] Warburton EC, Brown MW, Neural circuitry for rat recognition memory. SI: Object Recognition Memory in Rats and Mice 285, Behav Brain Res, 2015 May 15, pp. 131–139, 10.1016/j.bbr.2014.09.050. PMC4383363 25315129

[36] Plas SL, Tuna T, Bayer H, Juliano VAL, Sweck SO, Arellano Perez AD, , Neural circuits for the adaptive regulation of fear and extinction memory, Front. Behav. Neurosci. (2024 Feb 2) 18, 10.3389/fnbeh.2024.1352797. PMC10869525 38370858

[37] Doyle LM, Wang MZ, Overview of extracellular vesicles, their origin, composition, purpose, and methods for exosome isolation and analysis, Cells 8 (7) (2019 Jul 15) 727, 10.3390/cells8070727. 31311206 PMC6678302

[38] Zhao Y, Huang Y, Cao Y, Yang J, Astrocyte-Mediated neuroinflammation in neurological conditions, Biomolecules 14 (10) (2024 Sep 25) 1204, 10.3390/biom14101204. 39456137 PMC11505625

[39] Augusto-Oliveira M, de P G. Arrifano C.G. Leal-Nazaré, Chaves-Filho A, Santos-Sacramento L, Lopes-Araujo A, , Morphological diversity of microglia: implications for learning, environmental adaptation, ageing, sex differences and neuropathology, Neurosci. Biobehav. Rev. 172 (2025 May 1) 106091, 10.1016/j.neubiorev.2025.106091. 40049541

[40] Acharya MM, Christie LA, Lan ML, Giedzinski E, Fike JR, Rosi S, , Human neural stem cell transplantation ameliorates radiation-induced cognitive dysfunction, Cancer Res. 71 (14) (2011 Jul 15) 4834–4845, 10.1158/0008-5472.CAN-11-0027. 21757460 PMC3517293

[41] Acharya MM, Martirosian V, Christie LA, Riparip L, Strnadel J, Parihar VK, , Defining the optimal window for cranial transplantation of human induced pluripotent stem cell-derived cells to ameliorate radiation-induced cognitive impairment, Stem Cells Transl. Med. 4 (1) (2015 Jan) 74–83, 10.5966/sctm.2014-0063. 25391646 PMC4275007

[42] Acharya MM, Martirosian V, Chmielewski NN, Hanna N, Tran KK, Liao AC, , Stem cell transplantation reverses chemotherapy-induced cognitive dysfunction, Cancer Res. 75 (4) (2015 Feb 15) 676–686, 10.1158/0008-5472.CAN-14-2237. 25687405 PMC4332567

[43] Acharya MM, Christie LA, Lan ML, Limoli CL, Comparing the functional consequences of human stem cell transplantation in the irradiated rat brain, Cell Transplant. 22 (1) (2013) 55–64, 10.3727/096368912X640565. 22546529 PMC3601190

[44] Acharya MM, Christie LA, Lan ML, Donovan PJ, Cotman CW, Fike JR, , Rescue of radiation-induced cognitive impairment through cranial transplantation of human embryonic stem cells, Proc. Natl. Acad. Sci. U. S. A. 106 (45) (2009 Nov 10) 19150–19155, 10.1073/pnas.0909293106. 19901336 PMC2776427

[45] Leavitt RJ, Acharya MM, Baulch JE, Limoli CL, Extracellular vesicle-derived miR-124 resolves radiation-induced brain injury, Cancer Res. 80 (19) (2020 Oct 1) 4266–4277, 10.1158/0008-5472.CAN-20-1599. 32816912 PMC7541572

[46] Leavitt RJ, Limoli CL, Baulch JE, miRNA-based therapeutic potential of stem cell-derived extracellular vesicles: a safe cell-free treatment to ameliorate radiation-induced brain injury, Int. J. Radiat. Biol. 95 (4) (2019 Apr) 427–435, 10.1080/09553002.2018.1522012. 30252569 PMC6433537

[47] Ioannides P, Giedzinski E, Limoli CL, Evaluating different routes of extracellular vesicle administration for cranial therapies, J Cancer Metastasis Treat 6 (15) (2020), https://doi.org/10.20517/2394-4722.2020.22 doi:10.20517/2394-4722.2020.22 PMC8281946 34277952

[48] Stupp R, Hegi ME, Gilbert MR, Chakravarti A, Chemoradiotherapy in malignant glioma: standard of care and future directions, J Clin Oncol Off J Am Soc Clin Oncol 25 (26) (2007 Sep 10) 4127–4136, 10.1200/JCO.2007.11.8554. 17827463

[49] Elsharkasy OM, de Voogt WS, Tognoli ML, van der Werff L, Gitz-Francois JJ, Seinen CW, , Integrin beta 1 and fibronectin mediate extracellular vesicle uptake and functional RNA delivery, J. Biol. Chem. 301 (3) (2025 Feb 11) 108305, 10.1016/j.jbc.2025.108305. 39947471 PMC12020920

[50] Théry C, Witwer KW, Aikawa E, Alcaraz MJ, Anderson JD, Andriantsitohaina R, , Minimal information for studies of extracellular vesicles 2018 (MISEV2018): a position statement of the International Society for Extracellular Vesicles and update of the MISEV2014 guidelines, J. Extracell. Vesicles 7 (1) (2018 Nov 23) 1535750, 10.1080/20013078.2018.1535750. 30637094 PMC6322352

[51] Kugeratski FG, Hodge K, Lilla S, McAndrews KM, Zhou X, Hwang RF, , Quantitative Proteomics Identifies the Core Proteome of Exosomes with Syntenin-1 as the highest abundant protein and a Putative Universal Biomarker, Nat. Cell Biol. 23 (6) (2021 Jun) 631–641, 10.1038/s41556-021-00693-y. 34108659 PMC9290189

[52] El-Khatib SM, Vagadia AR, Le ACD, Baulch JE, Ng DQ, Du M, , BDNF augmentation reverses cranial radiation therapy-induced cognitive decline and neurodegenerative consequences, Acta Neuropathol. Commun. 12 (1) (2024 Dec 18) 190, 10.1186/s40478-024-01906-9. 39696694 PMC11654382

[53] Usmani MT, Krattli RP, El-Khatib SM, Le ACD, Smith SM, Baulch JE, , BDNF augmentation using riluzole reverses doxorubicin-induced decline in cognitive function and neurogenesis, Neurother J Am Soc Exp Neurother 20 (3) (2023 Apr) 838–852, 10.1007/s13311-022-01339-z. PMC10275819 36720792

[54] Markarian M, Krattli RP, Baddour JD, Alikhani L, Giedzinski E, Usmani MT, , Glia-Selective deletion of complement C1q prevents radiation-induced cognitive deficits and neuroinflammation, Cancer Res. 81 (7) (2021 Apr 1) 1732–1744, 10.1158/0008-5472.CAN-20-2565. 33323383 PMC8137520

[55] Allen BD, Apodaca LA, Syage AR, Markarian M, Baddour AAD, Minasyan H, , Attenuation of neuroinflammation reverses Adriamycin-induced cognitive impairments, Acta Neuropathol. Commun. 7 (2019 Nov 21) 186, 10.1186/s40478-019-0838-8. 31753024 PMC6868786

[56] Krattli RP, Do AH, El-Khatib SM, Alikhani L, Markarian M, Vagadia AR, , C5aR1 inhibition alleviates cranial radiation-induced cognitive decline, Cancer Res. 86 (1) (2026 Jan 2) 255–272, 10.1158/0008-5472.CAN-24-4869. 41085542 PMC12818912

[57] Milad MR, Quirk GJ, Fear extinction as a model for translational neuroscience: ten years of progress, Annu. Rev. Psychol. 63 (2012) 129–151, 10.1146/annurev.psych.121208.131631. 22129456 PMC4942586

[58] Barker GRI, Warburton EC, When is the hippocampus involved in recognition memory? J Neurosci Off J Soc Neurosci 31 (29) (2011 Jul 20) 10721–10731, 10.1523/JNEUROSCI.6413-10.2011. PMC6622630 21775615

[59] Barker GRI, Bird F, Alexander V, Warburton EC, Recognition memory for objects, place, and temporal order: a disconnection analysis of the role of the medial prefrontal cortex and perirhinal cortex, J Neurosci Off J Soc Neurosci 27 (11) (2007 Mar 14) 2948–2957, 10.1523/JNEUROSCI.5289-06.2007. PMC6672574 17360918

[60] Makale MT, McDonald CR, Hattangadi-Gluth JA, Kesari S, Mechanisms of radiotherapy-associated cognitive disability in patients with brain tumours, Nat. Rev. Neurol. 13 (1) (2017 Jan) 52–64, 10.1038/nrneurol.2016.185. 27982041 PMC5805381

[61] Greene-Schloesser D, Moore E, Robbins ME, Molecular pathways: radiation-induced cognitive impairment, Clin Cancer Res Off J Am Assoc Cancer Res 19 (9) (2013 May 1) 2294–2300, 10.1158/1078-0432.CCR-11-2903. PMC3642233 23388505

[62] Greene-Schloesser D, Robbins ME, Radiation-induced cognitive impairment–from bench to bedside, Neuro Oncol. 14 (Suppl 4) (2012 Sep), 10.1093/neuonc/nos196. Suppl 4):iv37–44 23095829 PMC3480242

[63] Acevedo-Vergara K, Perez-Florez M, Ramirez A, Torres-Bayona S, Dau A, Salva S, , Cognitive deficits in adult patients with high-grade glioma: a systematic review, Clin. Neurol. Neurosurg. 219 (2022 Aug 1) 107296, 10.1016/j.clineuro.2022.107296. 35662053

[64] Cómitre-Mariano B, Vellila-Alonso G, Segura-Collar B, Mondéjar-Ruescas L, Sepulveda JM, Gargini R, Sentinels of neuroinflammation: the crucial role of myeloid cells in the pathogenesis of gliomas and neurodegenerative diseases, J. Neuroinflammation 21 (2024 Nov 22) 304, 10.1186/s12974-024-03298-y. 39578808 PMC11583668

[65] Liao Y, Zheng Y, Zhang R, Chen X, Huang J, Liu J, , Regulatory roles of transcription factors T-bet and Eomes in group 1 ILCs, Int. Immunopharmacol. 143 (Pt 1) (2024 Dec 25) 113229, 10.1016/j.intimp.2024.113229. 39357208

[66] Won SY, Kwon S, Jeong HS, Chung KW, Choi BO, Chang JW, , Fibulin 5, a human Wharton's jelly-derived mesenchymal stem cells-secreted paracrine factor, attenuates peripheral nervous system myelination defects through the Integrin-RAC1 signaling axis, Stem Cells Dayt Ohio 38 (12) (2020 Oct 27) 1578–1593, 10.1002/stem.3287. PMC7756588 33107705

[67] Sur I, Zhao W, Zhang J, Kling Pilström M, Webb AT, Cheng H, , Shared requirement for MYC upstream super-enhancer region in tissue regeneration and cancer, Life Sci. Alliance 8 (6) (2025 Jun) e202403090, 10.26508/lsa.202403090. 40180576 PMC11969384

[68] Shaheen ZR, Christmann BS, Stafford JD, Moran JM, Buller RML, Corbett JA, CCR5 is a required signaling receptor for macrophage expression of inflammatory genes in response to viral double-stranded RNA, Am. J. Physiol. Regul. Integr. Comp. Physiol. 316 (5) (2019 May 1) R525–R534, 10.1152/ajpregu.00019.2019. 30811246 PMC6589596

[69] Wang L, Jiang J, Chen Y, Jia Q, Chu Q, The roles of CC chemokines in response to radiation, Radiat Oncol Lond Engl 17 (1) (2022 Apr 1) 63, 10.1186/s13014-022-02038-x. PMC8974090 35365161

[70] Munshi A, Ramesh R, Mitogen-activated protein kinases and their role in radiation response, Genes Cancer 4 (9–10) (2013 Sep) 401–408, 10.1177/1947601913485414. 24349638 PMC3863336

[71] McKenna WG, Muschel RJ, Gupta AK, Hahn SM, Bernhard EJ, The RAS signal transduction pathway and its role in radiation sensitivity, Oncogene 22 (37) (2003 Sep 1) 5866–5875, 10.1038/sj.onc.1206699. 12947393

[72] Nam SY, Seo HH, Park HS, An S, Kim JY, Yang KH, , Phosphorylation of CLK2 at serine 34 and threonine 127 by AKT controls cell survival after ionizing radiation, J. Biol. Chem. 285 (41) (2010 Oct 8) 31157–31163, 10.1074/jbc.M110.122044. 20682768 PMC2951189

[73] Laukoter S, Beattie R, Pauler FM, Amberg N, Nakayama KI, Hippenmeyer S, Imprinted Cdkn1c genomic locus cell-autonomously promotes cell survival in cerebral cortex development, Nat. Commun. 11 (1) (2020 Jan 10) 195, 10.1038/s41467-019-14077-2. 31924768 PMC6954230

[74] Huang B, Lang X, Li X, The role of IL-6/JAK2/STAT3 signaling pathway in cancers, Front. Oncol. 12 (2022) 1023177, 10.3389/fonc.2022.1023177. 36591515 PMC9800921

[75] Ji L, Xu S, Luo H, Zeng F, Insights from DOCK2 in cell function and pathophysiology, Front. Mol. Biosci. 9 (2022) 997659, 10.3389/fmolb.2022.997659. 36250020 PMC9559381

[76] Savant S, La Porta S, Budnik A, Busch K, Hu J, Tisch N, , The orphan receptor Tie1 controls angiogenesis and vascular remodeling by differentially regulating Tie 2 in tip and stalk cells, Cell Rep. 12 (11) (2015 Sep 22) 1761–1773, 10.1016/j.celrep.2015.08.024. 26344773 PMC6309948

[77] Chen Z, Yu M, Yan J, Guo L, Zhang B, Liu S, , PNOC expressed by B cells in cholangiocarcinoma was survival related and LAIR2 could be a T cell exhaustion biomarker in tumor microenvironment: characterization of immune microenvironment combining single-cell and bulk sequencing technology, Front. Immunol. 12 (2021) 647209, 10.3389/fimmu.2021.647209. 33841428 PMC8024580

[78] Almasan A, Ashkenazi A, Apo2L/TRAIL: apoptosis signaling, biology, and potential for cancer therapy, Cytokine Growth Factor Rev. 14 (3–4) (2003) 337–348, 10.1016/s1359-6101(03)00029-7. 12787570

[79] Jaworski J, Kalita K, Knapska E, c-Fos and neuronal plasticity: the aftermath of Kaczmarek's theory, Acta Neurobiol. Exp. 78 (4) (2018) 287–296. 30624427

[80] Hao B, Dong H, Xiong R, Song C, Xu C, Li N, , Identification of SLC2A1 as a predictive biomarker for survival and response to immunotherapy in lung squamous cell carcinoma, Comput. Biol. Med. 171 (2024 Mar) 108183, 10.1016/j.compbiomed.2024.108183. 38422959

[81] Liu Z, Lei X, Li X, Cai JM, Gao F, Yang YY, Toll-like receptors and radiation protection, Eur. Rev. Med. Pharmacol. Sci. 22 (1) (2018 Jan) 31–39, 10.26355/eurrev_201801_14097. 29364499

[82] Maliah A, Santana-Magal N, Parikh S, Gordon S, Reshef K, Sade Y, , Crosslinking of Ly6a metabolically reprograms CD8 T cells for cancer immunotherapy, Nat. Commun. 15 (1) (2024 Sep 27) 8354, 10.1038/s41467-024-52079-x. 39333093 PMC11437002

[83] Yang X, Duan Y, Zhao Z, Contemplating the prognostic and therapeutic potential of CD19: a comprehensive analysis across diverse cancer types, Am. J. Transl. Res. 16 (11) (2024) 6365–6383, 10.62347/KJGS9928. 39678610 PMC11645609

[84] Epstein I, Finkbeiner S, The arc of cognition: signaling cascades regulating Arc and implications for cognitive function and disease, Semin. Cell Dev. Biol. 77 (2018 May) 63–72, 10.1016/j.semcdb.2017.09.023. 29559111 PMC5865643

[85] den Hoed J, Sollis E, Venselaar H, Estruch SB, Deriziotis P, Fisher SE, Functional characterization of TBR1 variants in neurodevelopmental disorder, Sci. Rep. 8 (1) (2018 Sep 24) 14279, 10.1038/s41598-018-32053-6. 30250039 PMC6155134

[86] Hermann DM, Peruzzotti-Jametti L, Giebel B, Pluchino S, Extracellular vesicles set the stage for brain plasticity and recovery by multimodal signalling, Brain J Neurol 147 (2) (2024 Feb 1) 372–389, 10.1093/brain/awad332. PMC10834259 37768167

[87] Fallahi S, Zangbar HS, Farajdokht F, Rahbarghazi R, Mohaddes G, Ghiasi F, Exosomes as a therapeutic tool to promote neurorestoration and cognitive function in neurological conditions: achieve two ends with a single effort, CNS Neurosci. Ther. 30 (5) (2024 May) e14752, 10.1111/cns.14752. 38775149 PMC11110007

[88] Roy C, Avril S, Legendre C, Lelièvre B, Vellenriter H, Boni S, , A role for ceruloplasmin in the control of human glioblastoma cell responses to radiation, BMC Cancer 22 (1) (2022 Aug 2) 843, 10.1186/s12885-022-09808-6. 35918659 PMC9347084

[89] Sharanek A, Burban A, Laaper M, Heckel E, Joyal JS, Soleimani VD, , OSMR controls glioma stem cell respiration and confers resistance of glioblastoma to ionizing radiation, Nat. Commun. 11 (1) (2020 Aug 17) 4116, 10.1038/s41467-020-17885-z. 32807793 PMC7431428

