# Supplementary material for: Human neural stem cell-derived extracellular vesicles improve cognitive function following glioma chemoradiation therapy
Source: Cancer Lett. Author manuscript; Available in PMC 2026 Aug 10. (PMC13340850; doi:10.1016/j.canlet.2026.218564)
Supplement: Supplementary file 1 [file NIHMS2185260-supplement-Supplementary_Data.pdf]

Suppl. Fig. S1

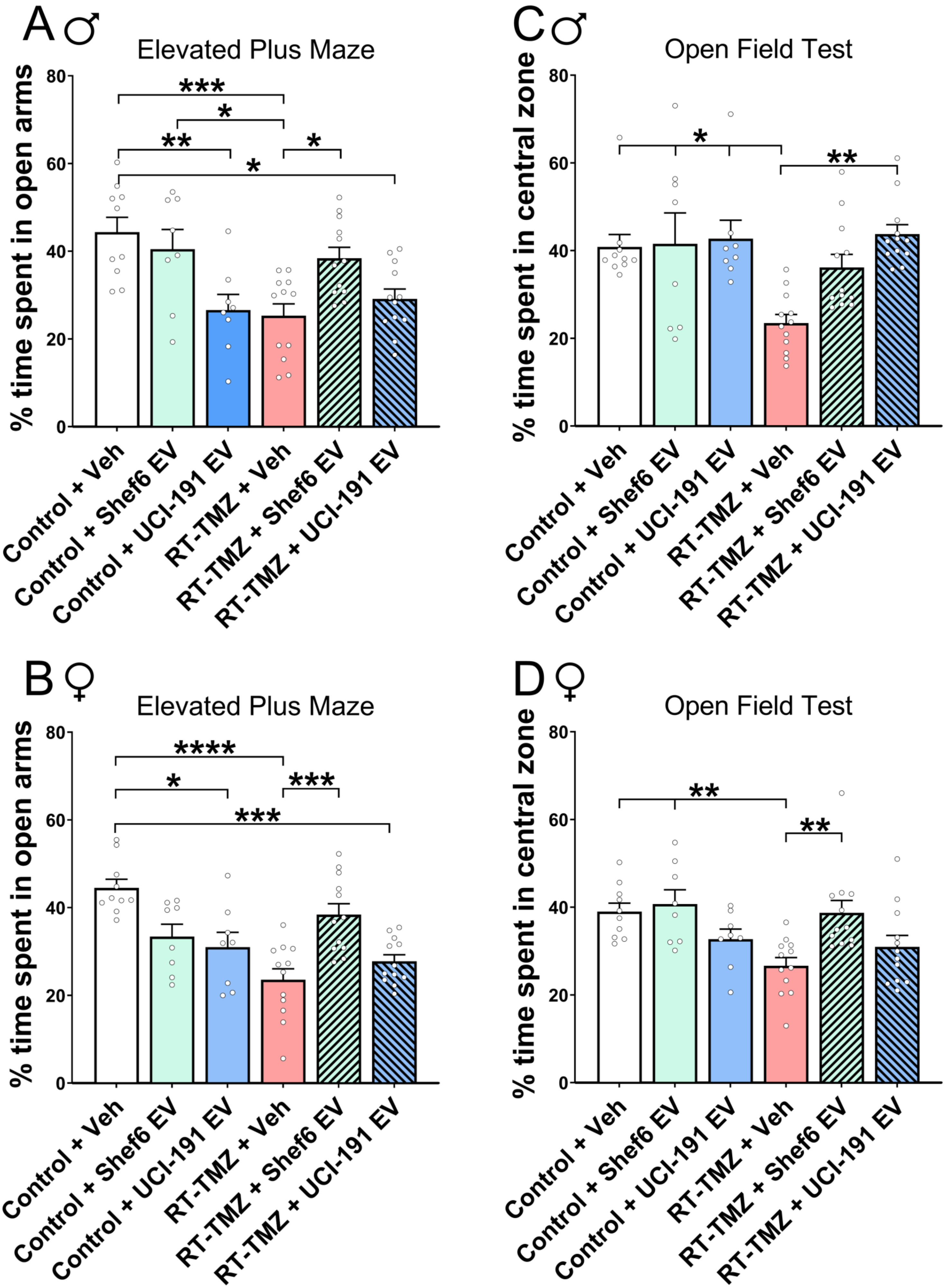
A ♂
Elevated Plus Maze
% time spent in open arms
0
20
40
60
80
***
*
**
*
*
Control + Veh
Control + Shef6 EV
Control + UCI-191 EV
RT-TMZ + Veh
RT-TMZ + Shef6 EV
RT-TMZ + UCI-191 EV
C ♂
Open Field Test
% time spent in central zone
0
20
40
60
80
*
**
Control + Veh
Control + Shef6 EV
Control + UCI-191 EV
RT-TMZ + Veh
RT-TMZ + Shef6 EV
RT-TMZ + UCI-191 EV
B ♀
Elevated Plus Maze
% time spent in open arms
0
20
40
60
80
****
*
***
***
Control + Veh
Control + Shef6 EV
Control + UCI-191 EV
RT-TMZ + Veh
RT-TMZ + Shef6 EV
RT-TMZ + UCI-191 EV
D ♀
Open Field Test
% time spent in central zone
0
20
40
60
80
**
**
Control + Veh
Control + Shef6 EV
Control + UCI-191 EV
RT-TMZ + Veh
RT-TMZ + Shef6 EV
RT-TMZ + UCI-191 EV

Supplemental Figure S1.

Effects of combined cranial radiotherapy and chemotherapy, or hNSC-derived EVs, on anxiety-like behavior.

(**A**-**D**) Analysis of percent time spent in the open arms of elevated plus maze (EPM) and the percentage time spent in the central zone of an open field arena in the open field test (OFT) is shown for the male (**A** and **B**) and female (**C** and **D**) mice after treatment with cranial radiation therapy (RT) and temozolomide (TMZ) followed by RO injections of either vehicle (RT-TMZ + Veh) or hNSC-EVs (RT-TMZ + Shef6 EV; RT-TMZ + UCI-191 EV). Combined RT and TMZ exposure (RT-TMZ + Veh) significantly reduced percentage times spent in either open arms (**A, C**; EPM) or the central zone (**C, D**; OFT) by male and female mice.

Data are represented by mean ± SEM (N = 5-12 animals/group). *P* values were derived from two-way ANOVA and Bonferroni's multiple comparisons tests. *, P ≤ 0.05; **, P ≤ 0.01; *** P ≤ 0.001; ****, P ≤ 0.0001.

Suppl. Fig. S2

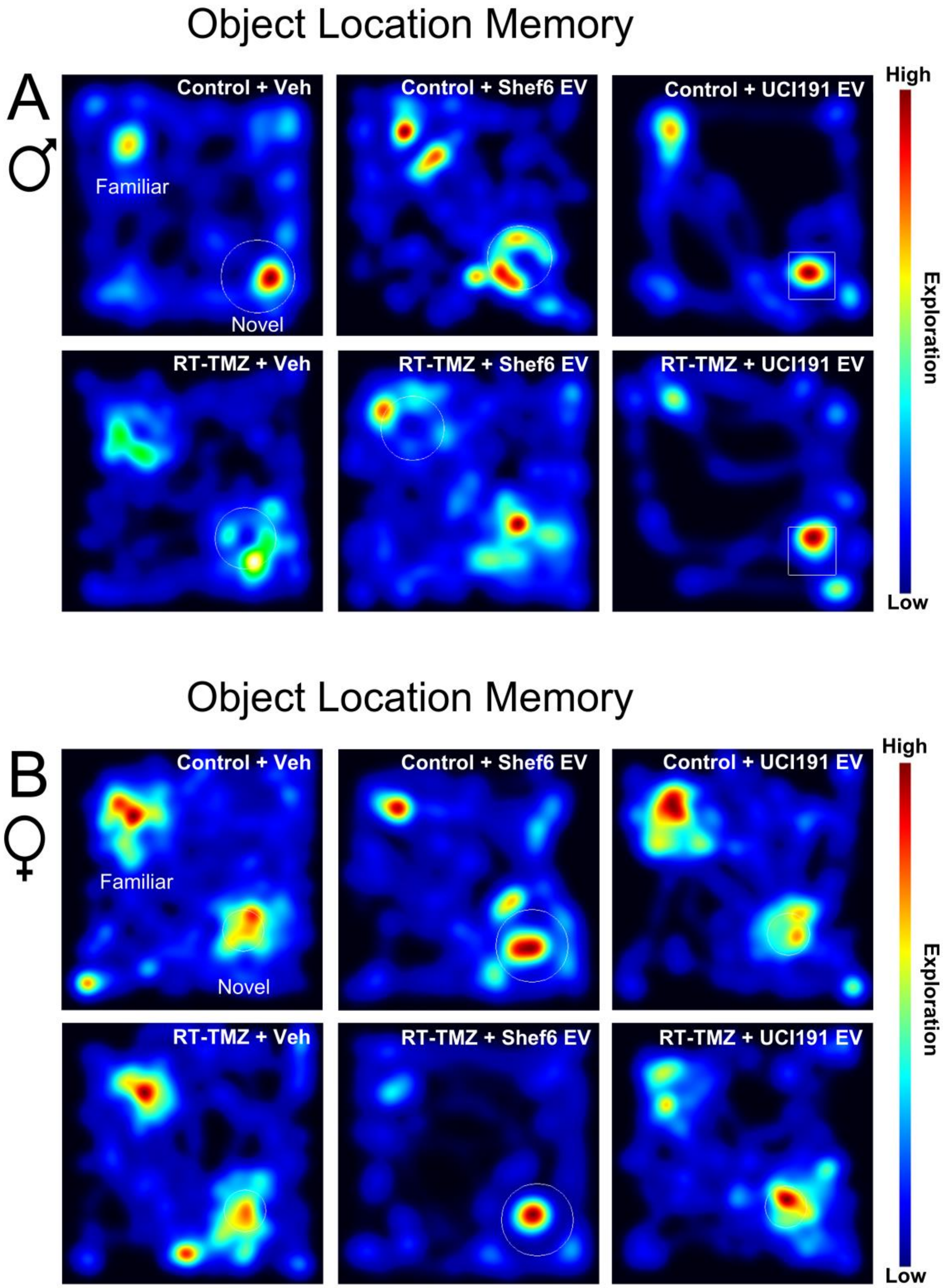
Object Location Memory
A
♂
Control + Veh
Familiar
Novel
Control + Shef6 EV
Control + UCI191 EV
RT-TMZ + Veh
RT-TMZ + Shef6 EV
RT-TMZ + UCI191 EV
High
Exploration
Low
Object Location Memory
B
♀
Control + Veh
Familiar
Novel
Control + Shef6 EV
Control + UCI191 EV
RT-TMZ + Veh
RT-TMZ + Shef6 EV
RT-TMZ + UCI191 EV
High
Exploration
Low

Supplemental Figure S2.

Representative heatmaps of exploration in Object Location Memory.

(**A**-**B**) Representative heatmaps depicting exploration throughout object location memory (OLM) test arena for male (**A**) and female (**B**) mice for the data presented in **Figures 2C** and **2E**.

Suppl. Fig. S3

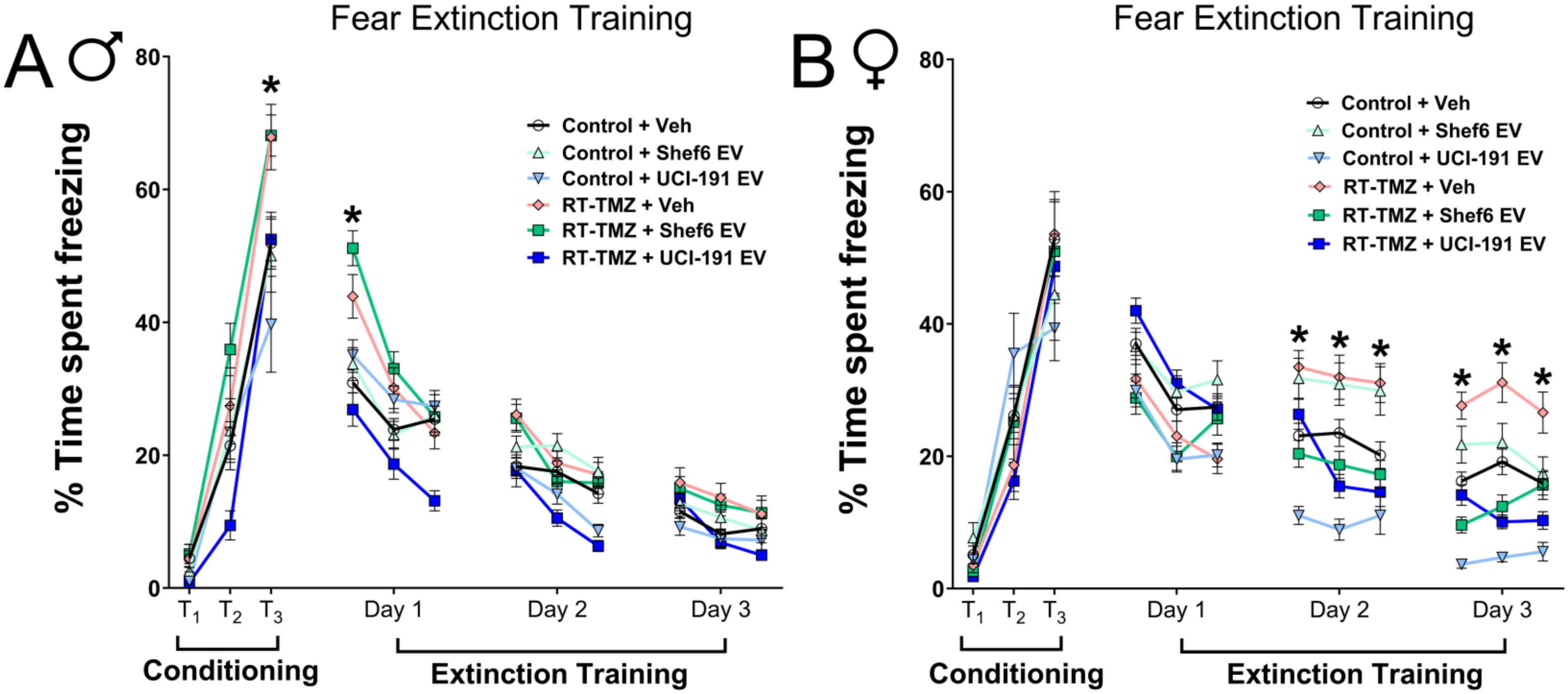

A ♂
Fear Extinction Training
% Time spent freezing
80
60
40
20
0
*
*
Control + Veh
Control + Shef6 EV
Control + UCI-191 EV
RT-TMZ + Veh
RT-TMZ + Shef6 EV
RT-TMZ + UCI-191 EV
T1
T2
T3
Day 1
Day 2
Day 3
Conditioning
Extinction Training
B ♀
Fear Extinction Training
% Time spent freezing
80
60
40
20
0
Control + Veh
Control + Shef6 EV
Control + UCI-191 EV
RT-TMZ + Veh
RT-TMZ + Shef6 EV
RT-TMZ + UCI-191 EV
* * *
* * *
T1
T2
T3
Day 1
Day 2
Day 3
Conditioning
Extinction Training

Supplemental Figure S3.

Effects of combined cranial radiotherapy and chemotherapy, or hNSC-derived EVs, on fear conditioning and extinction.

(**A**-**B**) Graphical depiction of sequential fear extinction (*Days 1-3*) in male (**A**) and female (**B**) mice after the initial fear conditioning phase (*T1-3*). Successful extinction of the conditioned fear response is evidenced by a decrease in the percentage of time spent freezing (**A** and **B**). Analysis of the percentage of time spent freezing on the final Test Day of fear extinction (FE) is depicted in **Figures 2H** and **2I**.

Data are represented by mean ± SEM (N = 5-12 animals/group). *P* values were derived from two-way ANOVA and Bonferroni's multiple comparisons tests. *, $P \leq 0.05$ vs. Control + Veh group.

Suppl. Fig. S4

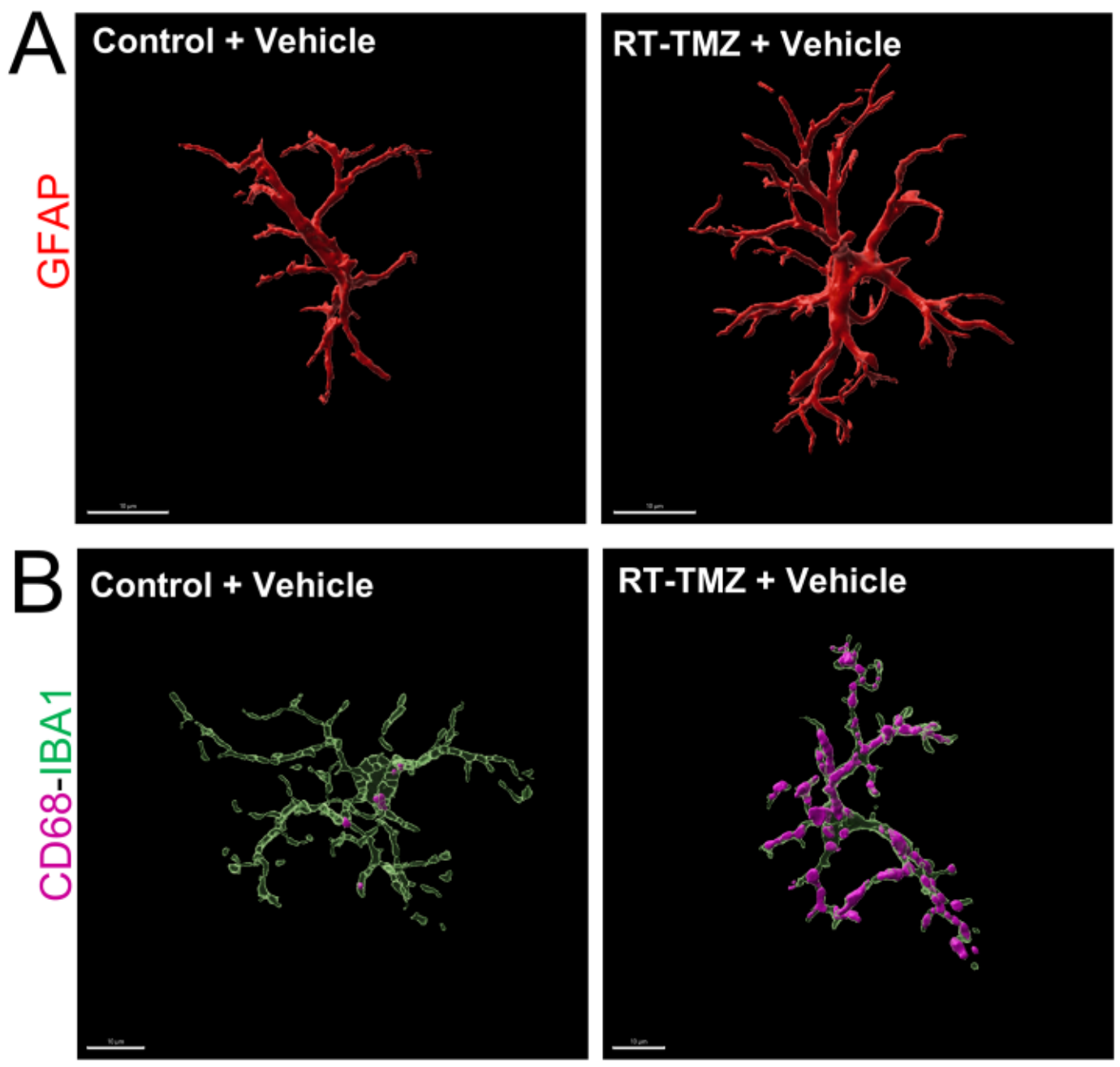
A
Control + Vehicle
RT-TMZ + Vehicle
GFAP
B
Control + Vehicle
RT-TMZ + Vehicle
CD68-IBA1

Supplemental Figure S4.

Neuroinflammation caused by combined cranial radiation and chemotherapy is prevented by hNSC-EVs.

(**A**-**B**) High resolution immunoreactive surface rendering of single cell images in the Control + Veh and RT-TMZ + Veh groups in female brains for GFAP+ astrocytes (**A**) and IBA1-CD68+ dual-labeled microglia (**B**).

Scale bars 10 µm (**A** and **B**).

Suppl. Fig. S5

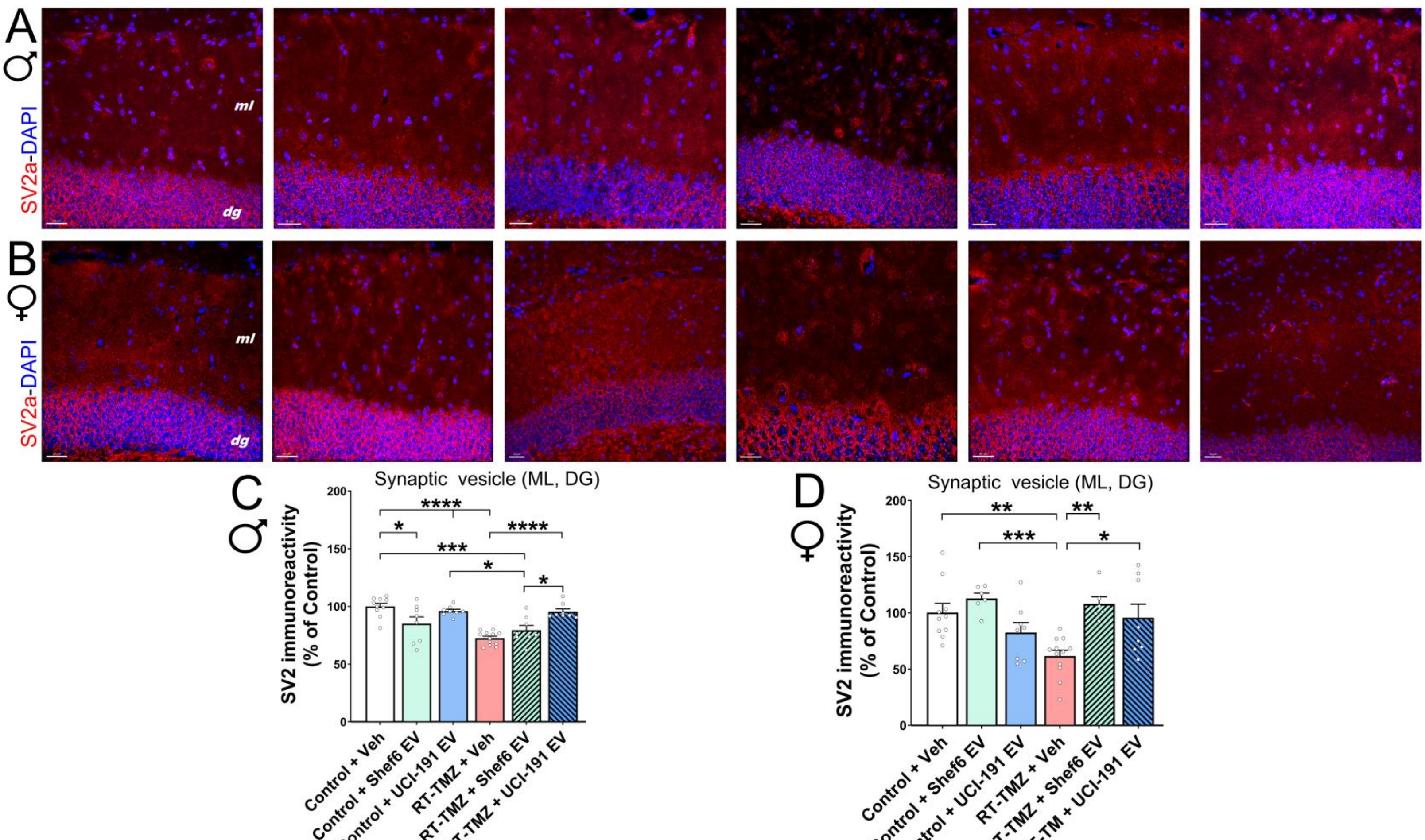

A
♂
SV2a-DAPI
ml
dg
B
♀
SV2a-DAPI
ml
dg
C
♂
Synaptic vesicle (ML, DG)
SV2 immunoreactivity (% of Control)
200
150
100
50
0
*
****
***
*
****
*
Control + Veh
Control + Shef6 EV
Control + UCI-191 EV
RT-TMZ + Veh
RT-TMZ + Shef6 EV
RT-TMZ + UCI-191 EV
D
♀
Synaptic vesicle (ML, DG)
SV2 immunoreactivity (% of Control)
200
150
100
50
0
**
**
***
*
Control + Veh
Control + Shef6 EV
Control + UCI-191 EV
RT-TMZ + Veh
RT-TMZ + Shef6 EV
RT-TM + UCI-191 EV

Supplemental Figure S5.

hNSC-EVs prevent the loss of SV2a following combined cranial radiation therapy and chemotherapy.

Fluorescent confocal micrographs and volumetric quantification of synaptic density in male (**A** and **C**) and female (**B** and **D**) mice.

(**A**-**D**) Immunofluorescence staining, confocal microscopy, and 3D algorithm-based quantification for synaptic vesicle glycoprotein 2A (SV2a; red) with DAPI nuclear counter stain (blue) in the hippocampal molecular layer (*ml*) and dentate gyrus (*dg*).

Scale bars, 30 μm (**A**-**B**). Data are represented by mean ± SEM (N = 6-12 animals/group). *P* values were calculated by one-way ANOVA and Tukey's multiple comparisons tests. *, $P \leq 0.05$; **, $P \leq 0.01$; *** $P \leq 0.001$; ****, $P \leq 0.0001$.

Suppl. Fig. S6

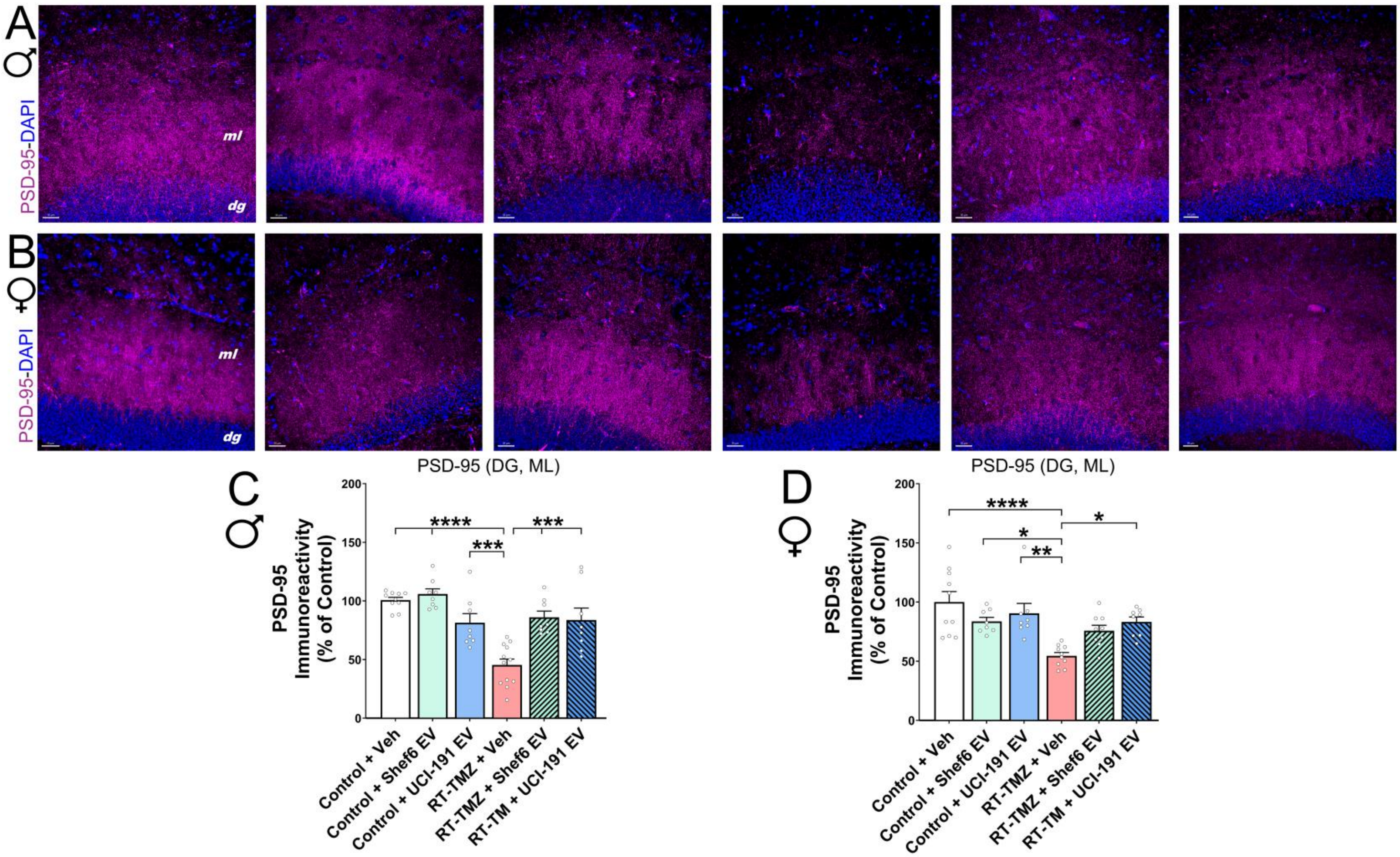

A
♂
PSD-95-DAPI
ml
dg
B
♀
PSD-95-DAPI
ml
dg
C
♂
PSD-95 (DG, ML)
PSD-95 Immunoreactivity (% of Control)
200
150
100
50
0
****
***
***
Control + Veh
Control + Shef6 EV
Control + UCI-191 EV
RT-TMZ + Veh
RT-TMZ + Shef6 EV
RT-TM + UCI-191 EV
D
♀
PSD-95 (DG, ML)
PSD-95 Immunoreactivity (% of Control)
200
150
100
50
0
****
*
**
*
Control + Veh
Control + Shef6 EV
Control + UCI-191 EV
RT-TMZ + Veh
RT-TMZ + Shef6 EV
RT-TM + UCI-191 EV

Supplemental Figure S6.

hNSC-EVs prevent the loss of PSD-95 following combined cranial radiation therapy and chemotherapy.

Fluorescent confocal micrographs and volumetric quantification of synaptic density in male (**A** and **C**) and female (**B** and **D**) mice.

(**A**-**D**) Representative confocal micrographs and analysis of postsynaptic density protein, PSD-95 (magenta) with DAPI nuclear counter staining in hippocampal molecular layer (*ml*) and dentate gyrus (*dg*).

Scale bars, 30 µm (**A**-**B**). Data are represented by mean ± SEM (N = 6-12 animals/group). *P* values were calculated by one-way ANOVA and Tukey's multiple comparisons tests. *, $P \leq 0.05$; **, $P \leq 0.01$; *** $P \leq 0.001$; ****, $P \leq 0.0001$.

Suppl. Fig. S7

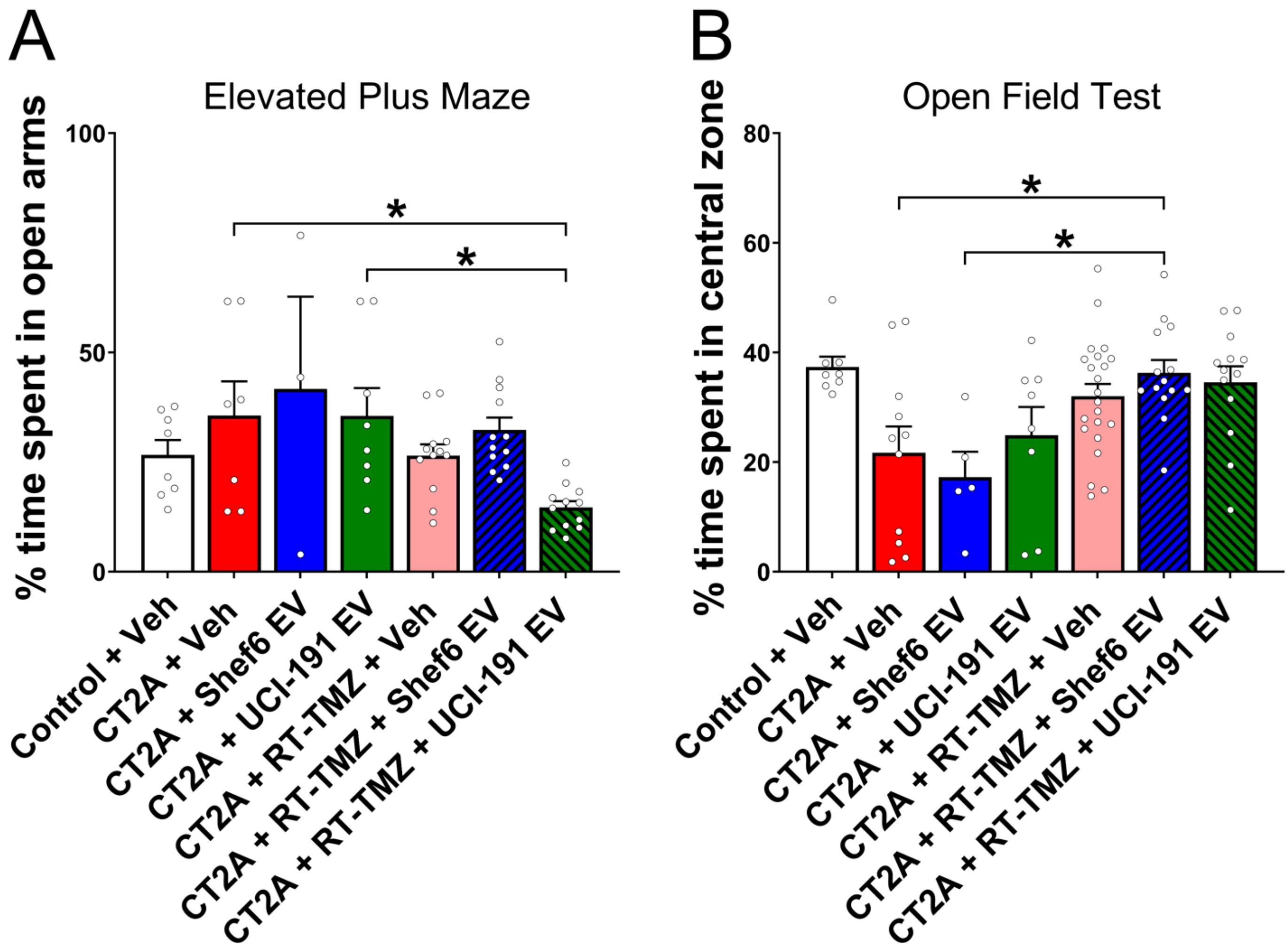

A
Elevated Plus Maze
% time spent in open arms
100
50
0
*
*
Control + Veh
CT2A + Veh
CT2A + Shef6 EV
CT2A + UCI-191 EV
CT2A + RT-TMZ + Veh
CT2A + RT-TMZ + Shef6 EV
CT2A + RT-TMZ + UCI-191 EV
B
Open Field Test
% time spent in central zone
80
60
40
20
0
*
*
Control + Veh
CT2A + Veh
CT2A + Shef6 EV
CT2A + UCI-191 EV
CT2A + RT-TMZ + Veh
CT2A + RT-TMZ + Shef6 EV
CT2A + RT-TMZ + UCI-191 EV

Supplemental Figure S7.

Effects of combined cranial radiotherapy and chemotherapy or hNSC-EVs, on anxiety-like behavior in glioma-bearing mice.

(**A**-**B**) Analysis of percent time spent in the open arms of the elevated plus maze (EPM, **A**) and the percentage time spent in the central zone of an open field arena in the open field test (OFT, **B**) in the control (Control + Veh) and glioma-bearing mice. After confirmation of CT2A-Luc^{+} cancer burden, mice were treated with cranial radiation therapy (RT) and chemotherapy followed by bilateral RO injections of hNSC-EVs (CT2A + RT-TMZ + Shef6 EV, CT2A + RT-TMZ + UCI-191 EV) or vehicle (CT2A + RT-TMZ + Veh; sterile PBS).

Data are represented by mean ± SEM (N = 5-12 animals/group). *P* values were derived from two-way ANOVA and Bonferroni's multiple comparisons tests. *, P ≤ 0.05; **, P ≤ 0.01; *** P ≤ 0.001; ****, P ≤ 0.0001.

Suppl. Fig. S8

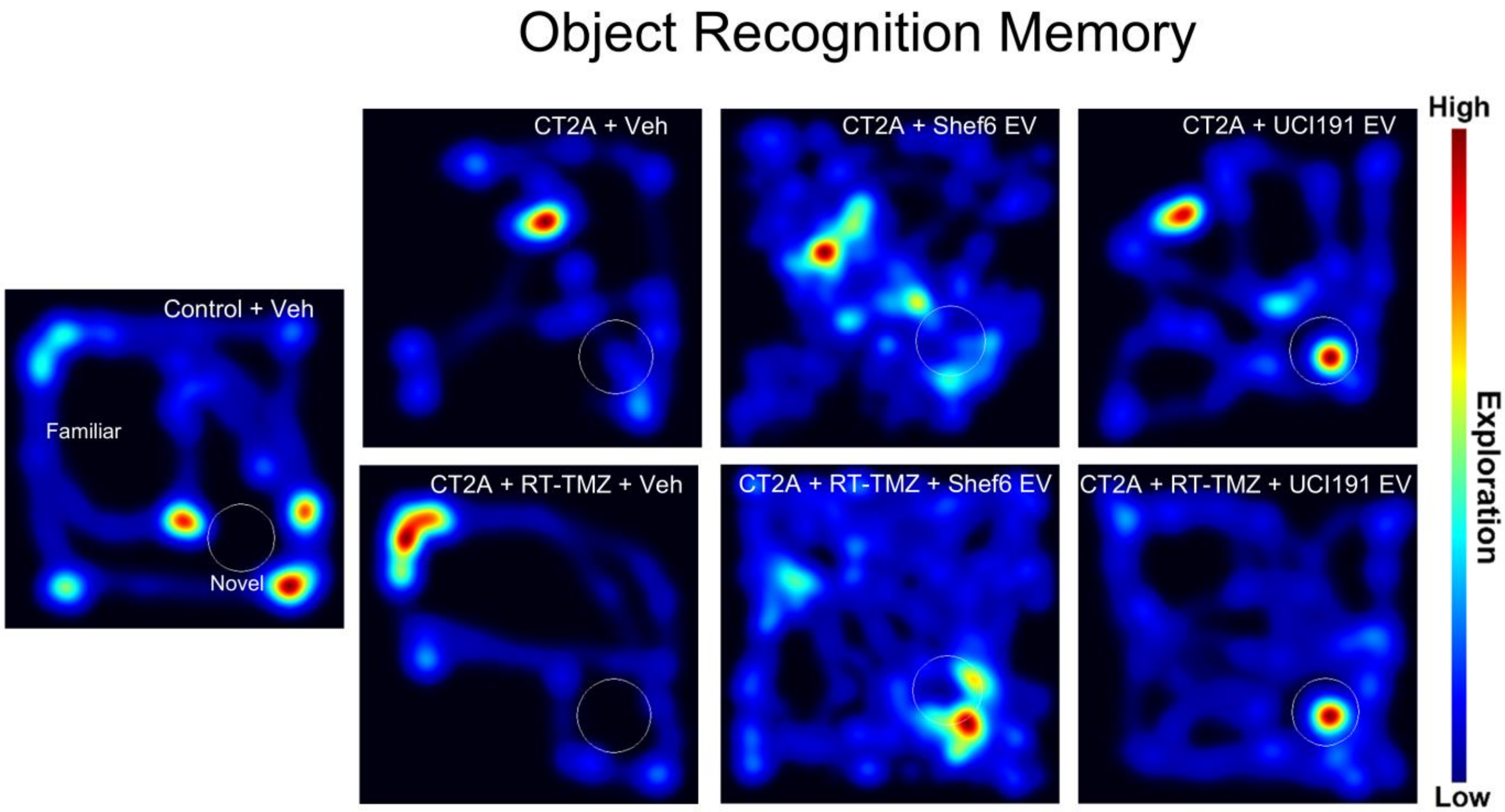

Object Recognition Memory
Control + Veh
Familiar
Novel
CT2A + Veh
CT2A + Shef6 EV
CT2A + UCI191 EV
CT2A + RT-TMZ + Veh
CT2A + RT-TMZ + Shef6 EV
CT2A + RT-TMZ + UCI191 EV
High
Exploration
Low

Supplemental Figure S8.

Representative heatmaps of exploration in Object Recognition Memory in glioma-bearing mice.

Representative heatmaps depicting exploration throughout the object recognition memory (ORM) test for the data presented in **Figure 5E.**

Suppl. Fig. S9

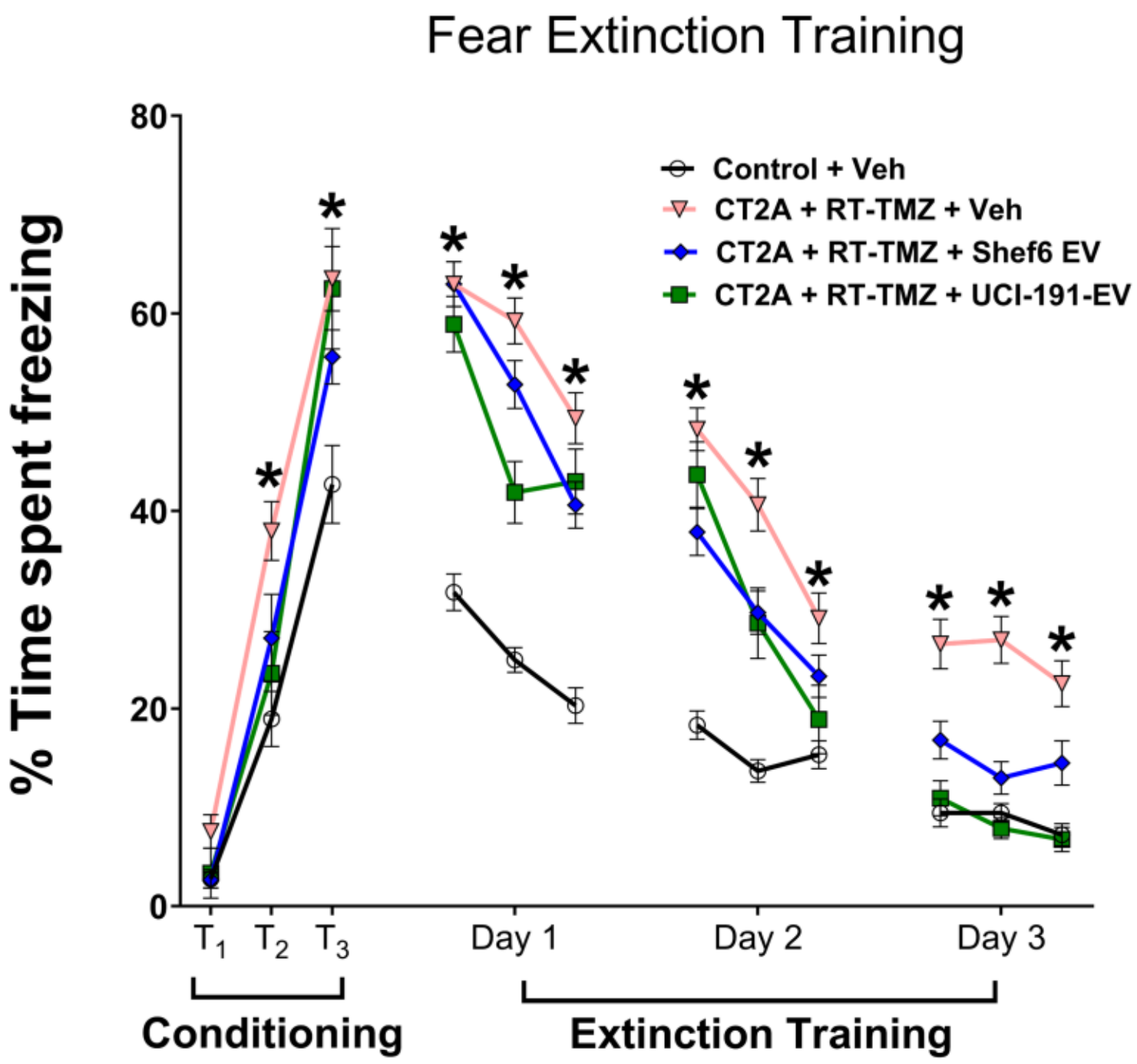
Fear Extinction Training
% Time spent freezing
80
60
40
20
0
Control + Veh
CT2A + RT-TMZ + Veh
CT2A + RT-TMZ + Shef6 EV
CT2A + RT-TMZ + UCI-191-EV
T1
T2
T3
Day 1
Day 2
Day 3
Conditioning
Extinction Training

Supplemental Figure S9.

Effects of combined cranial radiotherapy and chemotherapy, or hNSC-derived EVs in glioma-bearing mice, on fear conditioning and extinction.

Graphical depiction of sequential fear extinction (*Days 1-3*) after the initial fear conditioning phase (*T1-3*). Successful extinction of the conditioned fear response is evidenced by a decrease in the percentage of time spent freezing. Analysis of the percentage of time spent freezing on the final Test Day of fear extinction (FE) is depicted in **Figures 5F**.

Data are represented by mean ± SEM (N = 4-12 animals/group). *P* values were derived from two-way ANOVA and Bonferroni's multiple comparisons tests. *, $P \leq 0.05$ vs. Control + Veh group.

Suppl. Fig. S10

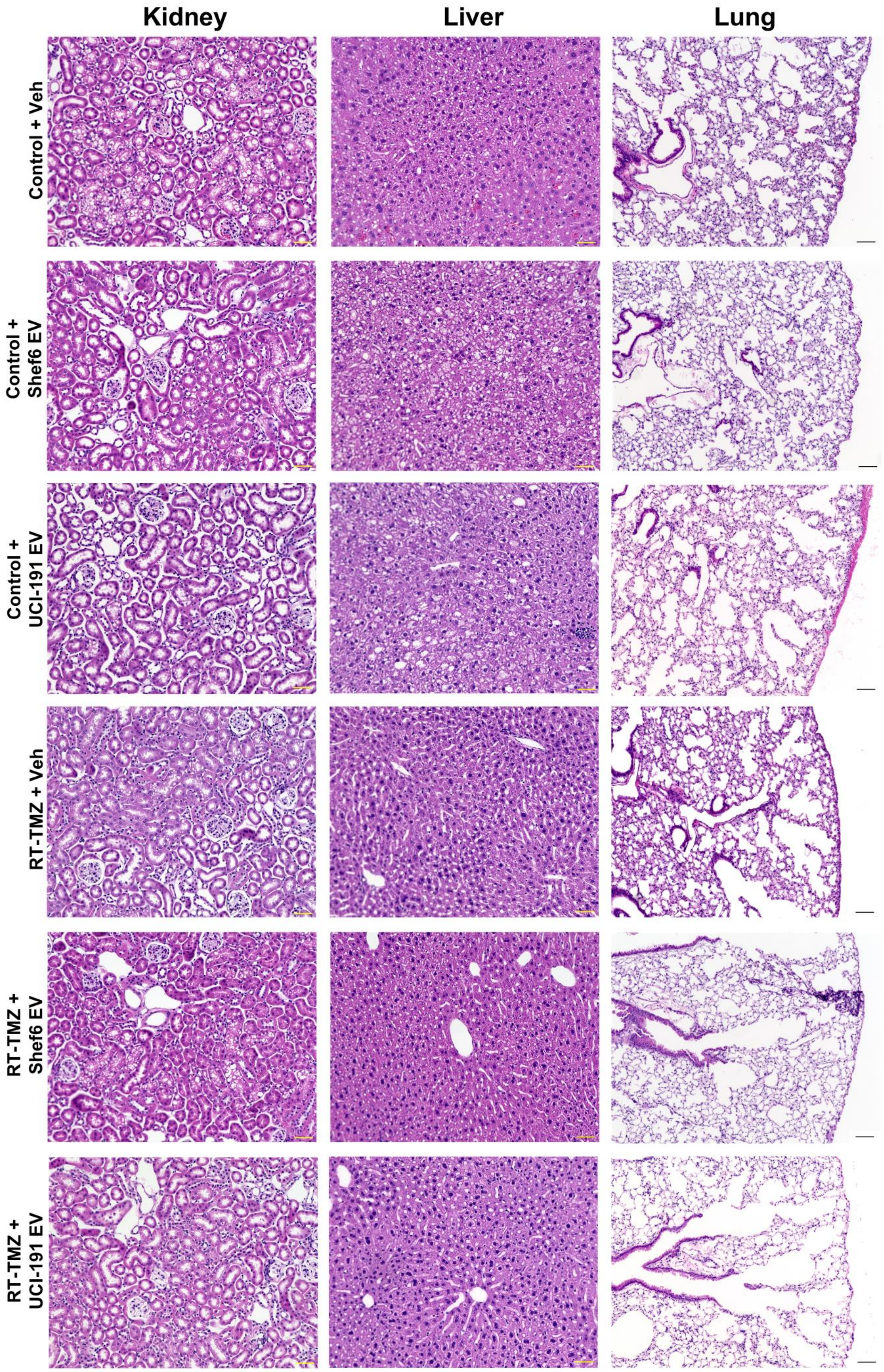

Supplemental Figure S10.

Histopathology of peripheral organs across treatment groups in males.

Representative H&E images for non-tumor-bearing male mice. Kidney, liver, and lung tissue specimens were collected and evaluated for gross morphology following hNSC-EV treatment. Paraffin-embedded tissue slices were stained for hematoxylin and eosin (H&E).

Kidney and liver were imaged at 20x magnification, and the lung was imaged at 10x magnification. Scale bars, 50 μm for 20x images and 100 μm for 10x.

Suppl. Fig. S11

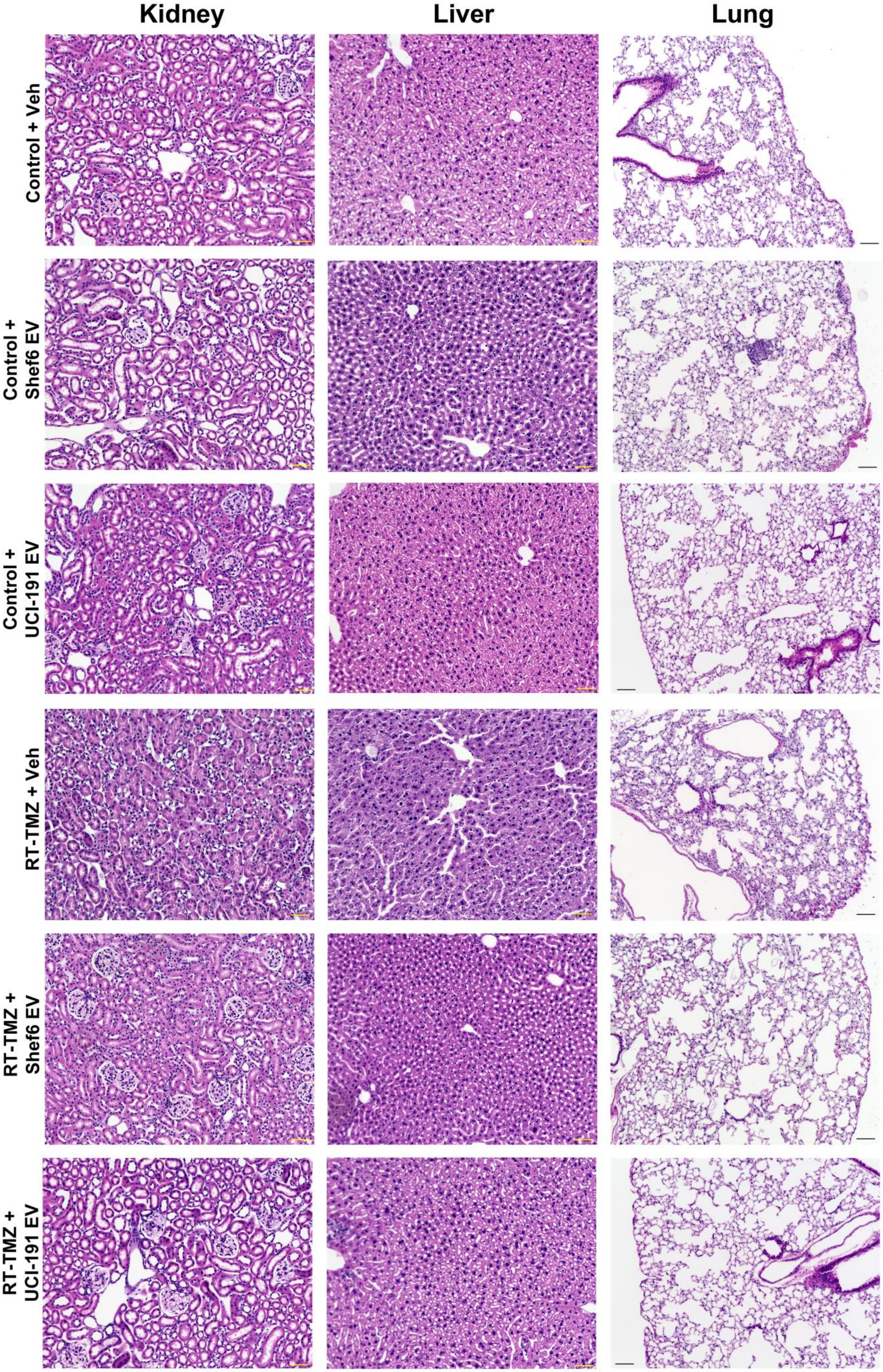

Supplemental Figure S11.

Histopathology of peripheral organs across treatment groups in females.

Representative H&E images for non-tumor-bearing female mice. Kidney, liver, and lung tissue specimens were collected and evaluated for gross morphology following hNSC-EV treatment. Paraffin-embedded tissue slices were stained for hematoxylin and eosin (H&E).

Kidney and liver were imaged at 20x magnification, and the lung was imaged at 10x magnification. Scale bars, 50 μm for 20x images and 100 μm for 10x.

Suppl. Fig. S12

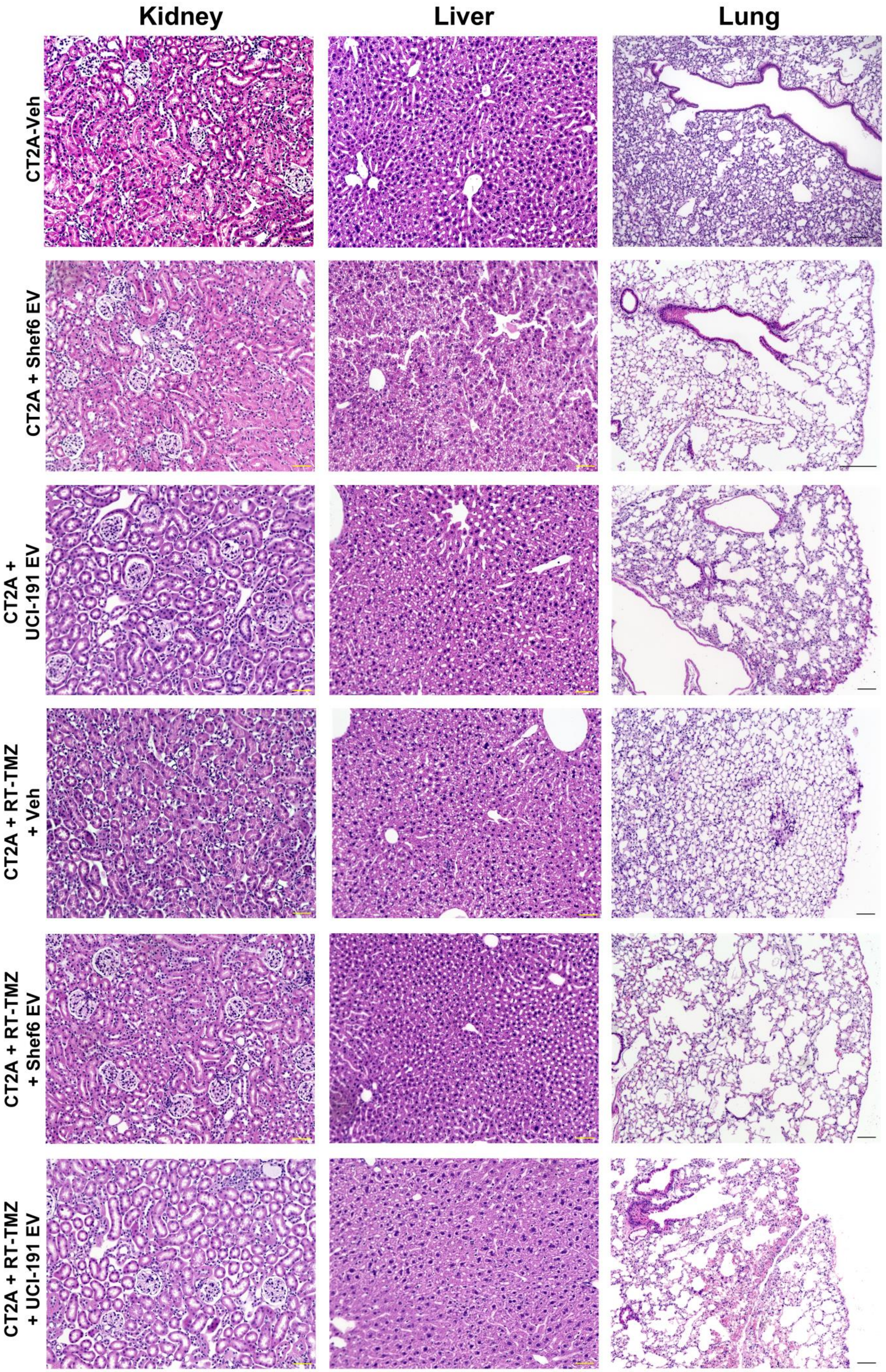

Supplemental Figure S12.

Histopathology of peripheral organs across treatment groups in glioma-bearing animals.

Representative H&E images for mice induced with CT2A-glioma. Kidney, liver, and lung tissue specimens were collected and evaluated for gross morphology following hNSC-EV treatment. Paraffin-embedded tissue slices were stained for hematoxylin and eosin (H&E).

Kidney and liver were imaged at 20x magnification, and the lung was imaged at 10x magnification. N = 2 females and 4 males. Scale bars, 50 μm for 20x images and 100 μm for 10x.

Suppl. Table T1

Summary of significant differences for the data as presented in Figure 5E.

Compared Groups	Significance	P-Value
Control + Veh vs. CT2A + Veh	**	0.0053
Control + Veh vs. CT2A + Shef6 EV	***	0.0008
Control + Veh vs. CT2A + UCI-191 EV	***	0.0002
Control + Veh vs. CT2A + RT-TMZ + Veh	*	0.0382
CT2A + Veh vs. CT2A + RT-TMZ + Shef6 EV	*	0.0132
CT2A + Veh vs. CT2A + RT-TMZ + UCI-191 EV	**	0.0017
CT2A + Shef6 EV vs. CT2A + RT-TMZ + Shef6 EV	**	0.0019
CT2A + Shef6 EV vs. CT2A + RT-TMZ + UCI-191 EV	***	0.0003
CT2A + UCI-191 EV vs. CT2A + RT-TMZ + Shef6 EV	***	0.0004
CT2A + UCI-191 EV vs. CT2A + RT-TMZ + UCI-191 EV	****	≤ 0.0001
CT2A + RT-TMZ + Veh vs. CT2A + RT-TMZ + UCI-191 EV	*	0.0126

The data presented in **Figure 5E** were for object recognition memory (ORM) in glioma-bearing mice. ORM data were calculated as a Preference Index as described in the Methods and Materials. Groups that are underlined had a higher Preference Index. Data are presented as mean ± SEM (N = 5-12 animals/group). *P* values were derived from two-way ANOVA and Bonferroni's multiple comparisons.

Suppl. Table T2

Summary of significant differences for the data as presented in Figure 6C.

Compared Groups	Significance	P Value
Control + Veh vs. CT2A + Veh	****	≤ 0.0001
Control + Veh vs. CT2A + UCI-191 EV	****	≤ 0.0001
Control + Veh vs. CT2A + RT-TMZ + Veh	**	0.0093
CT2A + Veh vs. CT2A + Shef6 EV	****	≤ 0.0001
CT2A + Veh vs. CT2A + RT-TMZ + Veh	***	0.0001
CT2A + Veh vs. CT2A + RT-TMZ + Shef6 EV	****	≤ 0.0001
CT2A + Veh vs. CT2A + RT-TMZ + UCI-191 EV	****	≤ 0.0001
CT2A + Shef6 EV vs. CT2A + UCI-191 EV	***	0.0004
CT2A + Shef6 EV vs. CT2A + RT-TMZ + Shef6 EV	**	0.0013
CT2A + UCI-191 EV vs. CT2A + RT-TMZ + Veh	*	0.0183
CT2A + UCI-191 EV vs. CT2A + RT-TMZ + Shef6 EV	****	≤ 0.0001
CT2A + UCI-191 EV vs. CT2A + RT-TMZ + UCI-191 EV	****	≤ 0.0001
CT2A + RT-TMZ + Veh vs. CT2A + RT-TMZ + Shef6 EV	****	≤ 0.0001

The data presented in **Figure 6C** were for the volumetric analysis of the astrocytic glial fibrillary acidic protein (GFAP) in glioma-bearing mice. Groups that are underlined had a higher GFAP expression. Data are presented as mean ± SEM (N = 5-12 animals/group). *P* values were derived from one-way ANOVA and Tukey's multiple comparisons tests.

Suppl. Table T3

Summary of significant differences for the data as presented in Figure 6F.

Compared Groups	Significance	P Value
Control + Veh vs. CT2A + Veh	****	≤ 0.0001
Control + Veh vs. CT2A + UCI-191 EV	****	≤ 0.0001
Control + Veh vs. CT2A + RT-TMZ + Veh	**	0.0026
Control + Veh vs. CT2A + RT-TMZ + Shef6 EV	*	0.0328
CT2A + Veh vs. CT2A + Shef6 EV	****	≤ 0.0001
CT2A + Veh vs. CT2A + RT-TMZ + Veh	****	≤ 0.0001
CT2A + Veh vs. CT2A + RT-TMZ + Shef6 EV	****	≤ 0.0001
CT2A + Veh vs. CT2A + RT-TMZ + UCI-191 EV	****	≤ 0.0001
CT2A + Shef6 EV vs. CT2A + UCI-191 EV	****	≤ 0.0001
CT2A + UCI-191 EV vs. CT2A + RT-TMZ + Veh	***	0.0002
CT2A + UCI-191 EV vs. CT2A + RT-TMZ + Shef6 EV	***	0.0002
CT2A + UCI-191 EV vs. CT2A + RT-TMZ + UCI-191 EV	****	≤ 0.0001
CT2A + RT-TMZ + Veh vs. CT2A + RT-TMZ + UCI-191 EV	*	0.0367

The data presented in **Figure 6F** were for the volumetric analysis of the colocalization of the ionized calcium-binding adaptor molecule 1 (Iba1), and lysosomal protein CD68.Groups that are underlined had a higher CD68-Iba1 co-localization. Data are presented as mean ± SEM (N = 5-12 animals/group). *P* values were derived from one-way ANOVA and Tukey's multiple comparisons tests.

Suppl. Table T4

Summary of significant differences for the data as presented in Figure 7E.

Compared Groups	Significance	P Value
Control + Veh vs. CT2A + Veh	****	≤ 0.0001
Control + Veh vs. CT2A + Shef6 EV	****	≤ 0.0001
Control + Veh vs. CT2A + UCI-191 EV	****	≤ 0.0001
Control + Veh vs. CT2A + RT-TMZ + Veh	****	≤ 0.0001
Control + Veh vs. CT2A + RT-TMZ + Shef6 EV	*	0.015
CT2A + Veh vs. CT2A + Shef6 EV	****	≤ 0.0001
CT2A + Veh vs. CT2A + UCI-191 EV	**	0.0095
CT2A + Veh vs. CT2A + RT-TMZ + Veh	**	0.0047
CT2A + Veh vs. CT2A + RT-TMZ + Shef6 EV	****	≤ 0.0001
CT2A + Veh vs. CT2A + RT-TMZ + UCI-191 EV	****	≤ 0.0001
CT2A + Shef6 EV vs. CT2A + UCI-191 EV	****	≤ 0.0001
CT2A + Shef6 EV vs. CT2A + RT-TMZ + Shef6 EV	****	≤ 0.0001
CT2A + Shef6 EV vs. CT2A + RT-TMZ + UCI-191 EV	****	≤ 0.0001
CT2A + UCI-191 EV vs. CT2A + RT-TMZ + Veh	****	≤ 0.0001
CT2A + UCI-191 EV vs. CT2A + RT-TMZ + Shef6 EV	****	≤ 0.0001
CT2A + UCI-191 EV vs. CT2A + RT-TMZ + UCI-191 EV	****	≤ 0.0001
CT2A + RT-TMZ + Veh vs. CT2A + RT-TMZ + Shef6 EV	****	≤ 0.0001
CT2A + RT-TMZ + Veh vs. CT2A + RT-TMZ + UCI-191 EV	****	≤ 0.0001

The data presented in **Figure 7E** were for the volumetric analysis of synaptic vesicle glycoprotein 2A (SV2a) in the pyramidal cell layer (*pyr*) and stratum radiatum (*sr*) of CA1 in the hippocampus of glioma-bearing mice. Groups that are underlined had a higher SV2a expression. Data are presented as mean ± SEM (N = 5-12 animals/group). *P* values were derived from one-way ANOVA and Tukey's multiple comparisons tests.

Suppl. Table T5

Summary of significant differences for the data as presented in Figure 7F.

Compared Groups	Significance	P Value
Control + Veh vs. CT2A + Veh	****	≤ 0.0001
Control + Veh vs. CT2A + Shef6 EV	****	≤ 0.0001
Control + Veh vs. CT2A + UCI-191 EV	*	0.0181
Control + Veh vs. CT2A + RT-TMZ + Veh	****	≤ 0.0001
CT2A + Veh vs. CT2A + Shef6 EV	**	0.0043
CT2A + Veh vs. CT2A + UCI-191 EV	****	≤ 0.0001
CT2A + Veh vs. CT2A + RT-TMZ + Shef6 EV	****	≤ 0.0001
CT2A + Veh vs. CT2A + RT-TMZ + UCI-191 EV	****	≤ 0.0001
CT2A + Shef6 EV vs. CT2A + UCI-191 EV	***	0.0003
CT2A + Shef6 EV vs. CT2A + RT-TMZ + Shef6 EV	****	≤ 0.0001
CT2A + Shef6 EV vs. CT2A + RT-TMZ + UCI-191 EV	****	≤ 0.0001
CT2A + UCI-191 EV vs. CT2A + RT-TMZ + Veh	****	≤ 0.0001
CT2A + UCI-191 EV vs. CT2A + RT-TMZ + UCI-191 EV	****	≤ 0.0001
CT2A + RT-TMZ + Veh vs. CT2A + RT-TMZ + Shef6 EV	****	≤ 0.0001
CT2A + RT-TMZ + Veh vs. CT2A + RT-TMZ + UCI-191 EV	****	≤ 0.0001
CT2A + RT-TMZ + Shef6 EV vs. CT2A + RT-TMZ + UCI-191 EV	***	0.001

The data presented in **Figure 7F** were for the volumetric analysis of synaptic vesicle glycoprotein 2A (SV2a) in the molecular layer (*ml*) and dentate gyrus (*dg*) in the hippocampus of glioma-bearing mice. Groups that are underlined had a higher SV2a expression. Data are presented as mean ± SEM (N = 5-12 animals/group). *P* values were derived from one-way ANOVA and Tukey's multiple comparisons tests.

Suppl. Table T6

Summary of significant differences for the data as presented in Figure 7G.

Compared Groups	Significance	P Value
Control + Veh vs. CT2A + Veh	****	≤ 0.0001
Control + Veh vs. CT2A + Shef6 EV	****	≤ 0.0001
Control + Veh vs. CT2A + UCI-191 EV	****	≤ 0.0001
Control + Veh vs. CT2A + RT-TMZ + Veh	****	≤ 0.0001
CT2A + Veh vs. CT2A + RT-TMZ + Shef6 EV	****	≤ 0.0001
CT2A + Veh vs. CT2A + RT-TMZ + UCI-191 EV	****	≤ 0.0001
CT2A + Shef6 EV vs. CT2A + UCI-191 EV	*	0.0338
CT2A + Shef6 EV vs. CT2A + RT-TMZ + Shef6 EV	**	0.001
CT2A + Shef6 EV vs. CT2A + RT-TMZ + UCI-191 EV	**	0.0056
CT2A + UCI-191 EV vs. CT2A + RT-TMZ + Shef6 EV	****	≤ 0.0001
CT2A + UCI-191 EV vs. CT2A + RT-TMZ + UCI-191 EV	****	≤ 0.0001
CT2A + RT-TMZ + Veh vs. CT2A + RT-TMZ + Shef6 EV	**	0.0013
CT2A + RT-TMZ + Veh vs. CT2A + RT-TMZ + UCI-191 EV	**	0.006

The data presented in **Figure 7G** were for the volumetric analysis of postsynaptic density protein 95 (PSD95) in the pyramidal cell layer (*pyr*) and stratum radiatum (*sr*) of CA1 in the hippocampus of glioma-bearing mice. Groups that are underlined had a higher PSD95 expression. Data are presented as mean ± SEM (N = 5-12 animals/group). *P* values were derived from one-way ANOVA and Tukey's multiple comparisons tests.

Suppl. Table T7

Summary of significant differences for the data as presented in Figure 7H.

Compared Groups	Significance	P Value
Control + Veh vs. CT2A + Veh	***	0.0002
Control + Veh vs. CT2A + UCI-191 EV	**	0.009
Control + Veh vs. CT2A + RT-TMZ + UCI-191 EV	*	0.0301
CT2A + Veh vs. CT2A + Shef6 EV	*	0.0321
CT2A + Veh vs. CT2A + UCI-191 EV	****	≤ 0.0001
CT2A + Veh vs. CT2A + RT-TMZ + Veh	*	0.0117
CT2A + Veh vs. CT2A + RT-TMZ + Shef6 EV	****	≤ 0.0001
CT2A + Veh vs. CT2A + RT-TMZ + UCI-191 EV	****	≤ 0.0001
CT2A + Shef6 EV vs. CT2A + UCI-191 EV	***	0.0001
CT2A + Shef6 EV vs. CT2A + RT-TMZ + UCI-191 EV	***	0.0005
CT2A + UCI-191 EV vs. CT2A + RT-TMZ + Veh	**	0.0011
CT2A + RT-TMZ + Veh vs. CT2A + RT-TMZ + UCI-191 EV	**	0.0038

The data presented in **Figure 7H** were for the volumetric analysis of postsynaptic density protein 95 (PSD95) in the molecular layer (*ml*) and dentate gyrus (*dg*) in the hippocampus of glioma-bearing mice. Groups that are underlined had a higher PSD95 expression. Data are presented as mean ± SEM (N = 5-12 animals/group). *P* values were derived from one-way ANOVA and Tukey's multiple comparisons tests.

SUPPLEMENTAL RESOURCE TABLE

REAGENT or RESOURCES	SOURCE	IDENTIFIER
**Antibodies**		
Rat anti-Mouse CD68	Bio-Rad	Cat: MCA1957<br>RRID: AB_322219
Rabbit anti-IBA1	FUJIFILM Wako	Cat: 019-19741<br>RRID: AB_839504
Goat anti-Rat AF 647	Abcam	Cat: ab150159<br>RRID: AB_2566823
Goat anti Rabbit AF 488	FisherSci	Cat: a11008<br>RRID: AB_143165
Mouse anti-SV2A	DHSB	Cat: SV2<br>RRID: AB_2315387
Goat anti-Mouse AF 568	Abcam	Cat: ab175473<br>RRID: AB_2895153
Mouse anti-PSD-95	FisherSci	Cat: MA1-045<br>RRID: AB_325399
Goat anti-Mouse AF 647	Abcam	Cat: ab150115<br>RRID: AB_2687948
Mouse anti-GFAP	Milipore	Cat: AB5804<br>RRID: AB_11212597
DAPI	FisherSci	Cat: D1306<br>RRID: AB_2629482
**Experimental Model: Cell lines**		
CT2A-Luc astrocytoma cells	Sigma-Aldrich	Cat: SCC195<br>RRID: CVCL_ZJ60
Human neural stem cells UCI-191	Univ. of California Irvine	Dr. Aileen Anderson (PI)
Human neural stem cells Shef6	Univ. of California Irvine	Dr. Brian Cummings (PI)
Contd.		
**Experimental Model: Organisms/strains**		
C57BL/6J mice	Jackson Labs	Cat: 000664<br>RRID: IMSR_JAX:000664
**Chemicals, peptides, and recombinant proteins**		
Gibco™ DMEM (1X) + GlutaMAX™-I	FisherSci	Cat: 10-569-010
Gibco™ FBS	FisherSci	Cat: 10-082-147
Gibco™ Hibernate™-A Medium	FisherSci	Cat: A1247501
Gibco™ TrypLE™ Express Enzyme	FisherSci	Cat: 12-605-010
CTS™ TrypLE™ Select Enzyme	ThermoFisher	Cat: A1285901
Isospire™ (isoflurane) Inhalation Anesthetic	Dechra	Cat: 26675-46-7
Betadine® Surgical Scrub	Patterson Veterinary	Cat: 07-836-3379
Hydrogen Peroxide, 30% (Certified ACS)	FisherSci	Cat: H325-500
Normal Saline (Sodium Chloride) 0.9%	Patterson Veterinary	Cat: 07-800-9424
Buprenorphine HCl (0.3 mg/ml)	MWI Health	Cat: 78949214
VECTASHIELD® Antifade Mounting Medium	VectaShield	Cat: H-1000-10
D-Luciferin, sodium salt	GoldBio	Cat: eLUCNA
Gibco™ DPBS (no magnesium, no calcium)	FisherSci	Cat: 14-190-144
Normal Goat Serum	Jackson ImmunoReasearch	Cat: 005-000-121<br>RRID: AB_2336990
Bovine Serum Albumin	Sigma-Aldrich	Cat: 05470
Virkon, premixed spray bottle	Lanxess	Cat: RSL_31/Virks
Heparin sodium salt from porcine intestinal mucosa	Sigma-Aldrich	Cat: H3149-25KU

Gibco™ DPBS (10X), no calcium, no magnesium	FisherSci	Cat: 14-190-144
Gibco™ DPBS (10X), with calcium and magnesium	FisherSci	Cat: 14-040-117
Temozolomide	Selleck Chemicals	Cat: S1237
Human FGF-acidic (FGF-1) Recombinant Protein	ThermoFisher	Cat: PHG0014
Human EGF Recombinant Protein	ThermoFisher	Cat: PHG0311
CTS™ N-2 Supplement	Invitrogen	Cat: A13707-01
Leukemia Inhibitory Factor (LIF)	Sigma-Aldrich	Cat: LIF1010
N-acetylcysteine	Sigma-Aldrich	Cat: A9165-5G
X-VIVO™15 Serum-Free Medium	Lonza	Cat: 04-481Q
CELLStart™ Substrate	ThermoFisher	Cat: A1014201
Methanol	FisherSci	Cat: A412500
Paraformaldehyde	Sigma-Aldrich	Cat: 158127
Sodium azide	Sigma-Aldrich	Cat: S2002
Sucrose	Sigma-Aldrich	Cat: S7903
Tissue-Tek* O.C.T. Compound	VWR	Cat: 25608-930
Tris-buffered Salin (10X TBS)	Bioland Scientific	Cat: TBS01-03
Triton™ X-100 Surfact-Amps™ Detergent Solution	FisherSci	Cat: 85111
TWEEN® 20	Sigma-Aldrich	Cat: 9005-64-5
FineFIX	Milestone Medical	Cat: 70147G
**Critical commercial assays**		
RNeasy Plus Universal Mini Kit	Qiagen	Cat: 73404
Nanostring nCounter Mouse Neuroinflammation	Nanostring	Cat: XT-CSO-MNROI1-12
**Software and algorithms**		
Living Image Software	Revvity	Cat: 128110
IVIS Lumina III Imaging System	Revvity	Cat: CLS136334
Noldus EthoVisionXT 18 Tracking Software	Noldus	RRID: SCR_000441
FreezeFrame v6 (ActiMetrics)	Coulbourn Instruments	RRID: SCR_014429
Imaris 9.1	Oxford Instruments	RRID: SCR_007370
nSolver software version 4.0	Nanostring	N/A
nSolver Advanced Analysis	Nanostring	N/A
NanoSight 300	Malvern Panalytical	Cat: NS300
**Other**		
Ultra Precise Digital Just for Mouse Stereotaxic Instrument	Stoelting	Cat: 51730UD
Sterile Alcohol Prep Pads	FisherSci	Cat: 06-669-62
Feather™ Single-Use Scalpels, size #11	FisherSci	Cat: 08-927-5B
10 µL Microliter Syringe Model 701 N	Hamilton	Cat: 80300
Pivetal® WebCryl™ Sutures	Patterson Veterinary	Cat: 07-891-0161
3M™ Vetbond™ Tissue Adhesive Bottle	Patterson Veterinary	Cat: 07-805-5031
Fisherbrand™ Superfrost™ Plus Microscope Slides	FisherSci	Cat: 22-037-246
SmART+ irradiator	Precision X-Ray	RRID: SCR_021996
Noldus EthoVisionXT 18 Video Capture Hardware System	Noldus	RRID: SCR_000441
Nanodrop	FisherSci	Cat: 13-400-525
Bone Drill	Cell Point Scientific	Cat: 67-1024
Anesthesia System	Vet Equip	Cat: 922100
Optima XE-90 Ultracentrifuge	Beckman Coulter	Cat: A94471
0.2 µm Nalgene™ Rapid-Flow™ Sterile Filter Unit	ThermoFisher	Cat: 566-0020
0.2 µm Nylon Filter	ThermoFisher	Cat: 13-1001-00
70 mL ultracentrifuge tubes	Beckman Coulter	Cat: 355655
26 mL ultracentrifuge tubes	Beckman Coulter	Cat: 355654
Transmission Electron Microscope	JOEL TLD	Cat: Jem-1400 Plus

UCSD Cellular and Molecular Medicine Electron Microscopy Core	University of California, San Diego	RRID: SCR_022039
UCI Genomics Research and Technology Hub	Univ. of California, Irvine	RRID: SCR_026615
UCI Experimental Tissue Resources	Univ. of California, Irvine	RRID: SCR_024622
Cryostat Hm 525 NX	Cryostat MX	Cat: 95-664-0EC
